# Frustration reduces interpersonal competition through dynamic interpersonal neural synchronization in dyads

**DOI:** 10.1038/s42003-026-10573-6

**Published:** 2026-06-27

**Authors:** Jingyue Liu, Yujia Meng, Chenyu Zhang, Ye Zhang, Yifei Zhong, Rui Huang, Yingjie Liu

**Affiliations:** 1 School of Psychology and Mental Health, Hebei Key Laboratory of Mental Health and Brain Science, North China University of Science and Technology, Tangshan, China; 2 Key Laboratory of Modern Teaching Technology, Ministry of Education, Shaanxi Normal University, Xi’an, China

**Keywords:** Human behaviour, Prefrontal cortex, Decision

## Abstract

Frustration reduces competitive willingness in social interactions, yet the underlying dynamic neural mechanisms remain unclear. Using functional near-infrared spectroscopy (fNIRS) hyperscanning, we record dyadic hemodynamic activity during a competitive task following a frustration task. Results show that frustration progressively reduces competitive willingness. Accordingly, increased interpersonal neural synchronization (INS) is observed in the frontopolar cortex (FPC) during the middle stage and the dorsolateral prefrontal cortex (dlPFC) during the late stage. FPC INS mediates the effect of frustration on competition performance. Role differences are determined based on frustration task performance, and Granger causality analysis reveals a bidirectional influence that is significantly stronger from leaders to followers. These findings suggest that frustration enhances cognitive control and executive function in the middle stage through increased INS in FPC, and enhances inference of others’ intentions in the late stage through increased INS in dlPFC, thereby dynamically reducing competition. This study reveals a distinct neural pattern of interpersonal competition processes under frustration.

## Abstract (web summary)

fNIRS hyperscanning reveals that frustration reduces competitive willingness over time, with frontopolar interpersonal neural synchronization in the middle stage and dorsolateral prefrontal synchronization in the late stage.

## Introduction

Within the intricate social interaction network, interpersonal competition, as a fundamental mode of social interaction, serves as a crucial driving force for personal growth and societal advancement, with its internal mechanisms and external manifestations being prominent research topics in psychology^1^. Traditional perspectives often characterize competition as a zero-sum game, wherein clashes among individuals or groups revolve around vying for finite resources, frequently accompanied by heightened stress, discord, and at times, animosity^2,3^. With advances in social psychology, researchers have increasingly focused on the complexity of interpersonal competitive behavior, particularly examining how various factors influence individuals’ competitive strategies^4^. Among these factors, although the potential impact of frustration as a significant contributor to interpersonal competition is evident, it has yet to garner widespread attention^5^. It pervades all aspects of daily life and is considered a barrier to the realization of anticipated satisfaction^5,6^. Frustration not only triggers negative emotional responses, such as anxiety and anger^7^, but also alters an individual’s mental state and behavioral patterns, ultimately affecting their performance in social interactions^8,9^. Hence, it holds profound significance to unveil the patterns of human interpersonal competitive responses after frustrating situations and their underlying neural mechanisms.

Previous research on the relationship between frustration and competitive behavior has yielded contradictory conclusions. On one hand, some scholars posit that frustration can activate an individual’s psychological resilience mechanisms, motivating individuals to exhibit heightened competitive drive and greater effort to overcome obstacles^10,11^. On the other hand, research suggests that frustration primarily acts as a potent psychological stressor, triggering psychological defense mechanisms^9,12^. This discrepancy may stem from the nature of the task paradigm and the degree of psychological resource depletion: when frustration is perceived as a surmountable challenge, it may trigger resilience; however, in more complex interactive settings, frustration often significantly constricts cognitive bandwidth. Increased psychological strain depletes the cognitive resources required to execute sophisticated competitive strategies^13,14^. In such contexts, individuals tend to prioritize psychological equilibrium and self-preservation mechanisms over competitive victory, thereby exhibiting an avoidance of competitive scenarios^15^. Therefore, considering the complexity of social interaction, we hypothesize that individuals experiencing frustration will exhibit diminished competitive willingness in rapid key-pressing tasks in subsequent interpersonal competitive tasks.

Hyperscanning technology provides a critical perspective for uncovering the neural mechanisms underlying changes in interpersonal competitive behavior^16^. Previous fNIRS-based hyperscanning studies have shown that in typical competitive interaction contexts, competing partners exhibit significant interpersonal neural synchronization (INS) in the right medial prefrontal cortex (mPFC). This synchronization is thought to reflect a strategic game process based on mutual prediction between interacting individuals^17^. However, when psychological stressors intervene, distinct relationship patterns may emerge between interpersonal competitive behavior and neural synchronization. For example, a study involving young women found that acute psychosocial stress significantly suppressed overt competitive behavior, while a significant increase in INS was observed in the right dorsolateral prefrontal cortex (dlPFC)^18^. Similarly, another study showed that when individuals faced a mortality threat, although their competitive behavior decreased, neural synchronization between interacting individuals was enhanced^19^. Therefore, under challenging conditions, individuals may trigger a compensatory neural coupling mechanism. Specifically, psychological stressors may weaken competitive behavior interacting individuals, while interacting partners instead exhibit higher INS. In such cases, the increased INS may reflect a highly integrated but less efficient interaction state, in which individuals need to invest more neural resources to maintain the interaction process, yet this integration does not necessarily correspond to higher behavioral efficiency^19,20^.

However, despite current advances in fNIRS hyperscanning technology, the assessment of INS remains limited by an implicit assumption—namely, that neural activity exhibits temporal stationarity throughout the measurement period^21^. Most existing studies tend to average data across the entire task duration to evaluate INS^22,23^. In contrast, interpersonal competition is essentially a highly dynamic social interaction process involving complex, real-time adjustments of emotional, cognitive, and social strategies^2,24^. Furthermore, from a hyperscanning perspective, the interaction patterns between two participants after jointly experiencing frustration may exhibit dynamic characteristics that are distinct from those in single-person contexts. Research has indicated that jointly experiencing frustration not only influences individual emotional regulation through social referencing^25,26^, but also prompts participants to gradually adjust their goal expectations and motivational structures^12,27^. Such shared experiences may trigger an adaptive psychological adjustment process: as frustration persists, individuals may gradually shift from their initial competitive focus toward maintaining psychological equilibrium, adopting more conservative behavioral strategies^7,15^. Therefore, the impact of frustration on interpersonal competition is not a monolithic instantaneous effect, but rather a dynamic mitigation process encompassing multi-stage changes^28^. This perspective, focusing on ecological validity and temporal dynamics, aligns closely with recent academic calls to evaluate social interaction behavior in real-world contexts^29,30^. In summary, to more accurately and comprehensively understand the dynamic neural synchronization mechanisms during interpersonal competition under frustration, the present study employs a more refined approach to explore the dynamic fluctuations of INS throughout the competitive task.

Notably, in previous research on the neural synchronization mechanisms of interpersonal competition, two competing participants are usually considered equal and symmetrical^16^, while relatively little attention has been paid to role asymmetry, which may overlook the role differences that prevail in the real world^2,22^. Indeed, research on leadership has found that leader-follower relationships spontaneously arise between participants in leaderless group discussions, even when they do not differ in natural or social status^31^. In addition, when a group faces frustration, the member who makes the final decision and assumes responsibility for the outcome is more likely to be identified as the relative leader^32,33^, and thus interpersonal neural computation with role symmetry alone may mask some important information. In the present study, relative roles (leader or follower) were assigned based on dyads’ interaction performance during the frustration task. This allowed us to examine interaction patterns during interpersonal competition involving asymmetric roles. Granger causality analysis (GCA) was able to provide neurobiological cues for the present study as to which individual more actively drives the other^34^. We therefore used GCA to further examine the coupled directionality of information flow between roles during competition.

In summary, this study will explore the dynamic effects of frustration on interpersonal competition using fNIRS hyperscanning. During the experiment, two participants cooperate to complete an adapted frustration-inducing task^7^. We collected corresponding emotional and psychological indicators via the Positive and Negative Affect Schedule (PANAS)^35^ and confidence levels to verify whether the induction was successful. In addition, relative leader and follower roles were assigned based on participants’ responses to the task questions and their performance during the frustration task. The classic keypress task was used to assess interpersonal competition performance^16^. During the interpersonal competition task, fNIRS simultaneously recorded the prefrontal cortex (PFC) of both participants. This region plays an important role in competitive interactions^36^. Studies have shown that the PFC is associated with basic cognitive abilities such as attention^37^ and working memory^38^, as well as with social cognitive abilities such as cognitive control^39^, which are important in keypress tasks. In addition, a significant increase in INS has been observed in PFC in several interpersonal competitive hyperscanning studies^17,36^. Therefore, we used the PFC as the region of interest. We hypothesized that: i) INS would be increased in the frustration condition (FC) relative to the control condition (CC). ii) Frustration dynamically reduces interpersonal competition across temporal phases. Dynamic INS in PFC would be strongly correlated with interpersonal competition behaviors at the corresponding time points. iii) The leader-follower dynamic during interpersonal competition interactions is bidirectional, with the leader eliciting more neural activity in the follower.

## Results

### Validity checks in frustration induction

**Fig. 1:**
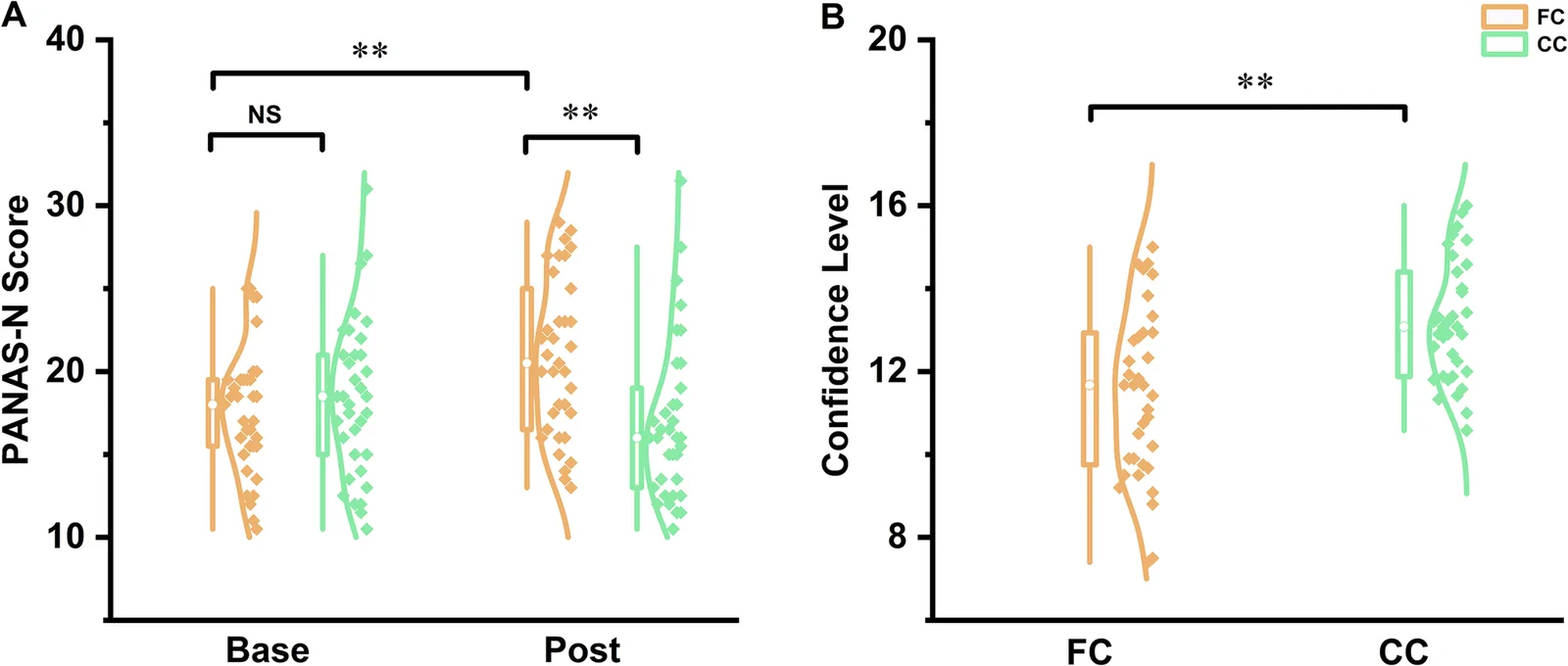
Behavioral and psychological validation of the frustration induction (*n* = 70). **A** Negative affect scores. The violin plots illustrate the Negative Affect (PANAS-N) scores at baseline (Base) and after the task (Post) for both the FC and the CC. A significant increase in negative affect was observed only in the FC following the frustration task, while no significant change was found in the CC. **B** Task-related confidence levels. Participants in the FC reported significantly lower confidence in their task performance compared to those in the CC group. These behavioral results provide a manipulation check, confirming that the frustration task successfully induced a negative emotional state and reduced subjective performance confidence in the FC. **p < 0.01, NS = not significant. FC Frustration Condition, CC Control Condition.

Paired-sample t-tests were employed to examine whether the frustration task effectively elicited negative affect. The results revealed a significant increase in negative affect measures during the frustration task compared to baseline levels (Baseline: *M* = 17.62, *SD* = 3.85; Frustration: *M* = 20.80, *SD* = 4.75; *t*_(34)_ = -2.89, *p* = 0.007, *Cohen’s d* = 0.48; Fig. 1A). Conversely, no significant differences were observed in the CC’s negative affect measures (*p* > 0.05). Additionally, independent samples t-tests were conducted to investigate differential emotional responses post-frustration across conditions. The results indicated that participants in the FC exhibited significantly higher negative affect scores compared to the CC (*t*_(68)_ = 3.34, *p* < 0.001, *Cohen’s d* = 0.79; Fig. 1A). To ascertain the absence of baseline differences in negative affect, an independent samples t-test was administered, confirming no significant intergroup variance at baseline (*t*_*(68)*_ = -0.75, *p* = 0.46). Regarding confidence levels, independent samples t-tests were used to explore variations between conditions. The results showed that the FC reported significantly lower confidence (*M* = 11.43, *SD* = 2.03) than the CC (*M* = 13.18, *SD* = 1.45; *t*_*(*68*)*_ = −4.15, *p* < 0.001, *Cohen’s d* = 0.90; Fig. 1B). These outcomes validate the efficacy of our frustration task in inducing a state of frustration and its associated emotional and psychological consequences in participants.

### Behavioral performance

**Fig. 2:**
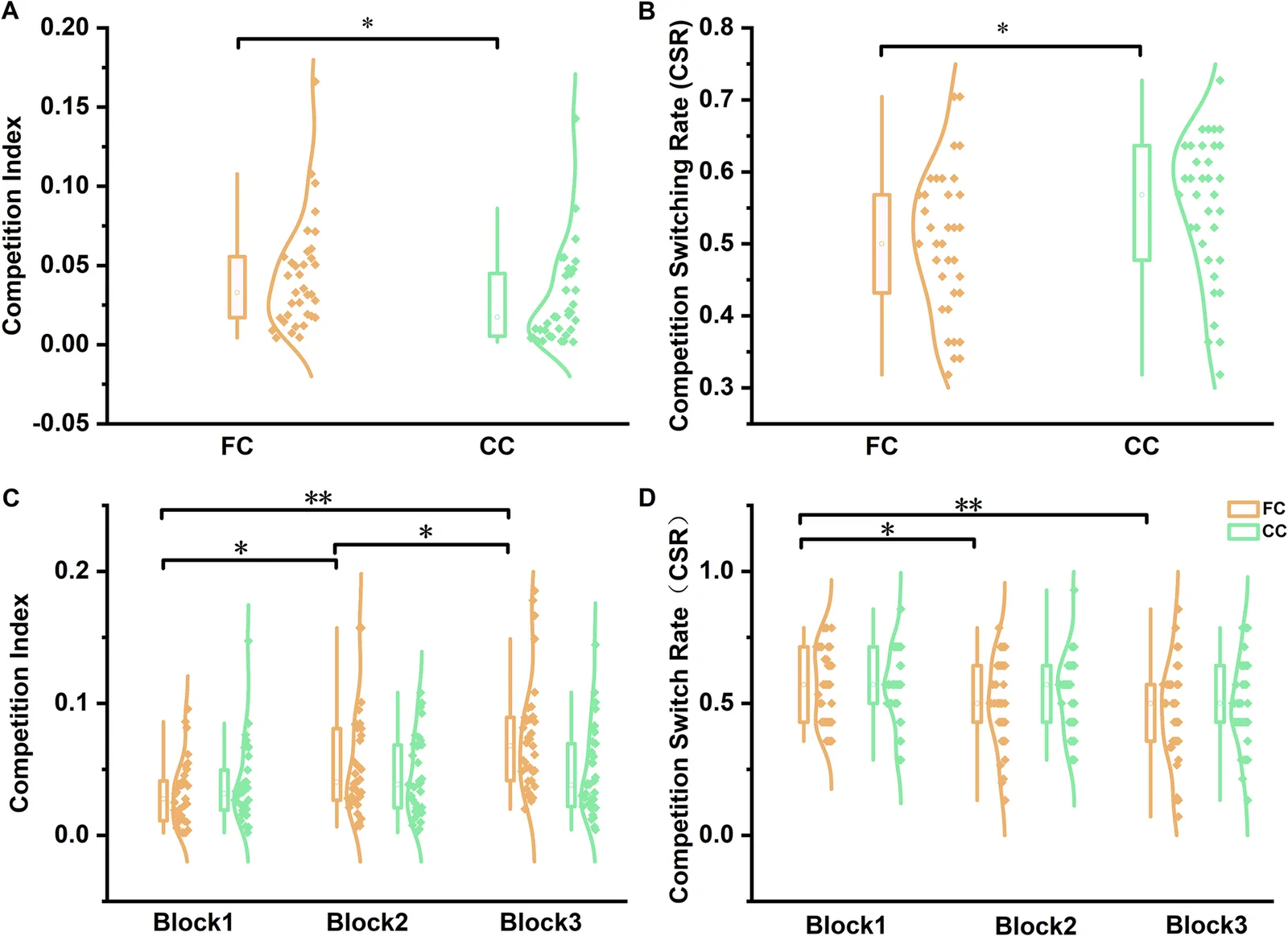
Behavioral performance and dynamic progression during the competitive task (*n* = 35 dyads). **A** Competition index. Independent-samples t-tests showed that the FC exhibited a significantly higher competition index than the CC, indicating reduced competitive willingness following frustration induction. **B** Competition Switch Rate (CSR). The FC showed a significantly reduced CSR compared to the CC. **C** Dynamic changes in competition index across blocks. A two-way ANOVA revealed a significant Condition × Block interaction. In the FC, the competition index increased significantly from Block 1 to Block 3, whereas no such temporal change was observed in the CC. **D** Dynamic changes in CSR across blocks. A significant main effect of Block was found, with CSR gradually decreasing from Block 1 to Block 3 across both conditions, reflecting a general stabilization of strategy over time. These findings indicate that frustration does not exert a static impact but instead attenuates interpersonal competitive interactions dynamically. * *p* < 0.05, ** *p* < 0.01.

Independent samples t-tests were utilized to examine the differences between the two behavioral metrics across conditions. Results revealed that the FC (*M* = 0.042, *SD* = 0.034) exhibited a higher competition index on the competition task compared to the CC (*M* = 0.027, *SD* = 0.020), *t*_(68)_ = 2.03, *p* = 0.047; Fig. 2A. Furthermore, the FC (*M* = 0.50, *SD* = 0.10) exhibited a reduced CSR on the competition task compared to the CC (*M* = 0.55, *SD* = 0.09), *t*_(68)_ = −2.01, *p* = 0.043; Fig. 2B. These findings suggest that dyads who experience frustration may exhibit a diminished willingness to compete during the competition task compared to the CC. Notably, although both behavioral indices showed significant between-condition differences, the effect sizes were relatively modest, suggesting that the influence of frustration on competitive behavior may be subtle and warrants further investigation.

To further examine the fluctuations in dynamic competitive behavioral performance elicited by frustration experiences across blocks, a two-way analysis of variance (ANOVA) was performed with Condition (FC, CC) and Block (Block1, Block2, Block3) as independent variables and the competition index and CSR of the blocks as dependent variables. Results revealed that the interaction between Condition and Block was statistically significant (*F*_(2, 136)_ = 4.96, FDR-corrected *p* < 0.05, *η*_*p*_^2^ = 0.06; Fig. 2C). Subsequent simple effect analysis indicated that the competition index in the FC of Block3 (*M* = 0.068, *SD* = 0.042) was significantly higher than that in Block1 (*M* = 0.028, *SD* = 0.024), *p* = 0.002. Block2 (*M* = 0.040, *SD* = 0.037) was significantly higher than that in Block1. Block3 exhibited a significantly higher competition index than Block2. However, no significant difference was observed in the CC (*p*s > 0.05).

Regarding the CSR, the main effect of Blocks was found to be significant (*F*_(2, 136)_ = 5.72, FDR-corrected *p* < 0.05, *η*_*p*_^2^ = 0.078; Fig. 2D), with post-hoc comparisons indicating that the CSR of Block2 was significantly lower than that of Block1, and that the CSR of Block3 was significantly lower than that of Block1. CSR decreased gradually across blocks. The main effect of Condition and the interaction between Condition and Block were not significant (*p*s > 0.05). These findings suggest that frustration may attenuate interpersonal competitive interactions over time.

### Increased task-related INS

**Fig. 3:**
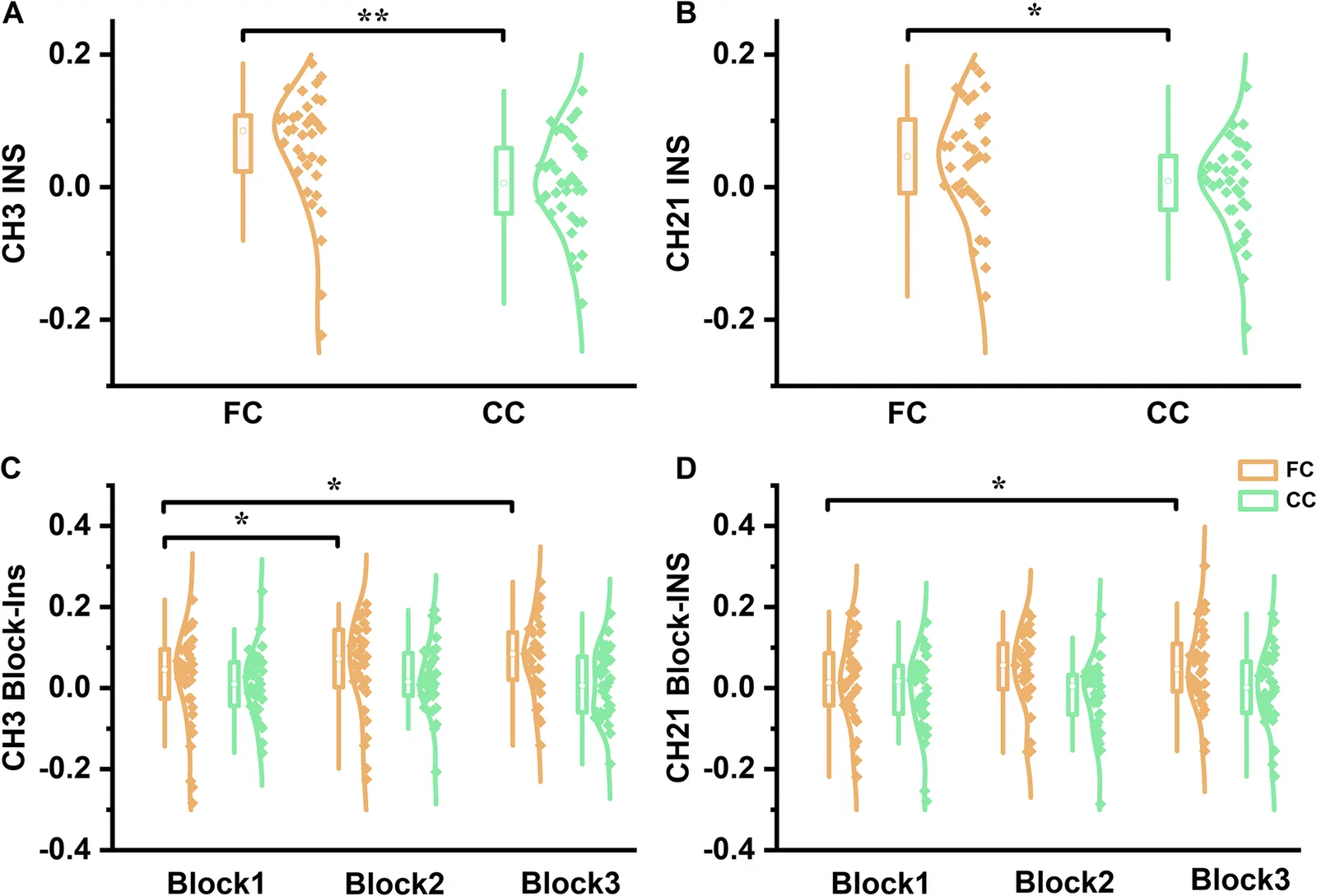
INS and dynamic progression during the competitive task (*n* = 35 dyads). **A** INS in CH3. Independent samples t-tests showed that FC exhibited significantly higher INS in CH3 compared to CC. **B** INS in CH21. Independent samples t-tests showed that FC exhibited significantly higher INS in CH21 compared to CC. **C** Dynamic changes in CH3 INS across blocks. Paired-samples t-tests within FC revealed that INS in CH3 increased significantly from Block 1 to the subsequent blocks. In contrast, no such temporal increase was observed in CC. **D** Dynamic changes in CH21 INS across blocks. In FC, INS in CH21 showed a significant increase in the Block 3 compared to Block 1, whereas INS in CC remained stable across the entire task. These findings demonstrate that frustration significantly enhances neural synchronization in both the FPC and dlPFC during competition. INS is not static but evolves dynamically, showing a progressive upward trend in frustrated dyads as the interaction unfolds. * *p* < 0.05, ** *p* < 0.01.

Based on a series of one-sample t-tests, task-related INS was significantly increased in two channels in frontal regions during the competition task, with a significant increase observed in CH3 and CH21 in the FC, which mainly overlapped with the FPC and dlPFC (CH3: *t*_(34)_ = 4.058, CH21: *t*_(34)_ = 2.804, *p* < 0.05, FDR-corrected), while no significant channels were found in the CC. An independent samples t-test was performed to examine between-condition differences using CH3 and CH21 as the channels of interest. The results indicated that in the FC (*M* = 0.060, *SD* = 0.088), the INS was significantly higher in CH3 than in the CC (*M* = 0.007, *SD* = 0.072), *t*_(68)_ = 2.749, *p* = 0.008, Cohen’s *d* = 0.657; Fig. 3A. Similarly, in the FC (*M* = 0.040, *SD* = 0.085), the INS of CH21 was significantly higher than in the CC (*M* = 0.001, *SD* = 0.071), *t*_(68)_ = 2.153, *p* = 0.034; Cohen’s *d* = 0.491; Fig. 3B.

Furthermore, we examined how task-related INS varied across blocks for the dyads in both conditions. A two-way ANOVA was performed on the effects of Condition (FC, CC) and Block (Block 1, Block 2, Block 3) on task-related INS. The main effect of Condition was significant, *F*_(1, 68)_ = 4.442, *p* = 0.039, *η*_*p*_^2^ = 0.068, and further post hoc comparisons indicated that the INS for FC (*M* = 0.050, *SD* = 0.012) was significantly larger than for CC (*M* = 0.015, *SD* = 0.012). The main effect of Block, and the Condition × Block interaction was not significant. When differentiated by Block, paired-samples t-tests revealed that in FC, Block 2 (*M* = 0.054, *SD* = 0.109) exhibited a higher INS than Block 1 (*M* = 0.021, *SD* = 0.116), *t*_(34)_ = -2.256, *p* = 0.031. Block 3 (*M* = 0.076, *SD* = 0.090) also demonstrated a higher INS than Block 1, *t*_(34)_ = -2.316, *p* = 0.027. The difference between Block 2 and Block 3 was not significant. In CC, differences between Blocks were not significant, *p* > 0.05, Fig. 3C.

In CH21, the main effect of Condition was significant, *F*_(1, 68)_ = 6.216, *p* = 0.015, *η*_*p*_^2^ = 0.084, and further post-hoc comparisons revealed that the INS for FC (*M* = 0.041, *SD* = 0.014*)* was significantly higher than in the CC (*M* = −0.008, *SD* = 0.092). If differentiated by Block, paired-samples t-test revealed that in FC, Block 3 (*M* = 0.061, *SD* = 0.090) exhibited a higher INS than Block 1 (*M* = 0.027, *SD* = 0.098), *t*_*(34)*_ = −2.316, *p* = 0.027. The remaining comparisons were not significant; the main effect of Block and the Condition × Block interaction were also not significant, *p* > 0.05, Fig. 3D.

### Validation analysis of task-related INS

**Fig. 4:**
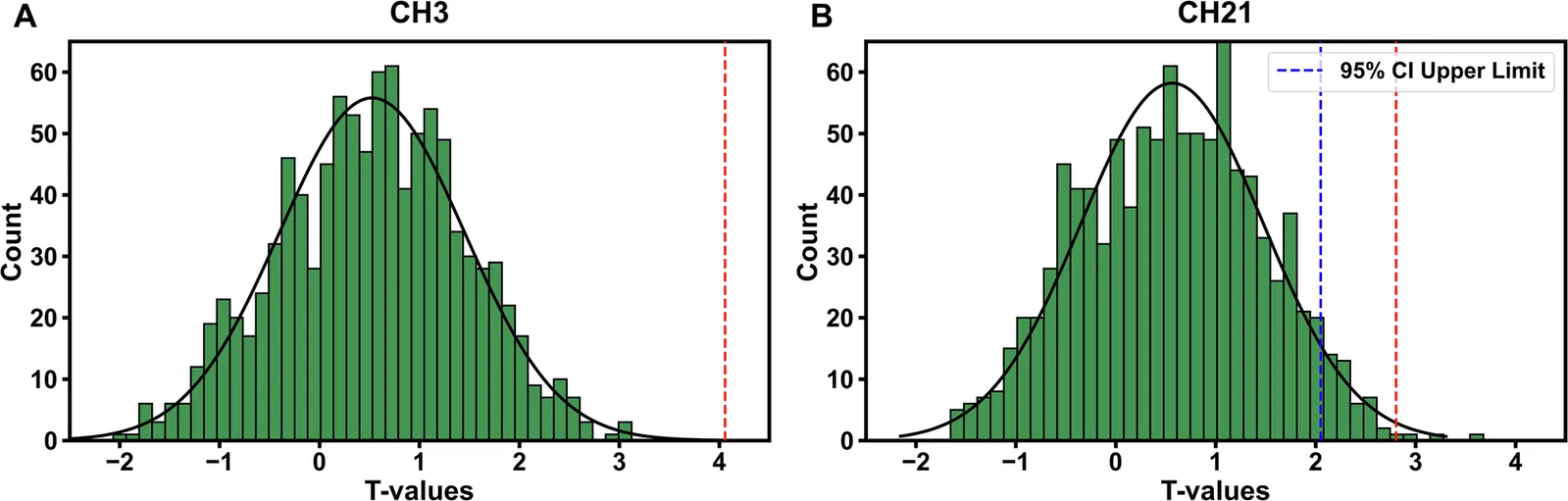
Validation of task-related INS via permutation tests. **A** Permutation results for CH3. The histogram illustrates the null distribution of t-values generated from 1,000 random dyadic permutations. The observed t-value from the actual dyads (indicated by the red dashed line) fell within the upper 5% tail of the distribution (*p* < 0.001), confirming that the INS in CH3 is specific to real interacting dyads. **B** Permutation results for CH21 (dlPFC). The observed t-value for CH21 (red dashed line) is significantly higher than the *t*-values derived from 1,000 random permutations (*p* < 0.01), falling beyond the 95% confidence interval upper limit (blue dashed line). Red dashed lines represent the observed t-values from real dyads. The blue dashed line represents the 95% confidence interval upper limit of the null distribution.

After permutation randomization, the validation results showed (t-values) for 1000 permutations of CH3 and CH21. The t-values of the actual dyads fell within the upper 5% of the permutation-generated distribution, indicating that the observed task-related INS was specific to the original dyadic pairings. These findings demonstrated that the significantly increased task-related INS was specific to the original dyads, Fig. 4A, B.

### Correlation and mediation analysis

**Fig. 5:**
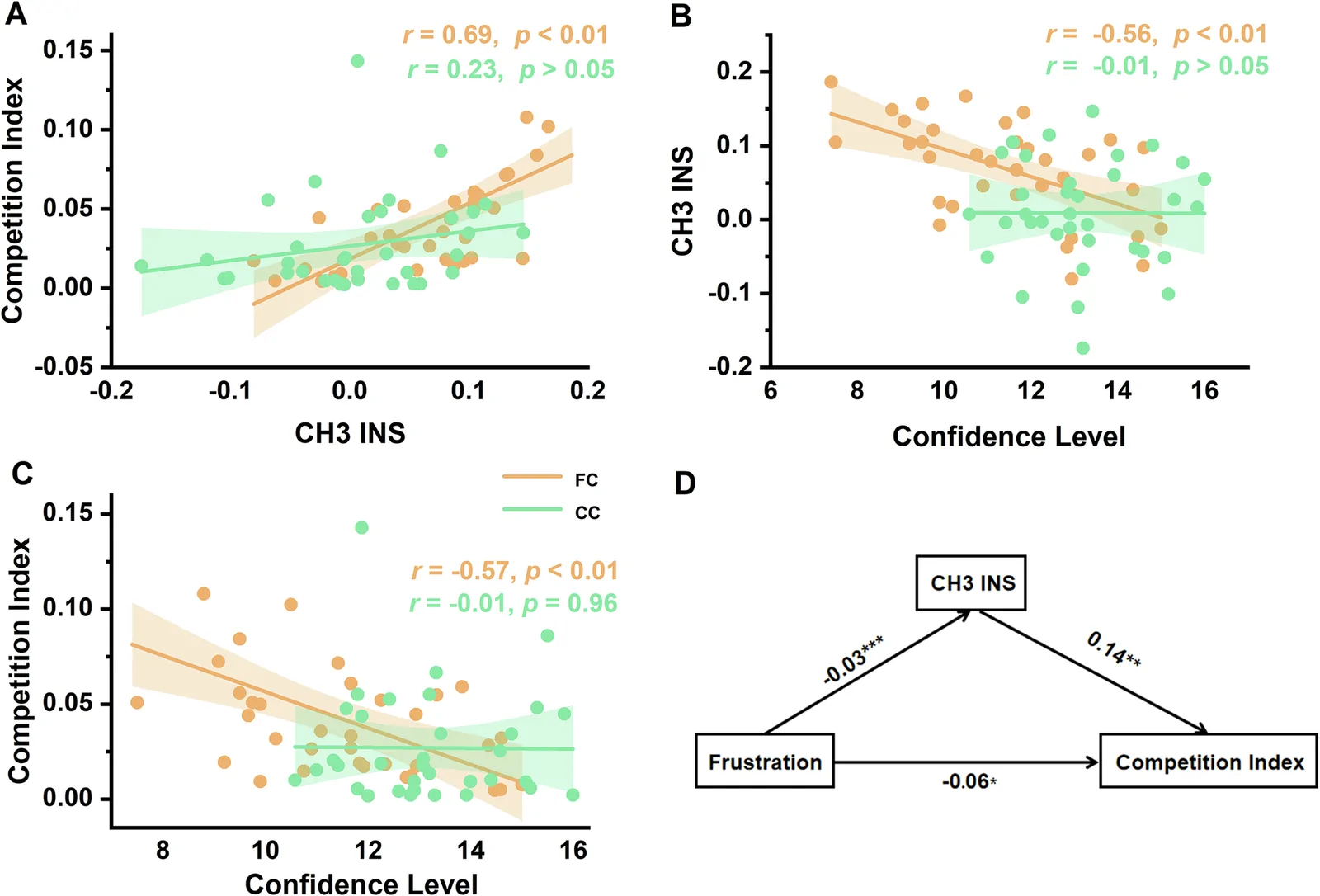
Relationships between INS, psychological states, and competitive behavior (*n* = 35 dyads). **A** Correlation between CH3 INS and competition index. Scatter plots show a significant positive correlation between task-related INS in CH3 and the competition index for FC, while no significant relationship was found for CC. **B** Correlation between CH3 INS and confidence level. In FC, higher INS in CH3 was significantly associated with lower subjective confidence levels. **C** Correlation between competition index and confidence level. A significant negative correlation was observed between the competition index and confidence levels in FC. **D** Mediation model. Path analysis reveals that the INS in CH3 significantly mediates the relationship between the frustration and the subsequent competition index. Standardized coefficients are shown on the paths, with asterisks indicating levels of statistical significance. *p < 0.05, ***p < 0.001. Shaded areas represent the 95% confidence interval.

Pearson correlation analyses were conducted on the task-related INS and the competition index. Positive correlation between task-related INS of CH3 and the competition index was observed in the FC (*r* = 0.69, *p* < 0.01), while no correlation was found at CC (*r* = 0.10, *p* = 0.60; Fig. 5A). Task-related INS of CH21 showed no significant correlation with the competition index in either condition (*p*s > 0.05). Regarding CSR, Pearson correlation analyses revealed a significant positive correlation between task-related INS and CSR in FC for CH21 (*r* = 0.43, *p* = 0.021), but not in CC (*p* > 0.05). Task-related INS in CH3 showed no correlation with CSR in either condition (*p*s > 0.05). These findings suggest that task-related INS plays a crucial role in interpersonal competition. Additionally, we explored the relationship between indicators of frustration experience and task-related INS. Pearson correlation analyses revealed significant negative correlations between confidence level and task-related INS of CH3 in the FC (*r* = −0.56, *p* < 0.01), but not in the CC (*p* > 0.05; Fig. 5B). In CH21, no significant correlations were found between negative affect, confidence levels, and task-related INS in either condition (*p*s > 0.05). Furthermore, we investigated the impact of frustration experiences on interpersonal competitive behavior, conducting Pearson correlation analyses between frustration experiences (confidence level) and indicators of interpersonal competition (competition index and CSR). A significant negative correlation was observed between confidence level and the competition index under FC conditions (*r* = −0.57, *p* < 0.001, Fig. 5C), whereas no such correlation was found under CC conditions (*p* > 0.05). No significant correlations were found between negative affect and the competition index, between negative affect and CSR, or between confidence levels and CSR in either condition (*p*s > 0.05).

Synthesizing these findings, under FC conditions, (i) frustration experience (confidence level) exhibited a negative correlation with INS of CH3 situated in FPC (Fig. 5B); (ii) INS of CH3 in FPC demonstrated a positive correlation with the competition index (Fig. 5A); and (iii) confidence level showed a negative correlation with the competition index (Fig. 5C). Based on these findings, we further tested whether INS in CH3, located in the FPC, mediated the relationship between frustration experience and the competition index. To address this, mediation analyses were performed employing Model 4 in SPSS PROCESS (Hayes, 2017). The mediating effects were assessed using PROCESS for SPSS (Model 4, employing 5000 bootstrap samples to generate 95% confidence intervals (CIs)), an approach that avoids assumptions regarding the normal distribution of indirect effects (Preacher & Hayes, 2008). If the 95% CI of the estimate does not include zero, the mediation effect is considered statistically significant at the 0.05 level. The analysis revealed that the indirect effect of frustration experience on interpersonal competition through the INS of CH3 was statistically significant (bootstrap ab, 95% CI [0.0447, 0.9759]). This suggests that as the confidence levels of participant dyads experiencing frustration decrease, the INS increases, subsequently reducing their inclination to compete, Fig. 5D.

### Trial-cumulative INS dynamics analysis

**Fig. 6:**
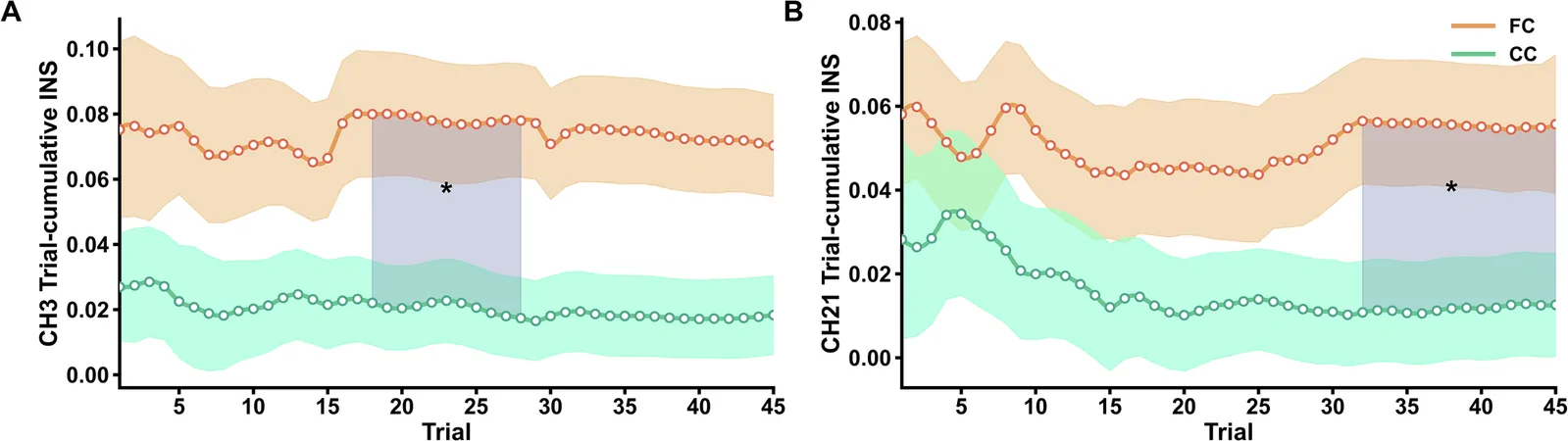
Trial-cumulative INS dynamics analysis. **A** Cumulative INS in CH3. This plot illustrates the continuous accumulation of INS across 45 trials. A stable and significant difference between FC and CC was observed between the 16th and 29th trials (indicated by the gray shaded rectangle). **B** Cumulative INS in CH21. Significant differences in CH21 emerged later in the task, starting from the 32nd trial and remaining consistent thereafter. Notes: Shaded bands around the solid lines represent the standard error of the mean (SEM). Gray shaded rectangles with asterisks (*) indicate stable significant windows (*p* < 0.05).

To comprehensively explore the dynamics of dyads’ INS in CH3 and CH21 as time progressed during interpersonal competition, we computed the trial-cumulative INS. A stable and significant difference in the cumulative INS of CH3 between FC and CC was observed between the 16th-29th trials, *p* < 0.05, FDR corrected, Fig. 6A. Starting from the 32nd trial, CH21 exhibited consistent between-condition differences, *p* < 0.05, FDR corrected, Fig. 6B.

### Coupling directionality

In FC, G-causalities were significantly greater than zero in both directions: from leader to follower (*t*_(34)_ = 10.29, *p* < 0.001) and from follower to leader (*t*_(34)_ = 17.80, *p* < 0.001). Results in CC were similar to FC, with significant increases in G-causalities relative to zero in both directions: from leader to follower (*t*_(34)_ = 8.88, *p* < 0.001) and from follower to leader (*t*_(34)_ = 12.26, *p* < 0.001).

**Fig. 7:**
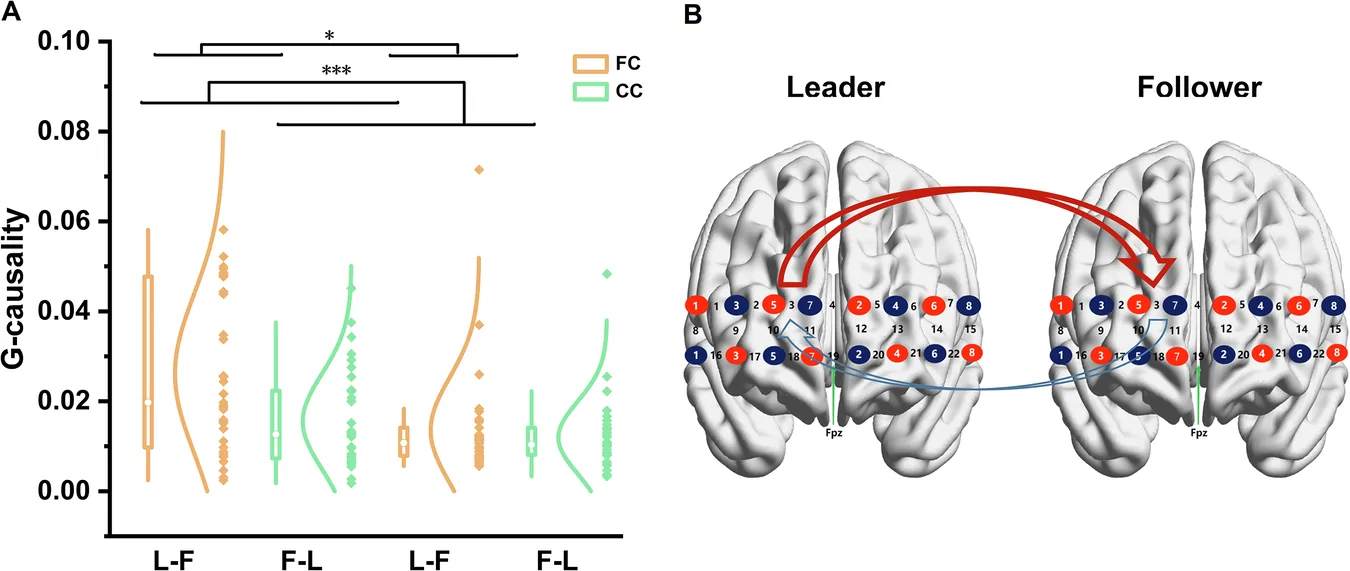
Granger causality (G-causality) and coupling directionality (*n* = 35 dyads). **A** Comparison of G-causality values. In FC, the G-causality from leader to follower (L-F) was significantly greater than from follower to leader (F-L), whereas no such directional difference was observed in CC. **B** Visualization of effective connectivity. The brain diagrams illustrate the direction and strength of information flow between the leader and the follower. The thicker red arrow represents the dominant influence from the leader to the follower in FC. *p < 0.05, ***p < 0.001. L-F = leader to follower; F-L = follower to leader.

A 2 (Condition: FC, CC) × 2 (Direction: leader-to-follower, follower-to-leader) two-way ANOVA was conducted in CH3. The results showed a significant main effect of Condition (*F*_(1, 68)_ = 8.40, *p* < 0.05, *η*_*p*_^2^ = 0.11) and greater G-causalities in FC (*M* = 0.015, *SD* = 0.015) compared to CC (*M* = 0.010, *SD* = 0.009). There was a significant main effect of Direction (*F*_(1, 68)_ = 14.60, *p* < 0.001, *η*_*p*_^2^ = 0.17) and greater G-causalities in L-F (*M* = 0.013, *SD* = 0.005) compared to F-L (*M* = 0.010, *SD* = 0.009). There was a significant interaction between Condition and Direction observed (*F*_(1, 68)_ = 4.51, *p* = 0.037, *η*_*p*_^2^ = 0.06). Further simple effects analyses found that leader-to-follower G-causalities were significantly larger than follower-to-leader G-causalities in FC (L-F: *M* = 0.019, *SD* = 0.017; F-L: *M* = 0.011, *SD* = 0.010, *p* < 0.05, Fig. 7A, B). In CC, the difference was not significant.

## Discussion

In the present study, we investigated the dynamic behavioral and neural synchronization mechanisms of interpersonal competitive response patterns following frustration experiences using fNIRS-based hyperscanning. Our findings confirmed that compared to the CC, participants who underwent frustration showed a reduction in interpersonal competitive willingness across task blocks, accompanied by a range of dynamic inter-brain coupling activities in the PFC. Specifically, throughout the interpersonal competition task, task-related INS in the FPC appeared at a middle stage, whereas task-related INS in the dlPFC appeared at a late stage. Further trial-cumulative INS results revealed stable and significant between-condition differences in the FPC during the 16th–29th trials, and significant differences in the dlPFC from the 32nd trial onward. We also found that INS in the FPC was significantly and positively correlated with the competition index and mediated the effect of frustration on competition performance. In addition, we revealed a greater flow of directional information from leader to follower when the roles of the participants were asymmetrical during the interpersonal competition task.

### INS characteristics during interpersonal competition

Some studies have suggested that INS may not be temporally static during measurement^21^. Furthermore, interpersonal competition is inherently a highly dynamic process of social interaction, involving complex cognitive and strategic adjustments in real time^28^. Therefore, we investigated the INS dynamics of the interpersonal competition task.

The reduction in competitive willingness in the second block was accompanied by an increased task-related INS in FPC, and further dynamic INS analyses revealed a stable and significant between-condition difference in trial-cumulative INS in CH3 observed in the 16th-29th trials. FPC has been found to play an important role in cognitive control^40,41^ and executive function^2,42^. Thus, in our study, the increase in INS in the second block may reflect cognitive control over competitive behavior, such as adjusting one’s key-press behavior in response to shared frustration, the opponent’s historical reaction-time distribution, and performance changes across consecutive trials, thereby mitigating interpersonal competition. This was supported by our results, where CSR was significantly lower in Block2 compared to Block1, suggesting that the intense competition between two individuals who were continuously competing against each other was mitigated.

As the task progressed to the third block, task-related INS appeared in the dlPFC, and further dynamic INS analyses revealed that significant between-condition differences in trial-cumulative INS in CH21 were observed for the 32nd trial. dlPFC is known to be associated with integrating information about oneself and others^43,44^, inferring others’ intentions^45^, and theory of mind^46^. It is also involved in the shaping and control of behavior during actions^47,48^. As mentioned earlier, interpersonal competition tasks involve interactive communication in the form of simultaneous key presses, and the feedback at the end of each trial not only affects the individual’s sense of self-efficacy and motivation level^49^, but also provides indirect information about the opponent’s performance, prompting participants to continually revise their assessment of the opponent’s abilities and strategies. This dynamic adjustment process not only demonstrates the flexibility of self-perceived control^16^, but also facilitates dynamic updating of others’ intentions^44,46^. Furthermore, our findings align with previous research demonstrating that task-related INS in the dlPFC increases during interpersonal competition when dyads engage in mutual prediction^50^. Our study thus further supports the ‘competitive mutual prediction’ hypothesis, whereby predicting others’ mental states and adapting to changes in the current situation subsequently enhances favorable decision-making strategies^50,51^.

In summary, we propose that this process reflects a dynamic evolution from executive resource regulation to intentional alignment. During the middle stage of the task, the enhanced synchronization in the FPC reflects interpersonal co-regulatory control directed at frustration. This regulatory process possesses distinct high-load cognitive attributes. When the brain allocates substantial resources to the FPC for monitoring and suppressing negative emotions triggered by frustration, the cognitive resources originally intended to drive aggressive competitive responses are reallocated, leading to the decline in early competitive willingness^13,14^. As the task persists, the shared experience of frustration facilitates emotional bonding between individuals, which in turn promotes intentional alignment mediated by the dlPFC. Social psychological research indicates that shared negative experiences, particularly in frustrating contexts, can enhance interpersonal cohesion^52^. At this stage, the INS in the dlPFC serves as a neural marker for dyads to mutually understand the intention to mitigate competition. The reduction in competition no longer relies solely on deliberate cognitive regulation but is transformed into an adaptive social behavior that fosters cohesion. This dynamic process provides a neurocognitive mechanistic explanation for understanding how INS drives the mitigation of competitive behavior.

### Relationship between INS and behavior

Exploring the potential correlation between behavioral performance and INS, our results found that confidence levels in the FC were related to the competition index. As previously discussed, interpersonal competition in the keypress task triggered synchronized mental processes (competing predictions of each other) and self-control processes (speed of the next keypress response). Based on these features, the observation that the competition index was higher in the FC than in the CC may suggest that the frustration experience of the participant dyads was more conducive to their specific actions to mitigate the willingness to compete. This could be explained by a previous study that showed a higher need to belong between two participants who experienced social exclusion together^25,53^. In our study, although participants in the pre-experimental dyad did not know each other, the shared experience of frustration gave them strong emotional resonance and may have driven both parties to engage in deep self-regulation through a strong motivation for self-regulatory action, which resulted in a higher competition index found in the interpersonal competition task^26,54^. In addition, our results observed that the positive correlation between competition-related INS and the competition index in FPC was confirmed in the FC but not the CC. This suggests that the higher the competition index, the greater the task-related INS in the FPC. This finding echoes recent neuroimaging studies that mutual prediction in a competition task promotes interbrain synchronization^50^. Interestingly, task-related INS in the FPC mediated the relationship between confidence levels during frustration and the competition index. Previous research has shown that greater INS during mutual prediction is strongly associated with behavioral performance in the rock-paper-scissors game^50^. Consistent with these findings, our results further support the mentalization process of mutual prediction. On the other hand, previous studies have shown that emotions are associated with INS if individuals are exposed to similar emotional content^18,19,55^. In addition, previous related studies have suggested that positive emotional engagement may lead to an increase in INS compared to neutral emotional states^55^. A recent fNIRS-based hyperscanning study found that emotional connection between parents and their children was associated with INS in their cooperation^56^. Our study extends previous findings by showing that lower levels of confidence in frustration experiences in participant dyads were associated with higher interpersonal competition indices, and that this effect was mediated by task-related INS between interactors.

### Role asymmetry in interpersonal competition

Leadership is a universal feature of social species^31^. Leader-follower relationships emerge spontaneously among participants in leaderless group discussions, even though they have no differences in natural or social status^31,57^. However, previous studies on interpersonal synchronization of interpersonal competition have not taken into account role asymmetry. In this study, two participants of the same gender completed a frustration task together, and based on their interaction performance, one member was identified as a leader and the other as a follower. Research on the generation of leadership has found that when a group encounters frustration and is faced with decisions, members who make the final decisions and take responsibility for the results tend to become leaders more often^32,33^. GCA was originally used to track the direction of information flow between different areas within a brain, and recently has been expanded to track the direction of information flow across interlocutors’ brains during social interactions^34^. The G-causalities could be used as an indicator of the influence exerted by one individual on another individual at the brain level^22^. Many lines of evidence have revealed significant directional differences in INS within interactive dyads^34,58^. For example, primary information flow in brain coherence has been observed from the gesturer to the guesser in a charades game^59^, from the instructor to the learner in a social interactive learning task^60^ and from the banker to the follower in a gambling card-game^61^. Our results showed that the influence between individuals in each dyad was bidirectional. However, the influence from the leaders on the followers was significantly stronger than the other direction. That is, although the leaders and followers were mutually influenced, the primary information flow was from leaders to followers. Specifically, the past neural activity of the leaders can better predict the future neural activity of the followers. These results may suggest that although both participants were actively engaged in the interaction, the leaders may entrain the followers’ neural activity more during the interpersonal competition interaction^60^.

### Practical implications for real-world interpersonal competition

The present study provides empirical insights into the management of interpersonal competition in real-world environments. First, the finding that shared frustration suppresses competitive impulses by enhancing INS in the FPC suggests that constructing a cognitive framework of shared adversity in deadlocked situations can effectively activate behavioral regulation, thereby mitigating irrational interpersonal competition^25^. Second, the evolution of competition mitigation from early-stage behavioral inhibition (FPC-based INS) to late-stage intention inference (dlPFC-based INS) indicates that conflict intervention strategies should be staged. In the initial phase of competition, priority should be given to reinforcing self-control through rule-based constraints. In contrast, during sustained interaction, the focus should shift toward enhancing informational transparency to facilitate perspective-taking, enabling a transition from maladaptive rivalry to strategic coordination^50,51^. Finally, our GCA results demonstrate that the directional influence of leaders is amplified within frustrating competitive environments. This underscores the critical importance of training leaders to maintain composed and rational social signals, which can more effectively synchronize the decision-making logic of the entire team and prevent collective lapses into irrational competition^31,60^.

### Limitations

The limitations of the current study should also be addressed. First, our fNIRS optical probe set covered only the prefrontal cortex of the brain, leaving other regions unexplored. Previous studies have found that the temporoparietal regions also cause increased INS during competitive tasks^62,63^. Future research could further investigate the role of other brain regions in competitive tasks using whole-brain measurements. Secondly, according to Self-Determination Theory, individuals suffering from prolonged frustration are more inclined to take measures to compensate, such as finding substitutes for needs, relaxing self-control, and developing undesirable behaviors^12^. It has also been found that persistent frustration reduces an individual’s level of motivation to engage in subsequent tasks^64^. Future research could further compare behavioral and neural differences between short-term and long-term frustration in competitive tasks. Finally, the current study did not fully account for individual differences within the dyads. Individual traits, such as frustration tolerance and competitive orientation, may significantly modulate an individual’s perception of setbacks and subsequent patterns of interpersonal competition^5,28^. Future research should specifically investigate the moderating effects of these individual characteristics, integrating other psychosocial factors—such as social value orientation^65^ and psychological resilience^66^—into a comprehensive analysis. Such efforts would provide a more nuanced understanding of the inter-individual variability in co-experienced frustration and competitive behavior.

## Conclusion

Within the framework of two-person neuroscience, we used fNIRS-based hyperscanning to integrate relevant parameters at the behavioral and brain levels to better understand the dynamic effects of frustration on interpersonal competition. We found that when dyads performed an interpersonal competition task following a frustration experience, task-related INS in the FPC and dlPFC emerged successively, accompanied by a gradual decrease in willingness to compete. This suggests that frustration dynamically reduces interpersonal competition, potentially through changes in INS. We also found that INS in the FPC was significantly and positively correlated with the competition index and mediated the effect of frustration on competition performance. We also identified roles in interpersonal competition and found that leaders exerted greater influence on followers. This suggests that role asymmetry within dyads plays a critical role in interpersonal competition, particularly in the context of frustration. These results provide neural evidence for competitive interactions in the context of frustration and contribute to a better understanding of the dynamic mitigating effects of frustration experiences on competition. Looking ahead, several key directions merit further investigation. For instance, the impact of the heterogeneity of frustration types on interpersonal competition warrants deeper exploration. Furthermore, subsequent studies should strive to validate these findings in more ecologically valid real-world settings.

## Methods

### Participants

For this study, 146 college students with normal or corrected-to-normal vision and no reported physical or psychological health problems were recruited. All participants were right-handed individuals. Every participant was randomly matched with an unfamiliar counterpart. Each pair, composed of two individuals of the same gender, was randomly allocated to either the FC or CC. Three dyads were excluded from the analyses because of failure to establish relative leader–follower role differentiation, prolonged task response times, or unsuccessful fNIRS data acquisition. Consequently, the ultimate dataset comprised 35 dyads in FC (mean age = 19.45 ± 1.35 years, females = 40) and 35 dyads in CC (mean age = 19.60 ± 1.55 years, females = 36). Written informed consent was obtained from all participants prior to the experiment. The study protocol was approved by the Medical Ethics Committee of North China University of Science and Technology (No. 2023030). All ethical regulations relevant to human research participants were followed. Participants received monetary compensation, with additional performance-based incentives.

### Experiment tasks and procedures

**Fig. 8:**
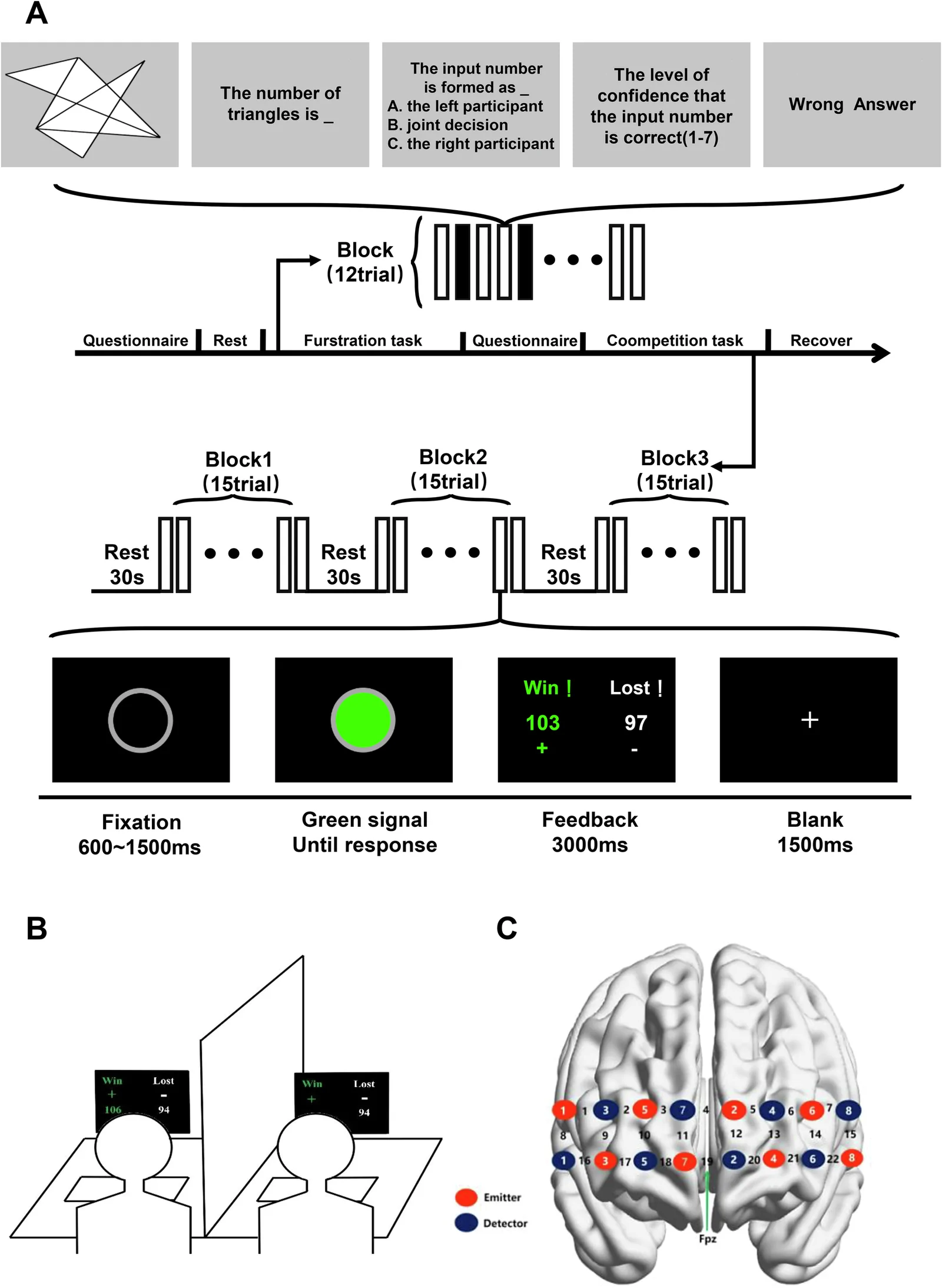
Experimental protocol. **A** This figure shows the experimental timeline, including 12 trials of the frustration task and three blocks (15 trials each) of the competition task. The procedure for each trial of both tasks is described in detail. **B** Two participants sat on either side of a table, each with a monitor and keyboard. **C** The fNIRS optode probe set was placed on the prefrontal cortex. The numbers indicate measurement channels (CHs). The patch was placed horizontally, following the international 10–20 system. All optode probe sets were positioned using individualized caps made from swimming caps, ensuring signal consistency across variations in head size.

The experimental schedule is depicted in Fig. 8A. Two participants arrived at the laboratory and were ushered into a secluded room after confirming their unfamiliarity with each other. Subsequently, the two participants were positioned side-by-side at an elongated table equipped with a computer screen and two keyboards placed before them, while a partition board was inserted in the center to foster a lifelike interactive setting (Fig. 8B). Upon seating, the experimenter meticulously elucidated the entire experimental procedure to ensure comprehensive comprehension of the experimental requirements. The experimental protocol comprised several stages, encompassing two questionnaire administration sessions (pre-frustration task: demographic questionnaire, state-check questionnaire, and Positive and Negative Affect Schedule (PANAS); post-frustration task: PANAS) and two task modules (frustration-inducing task or control task; interpersonal competition task), culminating in a concluding recovery phase.

### Frustration induction task

The frustration-inducing task was adapted from previous research^7^. To ensure the scientific validity of the task duration and difficulty, an independent sample of 24 dyads was recruited to pre-evaluate 20 candidate geometric images using an 8-point Likert scale (1 = particularly easy, 8 = especially difficult). Based on their evaluations, 10 medium-difficulty images (range: 3.55–5.13) and 2 low-difficulty images (range: 1.46–2.34) were ultimately selected as stimulus materials for the formal task, which consisted of 12 trials. In each trial, complex geometric images were presented for a maximum duration of 120 seconds. Participants were permitted oral communication while remaining physically still to reach a consensus on the number of triangles. If a consensus was reached before the time limit, participants could press the spacebar to terminate the image presentation and proceed immediately to the answer entry phase. Following the image presentation, the participant seated on the right entered the agreed-upon answer. Subsequently, to distinguish between roles within the dyad, participants were prompted to identify the decision-making process: (i) dominance by the left-side participant (2 points), (ii) joint determination (1 point each), or (iii) dominance by the right-side participant (2 points). After 12 trials, the individual with the higher cumulative score was defined as the “leader,” and the other as the “follower.” Immediately after role evaluation, participants assessed their confidence in the accuracy of the input number using an untimed 7-point Likert scale (1 = not at all confident, 7 = extremely confident), with the left-side participant responding first. Finally, a feedback screen was presented for 3000 ms. To consistently induce frustration, feedback was manipulated as “Incorrect Answer!” (totaling 10 trials, with an 83.3% failure rate) regardless of the actual response, and “Correct Answer!” was only displayed during the 2nd and 5th trials (low-difficulty images). Finally, a blank screen was presented during the inter-trial intervals.

### Interpersonal competition task

The entire task comprised three blocks, each consisting of 15 trials^16^. To enhance clarity in measuring our experimental objectives, the fact that both participants experienced frustration together was reiterated via an instructional phrase preceding each block, whereas the control condition did not receive such reinforcement. At the onset of each trial, a hollow gray circle appeared randomly at the center of the participant’s computer screen for a duration ranging from 0.6 to 1.5 seconds. Subsequently, upon the appearance of the green signal replacing the gray circle, participants were instructed to press a key as quickly as possible. Participant A (positioned on the left) was directed to press the ‘0’ key, whereas Participant B (positioned on the right) was directed to press the ‘1’ key. Participants were instructed to respond more rapidly than their counterparts to earn a point, with points deducted for slower responses. Following the response, the feedback screen was presented for 1.5 seconds, indicating either ‘Win!’ or ‘Lost!’ along with the cumulative score for each participant.

### Recovery task

Upon completion of the interpersonal competition task, participants were instructed to take a 3-minute break. Following the break, the experimenter provided a detailed description of the frustration task to the participants and assessed their skepticism regarding the experiment’s purpose. No participant reported suspicion about the experiment’s intent or the task process.

### Questionnaire measures

Demographic questionnaire: Participants were asked to complete a brief questionnaire that included information such as gender, age, and past medical history. Furthermore, it has been shown that emotional states such as anxiety and depression can affect an individual’s willingness to compete^5,28^. To control for the influence of these potential factors on the task, the Spielberger State-Trait Anxiety Inventory-State version (STAI-S)^67^ and the Beck Depression Inventory, Second Edition (BDI-II)^68^ were used to assess the participants’ emotional state prior to the start of the experimental task. Only after scoring within the normal range were participants allowed to proceed to the follow-up task.

Emotion self-assessment measurements: In the present study, we used the PANAS to assess participants’ emotional state before and after a frustration task to evaluate the effectiveness of the task^35^. The PANAS consists of two subscales: Positive Affect (PA) and Negative Affect (NA). The PA subscale assesses positive emotions and consists of 10 positively worded items, while the NA subscale assesses negative emotions and consists of 10 negatively worded items. Additionally, to enhance the precision of frustration measurement induced by the task, we incorporated a specific ‘frustration’ item into the NA subscale, facilitating direct assessment of participant frustration levels. All items were rated on a 7-point Likert scale, ranging from 1 (not at all) to 7 (extremely). The internal consistency of the scales was assessed using Cronbach’s alpha (>0.85), and all results indicated that the internal consistency was at an acceptable level.

### fNIRS data acquisition

Two identical fNIRS systems (LIGHTNIRS; Shimadzu Co., Japan) were utilized to record the hemodynamic signals of two participants in a dyad during the experiment. This fNIRS system has a sampling rate of 13.3 Hz and three near-infrared wavelengths (780 nm, 805 nm, and 830 nm). By utilizing an external trigger system to simultaneously send marking signals to both acquisition units, we ensured millisecond-level temporal alignment at the onset of the experiment and at each trial transition. For each participant, an 8 × 2 probe patch was positioned to cover the bilateral prefrontal cortex, resulting in a total of 22 recording channels (Fig. 8C). Each patch was equipped with 8 emitters and 8 detectors. The distance between emitters and detectors was approximately 3 cm. The placement of the emitters and detectors followed the 10–20 system^69^. The lower row of optodes was positioned along the Fp1-Fpz-Fp2 line. A 3D magnetic space digitizer (FASTRAK; Polhemus, USA) was employed to assess the anatomical positions of optodes and channels for each participant, using NZ, CZ, AL, and AR as reference points^70^. The spatial anatomical locations were further analyzed using the NIRS_SPM MATLAB package^71^, whereby individual coordinates were registered to the MNI space. Anatomical labels were determined based on the Brodmann and LPBA40 atlases. Detailed MNI information for all channels is provided in the Supplementary Table 1.

### Data analysis

#### Statistics and reproducibility

All analyses were conducted using MATLAB 2020b (MathWorks Inc., Natick, MA). Significance level was set at *p* < 0.05. False-discovery-rate (FDR) correction was applied to account for multiple comparisons.

#### Validity checks in frustration induction

To systematically evaluate the effectiveness of the frustration task in eliciting negative affect, paired-samples t-tests were employed to examine changes in participants’ negative affect before and after the task. Furthermore, to ensure the reliability of the results and rule out potential baseline differences between conditions, baseline negative affect was compared between the FC and CC using an independent-samples t-test. This step was essential for establishing a homogeneous mental state foundation for both conditions at the beginning of the experiment, thereby enhancing the validity of subsequent comparisons. Finally, given that frustration experiences often coincide with diminished individual self-confidence, we additionally scrutinized potential differences in participants’ self-confidence levels during task performance across experimental conditions, employing independent samples t-tests to reaffirm the efficacy of the frustration task.

#### Behavioral performance analysis

Building upon prior research^34,55,56^, we used an analytical framework with the objective of precisely quantifying the intensity of competition and the dynamic adjustment strategies in interpersonal competition tasks. Specifically, we capture the relative differences in reaction times (RTs) between the two parties by calculating the Competition index (Competition index = |RT1 - RT2|/|RT1 + RT2|). This index controls for the sum of RTs of the two participants. A smaller Competition index represents a higher willingness to compete, symbolizing a high degree of convergence between the two parties in terms of reaction speeds, indirectly mapping enhanced competitive motivation and tight interaction tensions. Moreover, to further refine the analysis of interpersonal competition dynamics, we propose the innovative metric of Competition Switch Rate (CSR) as a measure to evaluate the strategic flexibility and depth of interaction during competition. In practice, CSR is based on the frequency of participants’ behavior in adjusting the speed of keystrokes in response to immediate feedback on the outcome of a win or a loss, by counting the number of times the outcome feedback changed (i.e., switched from “Win!” to “Lost!” or vice versa) in consecutive trials. This metric is defined as the proportion of instances of switching within the total number of competition trials, i.e., CSR = switching times / total number of switching times in competition trial.

#### fNIRS data analysis

Preprocessing: The fNIRS data underwent preprocessing through the Homer2 toolbox and a customized MATLAB-based script. Initially, raw light intensity signals were converted into optical density (OD). Then, its quality was visually assessed according to the criterion of detector saturation or insufficient optical coupling (i.e., the absence of a heartbeat frequency of ~1 Hz in the signal’s power spectrum). Any channels that failed to meet the criterion were deleted. Ultimately, 98.8% of the channels were retained for further analysis. The channels exhibiting poor signal quality were replaced with the average values of neighboring channels. If more than one-third of the channels exhibited poor quality, the dyad was excluded^72^. Second, the principal component analysis (PCA) was employed to eliminate signal artifacts resulting from spontaneous blood flow oscillations or other systematic factors^73^. A conservative variance threshold of 80% was applied to remove noise. Correlation-based signal improvement (CBSI) was utilized to mitigate head motion artifacts^16^. In line with previous studies, the data were neither filtered nor detrended for task-related INS analyses^16,55^. Finally, the optical density data for each channel were transformed into concentrations of oxyhemoglobin (HbO), deoxyhemoglobin (HbR), and total hemoglobin (HbT) using the modified Beer-Lambert law. Given that HbO signaling is more sensitive to changes in cerebral blood flow and has been extensively utilized in fNIRS hyperscanning studies, we focused on analyzing HbO signals^16,34,74^.

#### INS data analysis

Following preprocessing, the Wavelet Transform Coherence (WTC) method was applied on each channel to compute the INS of interacting individuals^75^. In contrast to correlation methods, WTC is regarded as more appropriate due to its normalization of the signal amplitude in accordance with each time window, rendering it less susceptible to motion-induced transient spikes^76^. Compared to traditional cross-correlation methods, WTC enables the decomposition of signals in both the time and frequency domains simultaneously. Channel coherence was calculated utilizing the WTC package within a bespoke MATLAB script. The WTC of HbO time series i (m) and j (m) at wavelet scale s was determined according to the following formula:

$${{\rm{WTC}}}\left(m,s\right)=\frac{{|\left\langle {s}^{-1}{W}^{{{\rm{ij}}}}\left(m,s\right)\right\rangle |}^{2}}{{|\left\langle {s}^{-1}{W}^{{{\rm{i}}}}\left(m,s\right)\right\rangle |}^{2}{|\left\langle {s}^{-1}{W}^{{{\rm{j}}}}\left(m,s\right)\right\rangle |}^{2}}$$

where *m*, *s*, and *W* denote time, wavelet scale, and the continuous wavelet transform, respectively. To identify the task-related frequency band while avoiding the issue of double-dipping^77^, we employed a Leave-One-Subject-Out Cross-Validation (LOSO-CV) method. This approach integrates frequency selection with classification modeling to ensure the independence of the analysis process (detailed procedures are provided in *Supplementary Note 1*). The results showed increased INS for frequencies ranging from 0.12 to 0.21 Hz. This frequency band excludes low-frequency fluctuations (below 0.01 Hz) and high-frequency physiological signals, such as cardiac pulsation (∼1 Hz) and respiration (∼0.3 Hz)^78,79^. In this frequency of interest, INS was computed separately for the baseline and interpersonal competition task periods for each channel, and task-related INS was calculated by subtracting baseline INS from task-period INS. Following the acquisition of the INS values for each dyad and channel, one-sample t-tests were employed to examine whether any of the channels showed a significant enhancement of INS during competition, aiming to mitigate the risk of false findings and enhance the robustness of the results. The channels of interest in this investigation comprised all those that exhibited significant INS. The task-specific channels were then subjected to an independent samples t-test analysis and a two-way analysis of variance to assess whether there existed a difference between the FC and CC.

#### Validating the INS through a permutation test

To ensure that the higher INS observed in the frustration-conditioned interpersonal competition task stemmed from the joint interaction task performed by the two-person group members, independent of other factors, we implemented a validation analysis via recruiting a trial of pairwise randomized replacements. This rationale stems from the expectation that if the higher INS was solely attributed to the processing of the task material itself, it would vanish after randomizing the participants’ pairs, as there would be no interaction between them. It is noteworthy that new dyadic pairs of participants did not have exactly the same duration in the competition task due to individual differences in reaction times, but wavelet consistency analyses require that the durations of the two time series be equal^75^. Therefore, we clipped the data for each new dyad to ensure the time series were consistent^56^. The INS analyses were subsequently re-run, and a series of independent samples t-tests were conducted on the two newly formed data sets. This replacement process was iterated 1000 times to generate the null distribution (*t*-value), and the level of significance was determined by comparing the original paired INS against the 1000 randomly paired INS (*p* < 0.05).

#### Correlation and mediation analysis

To elucidate potential mechanistic processes linking competitive behavioral performance and neural signals in the brain, we employed Pearson correlation analyses to examine the relationship between the indicators of competitive behavior (Competition index and CSR) and the task-related INS of the significant channels. Additionally, given our study’s focus on the impact of frustrating experiences on interpersonal competition, we computed Pearson correlation coefficients to assess the relationship between negative emotions, confidence levels, and competitive behavior. Lastly, we utilized PROCESS Model 4 (containing 5000 bootstrap samples) in SPSS to examine whether the effects of frustrating experiences on interpersonal competition were mediated by task-related INS^80^.

#### Dynamics of the trial-cumulative INS analysis

To achieve a more comprehensive understanding of task- related INS and explore the initial time points of increased INS under different conditions, we conducted a trial-cumulative analysis of INS, focusing on significant channels that showed task-related INS throughout the task. Given that the entire competitive task comprises 45 trials, for each dyad, we adopted a cumulative strategy to assess the dynamics of INS growth. Specifically, the INS values from the first to the nth trial were cumulatively summed to track the evolutionary trend of INS throughout the task. Subsequently, an independent samples t-test was employed to assess the disparity in trial-cumulative INS growth between the two conditions. Finally, False Discovery Rate (FDR) corrections were implemented on the resultant p-values.

#### Coupling directionality

To delve deeper into the relationship between the coupling directionality of different roles in the competitive process, we employed Granger Causality Analysis (GCA) based on vector autoregressive modeling to estimate the bidirectionality of the information flow between two time series: the leader and the follower. GCA identifies X as ‘Granger causal’ for Y if the historical values of variable X aid in predicting the future values of variable Y, provided that Y’s own historical values have been considered^81^. In this study, we performed GCA on channels exhibiting significant INS, concentrating on the information flow between leaders and followers (i.e., leader - follower, follower - leader) in both FC and CC. The GCA was computed utilizing the Multivariate Granger Causality (MVGC) toolbox^81^. The order of the autoregressive (AR) model was determined to be 2 based on the Bayesian Information Criterion^82^, with a magnitude similar to that utilized in a prior study^36^. Lastly, we employed a one-sample t-test to assess the disparity between each direction and zero. Paired-sample t-tests were conducted to compare disparities in G causality between the two directions during the competitive task. The effects of Condition and Direction were assessed using a 2 (Condition: FC, CC) × 2 (Direction: leader - follower, follower - leader) mixed-design ANOVA.

### Reporting summary

Further information on research design is available in the Nature Portfolio Reporting Summary linked to this article.

## Supplementary information

Transparent Peer Review file

Supplementary Information

Reporting Summary

## Data Availability

The main data used to generate all the figures and analyses in this article are available in the Science Data Bank repository: (https://doi.org/10.57760/sciencedb.15908)^83^.

## Code availability

In the present study, the fNIRS data collected during the task and at rest were analyzed using the Matlab 2020b platform (Mathworks Inc., Natick, MA, USA). All custom scripts used in this study were written in MATLAB 2020b and are available upon request.
