# Supplementary material for: Frustration reduces interpersonal competition through dynamic interpersonal neural synchronization in dyads
Source: Commun Biol. 2026 Jun 27;9:1362. doi: 10.1038/s42003-026-10573-6 (PMC13677735; doi:10.1038/s42003-026-10573-6)
Supplement: Supplementary file 1 [file 42003_2026_10573_MOESM1_ESM.pdf]

nature portfolio

Peer Review File

Frustration reduces interpersonal competition through dynamic interpersonal neural synchronization in dyads

Corresponding Author: Professor Yingjie Liu

This file contains all reviewer reports in order by version, followed by all author rebuttals in order by version.

Version 0:

Reviewer comments:

Reviewer #1

(Remarks to the Author)
The study investigates the dynamic effects of frustration on interpersonal competition using fNIRS hyperscanning, which is a novel approach. There are different issues to be addressed before considering it for final publication:

1. The literature review appears to be flawly. I would suggest to include more recent studies performed in real word contexts on the same topic (e.g., https://www.frontiersin.org/journals/neuroinformatics/articles/10.3389/fninf.2024.1409322/full).

2. The paper could benefit from a more detailed explanation of the fNIRS hyperscanning method and data analysis techniques.

3. The paper could be strengthened by a more thorough discussion of the implications of the findings for understanding and managing interpersonal competition in real-world settings.

4. The study does not take into account individual differences in frustration tolerance and competitive behavior, which may limit the generalizability of the findings.

5. The conclusion should provide a more detailed discussion of future research directions, such as exploring the impact of different types of frustration on interpersonal competition.

Reviewer #2

(Remarks to the Author)
This work aims to establish whether frustration leads to a decrease in competitive behaviour between two participants playing a reaction time game. In the frustration group participants first play a geometric game which is shown to increase the frustration of the participants and decrease their confidence. This is the correlated with a decrease in the competitive behaviour mediated by an increase in interpersonal neural synchronization within prefrontal cortex responsible for cognitive control.

Originality:
The work is contextualised well and motivated in terms of the limitations of prior work - such as the inability of prior work to establish an attenuation of competition over time. In addition the details of the paradigm appear novel and support detailed new results and perspectives while remaining grounded in established techniques where appropriate.

Quality:
The validity check employed throughout the paradigm is the greatest strength of this work in my opinion and lends a greater degree of confidence in the results as a consequence. For example, being able to establish a clear increase in frustration from the geometric task, established a greater direction of influence from leaders to follows, and track INS over time are important qualities of the paradigm and lead to very rich results being obtained.

The one weakness on quality for me is the absence of any discussion on the mechanism which leads INS in the prefrontal cortex to decrease competitive behaviour. For example, is competition decreased as the frustrated participants are now

using more effort to control for their negative affect deliberately, or is the decrease in competition a more innate behaviour which promotes social cohesion? This is a subtle difference but how deliberate this mechanism is and how innate it is appear to be important considerations.

Clarity:
The clarity of this work to me is the greatest weakness. There are two major points here. Firstly, the captions of this work could use a lot more detail (and figures should include the letters being used as a key in the caption). For figures on the results, there is almost no information summarising the main point of the figure or providing explanation. In the case of Figure 1, no information is given which aids understanding the figure or summarises the paradigm. This would be a good place to summarise the experiment with the visual ques from the images. Secondly, portions of the writing are quite disjointed and incoherent. The introduction in particular repeats itself a lot and not flow neatly between points. For example on the bottom of page 4, the conflicting results on how frustration impacts competitiveness is discussed, then the effect of group frustration during protests is considered, and then on page 6 the effect of stressors on competitive behaviour is revisited. A similar problem occurs in the methods where the preprocessing of the fNIRS data is explained multiple times in different subsections. Finally, while I appreciate that there is conflicting results in the literature (motivating this work), more effort could be done to try to consolidate the current standing of the field in the introduction. For example, on the bottom of page 4 it is mentioned in quick succession that frustration can lead to enhanced competitive behaviour, an avoidance of competitive scenarios and an attempt to preserve self esteem, with relatively little discussion on why these conflicting results might occur. I will also mention that a large portion of page 16 is missing. I do not know why this occurred but clearly this needs to be addressed.

Significance:
Overall I think the quality in the design of the paradigm lends this work a high degree of significance, making communications biology an appropriate journal for the work. I also appreciate the limitations section which demonstrates that future work could clearly build on the approach described in this work - adding to its significance. I do think that some discussion could be added in cases where the results of this work contradict prior results - once again looking at Veltkamp et al (2009) and Radel et al (2011,2014). Why might this work find completely different results from these works? However, due to the depth of validation in the paradigm and precision in the metrics and results I believe the results of this work are reproducible, with the main limitation to the reproducibility coming from the clarity in the description of the methods. Specifically I think Section 2.2.1 could use more clarity in how the points system works for this game, how feedback is given and how confidence levels were determined. While I think this task does induce frustration, the section is too vague to be completely reproducible.

Reviewer #3

(Remarks to the Author)
This paper provides a compelling account of how frustration dynamically modulates interpersonal competition using fNIRS hyperscanning. The authors claim that frustration gradually reduces competitive willingness, accompanied by stage-specific increases in interbrain synchronisation—initially in the frontopolar cortex and later in the dorsolateral prefrontal cortex. Notably, FPC synchronisation mediates the effect of frustration on competition, and a pronounced leader–follower dynamic emerges during task performance. This integration of behavioural and hyperscanning data offers significant insights into cognitive control and social interaction under emotional stress, thereby advancing our understanding of dynamic neural mechanisms in competitive contexts. The experiment is well thought-out, and the paper is clear and concise; however, I have a major concern regarding the selection of the frequency of interest, which the authors need to address. I would appreciate clarification on the following points:

Q1) In the Methods section (paragraph 2.3), the authors state that they used the NIRS_SPM package in MATLAB to further analyse the spatial anatomical location. Could the authors elaborate on this? Specifically, did you register all participants to MNI space? Did you use a specific atlas to determine whether one or more channels were localised in a particular brain region? Please clarify.

Q2) The authors selected the frequency of interest for their analysis by performing t-tests comparing the task to the baseline over the full frequency range. There is a serious risk of double-dipping here, as the frequencies that already showed significant differences between task and baseline might not be independent of the subsequent FC versus CC comparison. This issue was recently raised in a review by Hakim et al. (2023; https://doi.org/10.1016/j.neuroimage.2023.120354), in which the authors warned about the risk of double-dipping and the issues associated with such a method. Could you provide further details on how you controlled for this potential double-dipping when using the FOI to compare FC and CC? For example, did you employ any cross-validation or use independent data for frequency selection? Additionally, the authors state that the results are in line with previous studies; however, the resulting frequency range ([0.12–0.21 Hz]) falls within the range reported by the cited papers but is not identical to the [0.08–0.3 Hz] range. Could you provide further information on this discrepancy? Is it because the selected range coincides with the durations of the task set-up?

Q3) In the Results section (paragraph 3.7), it is not clear whether the t-test results pertain to channel 3 or channel 19. Could you please clarify?

Minor Points:

Q4) There are two citations in the Introduction (line 135: Hari et al., 2018; line 166: Hamilton, 2021) that are not present in the bibliography.

Q5) In the Methods section, when discussing the CBSI, the citation is incorrect. The correct reference for the CBSI method is "Functional near infrared spectroscopy (NIRS) signal improvement based on negative correlation between oxygenated and deoxygenated haemoglobin dynamics."

Q6) In the Methods section (paragraph 2.4.6), do you mean “trail-cumulative” or “trial-cumulative” INS analysis?

Q7) In the Methods section (line 254), the authors refer to image 2A (a plot) instead of image 1, which depicts the task.

Q8) In Figure 1A, although the reference is clear, please label the image with A, B, and C for clarity. There are also two minor spelling errors: “trail” should be “trial” and “coompetition” should be “competition.”

Version 1:

Reviewer comments:

Reviewer #1

(Remarks to the Author)
Many thanks for the revising work. All the issues were properly addressed.

Reviewer #2

(Remarks to the Author)
I have read the author responses in full and reread the full manuscript. My original comments stand that I feel the work has sufficient novelty and will likely shape future work. My original clarity concerns, which were my largest critiques of the original, have been improved on and I thank the authors for their efforts in revising the manuscript. I have no remaining large concerns. Just some minor points:

1. At the bottom of page 7 there is a sentence which ends "..frustration task and thus." which appears to be incomplete
2. The figure captions are improved but I think the figures themselves are still very low resolution and this is very clear in cases where there is text such as on Figure 1 and the legend of Figure 5
3. There is a typo in the caption of Figure 6 at "behaviour(n=35)"
4. Figure 7A seems to have a floating and empty legend square
5. Top of page 30 there is a typo at "consecutive trialS"

Reviewer #3

(Remarks to the Author)
The authors have addressed my previous questions appropriately. The manuscript is now much clearer, the Methods section is complete and easy to follow, and the Discussion is balanced and informative. I support publication of the manuscript in the Communications Biology Journal.

I only suggest one minor clarification, which could be added to the Supplementary Materials section called "Supplementary Analysis 1". Please clarify whether the permutation test was performed by rerunning the full LOSO-CV pipeline for each permutation, including foldwise FOI selection, and confirm that no other preprocessing or model-selection steps were carried out outside the training folds. This would improve the transparency of the validation procedure.

Open Access This Peer Review File is licensed under a Creative Commons Attribution 4.0 International License, which permits use, sharing, adaptation, distribution and reproduction in any medium or format, as long as you give appropriate credit to the original author(s) and the source, provide a link to the Creative Commons license, and indicate if changes were made.
In cases where reviewers are anonymous, credit should be given to 'Anonymous Referee' and the source.
The images or other third party material in this Peer Review File are included in the article's Creative Commons license, unless indicated otherwise in a credit line to the material. If material is not included in the article's Creative Commons license and your intended use is not permitted by statutory regulation or exceeds the permitted use, you will need to obtain permission directly from the copyright holder.
To view a copy of this license, visit https://creativecommons.org/licenses/by/4.0/

Manuscript ID：COMMSBIO-24-7780

Title: Dynamic Interpersonal Neural Synchronization of Frustration Mitigated Interpersonal Competition：a fNIRS-based Hyperscanning Study

Dear editors and reviewers:

Thank you very much for your advice on our manuscript. We also highly appreciate the reviewer's carefulness and conscientiousness, since he/she has given me a number of beneficial suggestions. Those comments are all valuable and very helpful for revising and improving our paper, as well as the important guiding significance to our research. We have studied comments carefully and have made correction which we hope meet with approval. We sincerely hope this manuscript will be acceptable to be published on Communications Biology. Please find the following Response to the comments of the referees:

Responses to Reviewer 1

COMMENT 1: The literature review appears to be flawly. I would suggest to include more recent studies performed in real word contexts on the same topic (e.g., https://www.frontiersin.org/journals/neuroinformatics/articles/10.3389/fninf.2024.1409322/full).

RESPONSE: Thank you for this valuable suggestion. We have carefully reviewed Capotorto et al. (2024) as suggested. We agree that this work represents a critical shift towards evaluating social interactions in ecologically valid settings. Furthermore, inspired by your suggestion, we expanded our literature search to include other recent works by the same research group. We found these studies highly relevant as they emphasize the importance of objective evaluation and dynamic analysis in complex, real-world (or simulated) scenarios. We have incorporated these key insights into the Introduction to highlight the necessity of our dynamic analytical approach. Furthermore, in response to the requirements from the Editor and the reviewers, we have updated the Introduction to incorporate relevant research from recent years,

ensuring a more contemporary and comprehensive literature review.

Please see the details below (see revised manuscript on page 4 and 35)：

However, despite current advances in fNIRS hyperscanning technology, the assessment of INS remains limited by an implicit assumption—namely, that neural activity exhibits temporal stationarity throughout the measurement period (Li et al., 2021). Most existing studies tend to average data across the entire task duration to evaluate INS (Liang et al., 2022; Wang et al., 2022). In contrast, interpersonal competition is essentially a highly dynamic social interaction process involving complex, real-time adjustments of emotional, cognitive, and social strategies (Hamilton, 2021; Jiang et al., 2021). Furthermore, from a hyperscanning perspective, the interaction patterns between two participants after jointly experiencing frustration may exhibit dynamic characteristics that are distinct from those in single-person contexts. Research has indicated that jointly experiencing frustration not only influences individual emotional regulation through social referencing (Miao et al., 2021; Decety & Meyer, 2008) but also prompts participants to gradually adjust their goal expectations and motivational structures (Robinson & Moeller, 2014; Vansteenkiste & Ryan, 2013). Such shared experiences may trigger an adaptive psychological adjustment process: as frustration persists, individuals may gradually shift from their initial competitive focus toward maintaining psychological equilibrium, adopting more conservative behavioral strategies (Yuan et al., 2015). Therefore, the impact of frustration on interpersonal competition is not a monolithic instantaneous effect, but rather a dynamic mitigation process encompassing multi-stage changes (Dugré & Potvin, 2023). This perspective, focusing on ecological validity and temporal dynamics, aligns closely with recent academic calls to evaluate social interaction behavior in real-world contexts (Capotorto et al., 2024; Ronca et al., 2025). In summary, to more accurately and comprehensively understand the dynamic neural synchronization mechanisms during interpersonal competition under frustration, the present study employs a more refined approach to explore the dynamic

fluctuations of INS throughout the competitive task.

5.Conclusion

Within the framework of two-person neuroscience, we used fNIRS-based hyperscanning to integrate relevant parameters at the behavioural and brain levels to better understand the dynamic effects of frustration on interpersonal competition. We found that when dyads perform an interpersonal competition task following a frustration experience, the task-related INS of the FPC and dlPFC emerge successively with a gradual decrease in willingness to compete. This suggests that frustration dynamically reduces interpersonal competition, potentially through changes in INS. We also found that the INS in FPC was significantly and positively correlated with competition index and mediated the effect of frustration on competition performance. We also identified roles in interpersonal competition and found that leaders exerted greater influence on followers. This suggests that role asymmetry within dyads play a critical role in interpersonal competition, particularly in the context of frustration. These results provide interpersonal neural evidence for interpersonal competitive interactions in the context of frustration and contribute to a better understanding of the dynamic mitigating effects of frustration experiences on competition. Looking ahead, several key directions merit further investigation. For instance, the impact of the heterogeneity of frustration types on interpersonal competition warrants deeper exploration. Furthermore, subsequent studies should strive to validate these findings in more ecologically valid real-world settings.

New References:

Capotorto, R., Ronca, V., Sciaraffa, N., Borghini, G., Di Flumeri, G., Mezzadri, L., ... & Aricò, P. (2024). Cooperation objective evaluation in aviation: Validation and comparison of two novel approaches in simulated environment. Frontiers in Neuroinformatics, 18, 1409322.

Ronca, V., Castagneto Gissey, L., Bellini, M. I., Iodice, A., Aricò, P., Di Flumeri, G., ... & Borghini, G. (2025). Mutual information-based teamwork evaluation in real-world environments: an exploratory investigation with professional surgeons. Frontiers in Network Physiology, 5, 1608824.

COMMENT 2: The paper could benefit from a more detailed explanation of the fNIRS hyperscanning method and data analysis techniques.

RESPONSE: We are grateful for your constructive suggestion. We entirely agree that comprehensive methodological transparency and a rigorous data analysis pipeline are fundamental to the validity of hyperscanning research. In accordance with your comments, we have extensively refined the "Methods" section of the revised manuscript. Specifically, we have added substantial technical details regarding fNIRS data acquisition, preprocessing standards, and the computational logic for Interpersonal Neural Synchronization (INS):

Detailed Synchronization Mechanism for Hyperscanning: We have provided additional technical specifications regarding the synchronization of dyadic data acquisition. By utilizing an external trigger system to simultaneously send marking signals to both acquisition units, we ensured millisecond-level temporal alignment at the onset of the experiment and at each trial transition. This precision is a critical prerequisite for the subsequent calculation of inter-brain synchronization.

Refined Data Preprocessing Pipeline:We have expanded the description of the preprocessing steps in the revised manuscript.

Enhanced INS Calculation Logic:We have provided a detailed rationale for employing Wavelet Transform Coherence (WTC) for our analysis. Compared to traditional cross-correlation methods, WTC enables the decomposition of signals in both the time and frequency domains simultaneously. This capability is essential for capturing the dynamic interpersonal neural synchronization patterns that are a central focus of this study.

Optimized Selection of Frequency of Interest (FOI): To ensure statistical rigor and mitigate the potential risk of double-dipping , we have improved our method for determining the FOI. Specifically, we introduced a Leave-One-Subject-Out Cross-Validation (LOSO-CV) strategy. This approach ensures that the frequency selection process is independent of the subsequent group-level statistical comparisons, thereby enhancing the robustness of our findings.

Strengthened Statistical Validation via Permutation Testing: We have provided a more meticulous description of the permutation test procedure used to exclude spurious correlations. By comparing the INS of genuine interacting dyads against a null distribution generated from 1000 iterations of random pairings, we demonstrated that the observed synchronization was specifically driven by actual social interaction rather than shared task environments or stochastic noise.

Please see the details below (see revised manuscript on page 11-12 and 14-17)：

2.3 fNIRS data acquisition

Two identical fNIRS systems (LIGHTNIRS; Shimadzu Co., Japan) were utilized to record the brain signals of two participants in a dyad during the experiment. This fNIRS system has a sampling rate of 13.3 Hz and three near-infrared wavelengths (780 nm, 805 nm, and 830 nm). By utilizing an external trigger system to simultaneously send marking signals to both acquisition units, we ensured millisecond-level temporal alignment at the onset of the experiment and at each trial transition. For each participant, an 8 × 2 probe patch was positioned to cover the bilateral prefrontal cortex, resulting in a total of 22 recording channels (CHs) (Fig. 2C). Each patch was equipped with 8 emitters and 8 detectors. The distance between emitters and detectors was approximately 3 cm. The placement of the emitter and detector followed the 10–20 system (Okamoto et al., 2004). The lower row of optodes was positioned along the Fp1-Fpz-Fp2 line. A 3D magnetic space digitizer (FASTRAK; Polhemus, USA) was employed to assess the anatomical positions of optodes and channels for each participant, using NZ, CZ, AL, and AR as reference points (Singh et al., 2005; Tsuzuki et al., 2007). The spatial anatomical locations were further analyzed using the NIRS_SPM MATLAB package (Ye et al., 2009), whereby individual coordinates were registered to the MNI space. Anatomical labels were determined based on the Brodmann and LPBA40 atlases. Detailed MNI information for all channels is provided in the Supplementary Material .

2.4.3 fNIRS data analysis

Preprocessing

The fNIRS data underwent preprocessing through the Homer2 toolbox and a customized MATLAB-based script in MATLAB 2021a (Mathworks Inc., Natick, MA, USA). Initially, the raw light intensity signal was converted into optical density (OD). Then, its quality was visually assessed according to the criterion of detector saturation or insufficient optical coupling (i.e., the absence of a heartbeat frequency of ~ 1 Hz in the signal's power spectrum). Any channels that failed to meet the criterion were deleted. Ultimately, 98.8% of the channels were retained for further analysis. The channels exhibiting poor signal quality were replaced with the average values of neighboring channels. If more than one-third of the channels exhibited poor quality, the dyad was excluded (Lu et al., 2020; Zhao et al., 2022). Second, the principal component analysis (PCA) was subsequently employed to eliminate signal artifacts resulting from spontaneous blood flow oscillations or other systematic factors (Zhang et al., 2016). A conservative variance threshold of 80% was applied to remove noise. Correlation-based signal improvement (CBSI) was utilized to mitigate head motion artifacts (Cui et al., 2010). In line with previous studies, the data were neither filtered nor detrended for task-related INS analyses (Cui et al., 2012; Cheng et al., 2015). Finally, the optical density data for each channel were transformed into concentrations of oxyhemoglobin (HbO), deoxyhemoglobin (Hbr), and total hemoglobin (Hbt) using the modified Beer-Lambert law. Given that HbO signaling is more sensitive to changes in cerebral blood flow (Hoshi, 2007) and has been extensively utilized in fNIRS hyperscanning studies (Pan et al., 2017; Li et al., 2022), we focused on analyzing HbO signals.

INS data analysis

Following preprocessing, the Wavelet Transform Coherence (WTC) method was applied on each channel to compute the INS of interacting individuals (Grinsted et al., 2004). In contrast to correlation methods, WTC is regarded as more appropriate due to its normalization of the signal amplitude in accordance with each time window,

rendering it less susceptible to motion-induced transient spikes (Nozawa et al., 2016). Compared to traditional cross-correlation methods, WTC enables the decomposition of signals in both the time and frequency domains simultaneously. Channel coherence was calculated utilizing the WTC package within a bespoke MATLAB script. The WTC of HbO time series i (m) and j (m) at wavelet scale s was determined according to the following formula:

$$\mathrm{WTC}(m,s)=\frac{\left|\left\langle s^{-1}W^{ij}(m,s)\right\rangle\right|^{2}}{\left|\left\langle s^{-1}W^{i}(m,s)\right\rangle\right|^{2}\left|\left\langle s^{-1}W^{j}(m,s)\right\rangle\right|^{2}}$$

where m, s, and W denote time, wavelet scale, and the continuous wavelet transform, respectively. To identify the task-related frequency band while avoiding the issue of double-dipping (Hakim et al., 2023), we employed a Leave-One-Subject-Out Cross-Validation (LOSO-CV) method. This approach integrates frequency selection with classification modeling to ensure the independence of the analysis process (detailed procedures are provided in Supplementary Analysis 1). The results showed increased INS for frequencies ranging between 0.12 and 0.21 Hz. This frequency band excludes low-frequency fluctuations (below 0.01 Hz) and high-frequency physiological signals, such as cardiac pulsation (~ 1 Hz) and respiration (~ 0.3 Hz) (Pierro et al., 2014; Yücel et al., 2021). In this FOI, each channel's INS was computed individually for baseline and interpersonal interaction tasks, and then the latter was subtracted from the former to obtain the task-related INS (in comparison to the baseline). Following the acquisition of the INS values for each dyad and channel, single-sample t-tests were employed to examine whether any of the channels showed a significant enhancement of INS during cooperation, aiming to mitigate the risk of false findings and enhance the robustness of the results. The channels of interest in this investigation comprised all those that exhibited significant INS. The task-specific channels were then subjected to independent samples t-test analyse and two-way analysis of variance to assess whether there existed a difference between the FC and CC.

2.4.5 Validating the INS through permutation test

To ensure that the higher INS observed in the frustration-conditioned

interpersonal competition task stemmed from the joint interaction task performed by the two-person group members, independent of other factors, we implemented a validation analysis via recruiting a trial of pairwise randomized replacements. This rationale stems from the expectation that if the higher INS was solely attributed to the processing of the task material itself, it would vanish after randomizing the participants' pairs, as there would be no interaction between them. It is noteworthy that new dyadic pairs of subjects did not have exactly the same duration in the competition task due to individual differences in reaction times, but wavelet consistency analyses require that the durations of the two time series be equal (Grinsted et al., 2004). Therefore, we clipped the data for each new dyad to ensure the time series were consistent (Reindl et al., 2018). The INS analyses were subsequently re-run, and a series of independent samples t-tests were conducted on the two newly formed data sets. This replacement process was iterated 1000 times to generate the null distribution (t-value), and the level of significance was determined by comparing the original paired INS against the 1000 randomly paired INS ($p < 0.05$).

COMMENT 3: The paper could be strengthened by a more thorough discussion of the implications of the findings for understanding and managing interpersonal competition in real-world settings.

RESPONSE: We are grateful for your insightful suggestion. We entirely agree that a more comprehensive discussion of the practical implications of our findings significantly enhances the broader impact and relevance of this study. Accordingly, we have added a new subsection titled "4.4 Practical Implications for Real-world Interpersonal Competition" to the Discussion.

Please see the details below (see revised manuscript on page 30)：

4.4 Practical Implications for Real-world Interpersonal Competition

The present study provides empirical insights into the management of interpersonal competition in real-world environments. First, the finding that shared

frustration suppresses competitive impulses by enhancing INS in the FPC suggests that constructing a cognitive framework of shared adversity in deadlocked situations can effectively activate behavioral regulation, thereby mitigating irrational interpersonal competition (Miao et al., 2021). Second, the evolution of competition mitigation from early-stage behavioral inhibition (FPC-based INS) to late-stage intention inference (dlPFC-based INS) indicates that conflict intervention strategies should be staged. In the initial phase of competition, priority should be given to reinforcing self-control through rule-based constraints. In contrast, during sustained interaction, the focus should shift toward enhancing informational transparency to facilitate perspective-taking, enabling a transition from maladaptive rivalry to strategic coordination (Zhang et al., 2023; Kadohisa et al., 2022). Finally, our GCA results demonstrate that the directional influence of leaders is amplified within frustrating competitive environments. This underscores the critical importance of training leaders to maintain composed and rational social signals, which can more effectively synchronize the decision-making logic of the entire team and prevent collective lapses into irrational competition (Jiang et al., 2015; Pan et al., 2018).

COMMENT 4: The study does not take into account individual differences in frustration tolerance and competitive behavior, which may limit the generalizability of the findings.

RESPONSE: We sincerely appreciate your insightful suggestion. We fully agree that trait-level frustration tolerance and competitive predisposition are critical moderating variables that may influence both interpersonal neural synchronization (INS) and behavioral responses. The primary objective of the present study was to investigate the dynamic impact of experimentally induced, state-level frustration on INS. In response to your suggestion, we have expanded the discussion in the "Limitations" section (Section 4.5) of the revised manuscript. We now explicitly address the potential moderating roles of these individual characteristics, aiming to further elucidate how individual traits interact with situational frustration to shape the dynamic processes of interpersonal competition.

Please see the details below(see revised manuscript on page 34-35):

4.4 Limitations

The limitations of the current study should also be addressed. First, our fNIRS optical probe set covered only the prefrontal cortex of the brain, leaving other regions unexplored. Previous studies have found that the temporoparietal regions also cause increased INS during competive tasks (Xue et al., 2018; Czeszumski et al., 2022). Future research could further investigate the role of other brain regions in competive tasks by measuring the whole brain. Secondly, according to Self-Determination Theory, individuals suffering from prolonged frustration are more inclined to take measures to compensate, such as finding substitutes for needs, relaxing self-control, and developing undesirable behaviors (Vansteenkiste & Ryan, 2013). It has also been found that persistent frustration reduces an individual's level of motivation to engage in subsequent tasks (Fang et al., 2020). Future research could further compare behavioral and neural differences between short-term and long-term frustration in competive tasks. Finally, the current study did not fully account for individual differences within the dyads. Individual traits, such as frustration tolerance and competitive orientation, may significantly modulate an individual’s perception of setbacks and subsequent patterns of interpersonal competition (Yu et al., 2016). Future research should specifically investigate the moderating effects of these individual characteristics, integrating other psychosocial factors — such as social value orientation (Bogaert et al., 2008) and psychological resilience (Troy et al., 2023)— into a comprehensive analysis. Such efforts would provide a more nuanced understanding of the inter-individual variability in co-experienced frustration and competitive behavior.

COMMENT 5: The conclusion should provide a more detailed discussion of future research directions, such as exploring the impact of different types of frustration on interpersonal competition.

RESPONSE: We are grateful for your insightful suggestion. We fully agree that

delineating future research directions within the conclusion—specifically exploring the heterogeneity of frustration—is vital for enriching the theoretical framework of this research area. In accordance with your suggestion, we have revised and expanded the Conclusion (Section 5) to incorporate these forward-looking perspectives.

Please see the details below(see revised manuscript on page 35)：

5. Conclusion

Within the framework of two-person neuroscience, we used fNIRS-based hyperscanning to integrate relevant parameters at the behavioural and brain levels to better understand the dynamic effects of frustration on interpersonal competition. We found that when dyads perform an interpersonal competition task following a frustration experience, the task-related INS of the FPC and dlPFC emerge successively with a gradual decrease in willingness to compete. This suggests that frustration dynamically reduces interpersonal competition, potentially through changes in INS. We also found that the INS in FPC was significantly and positively correlated with competition index and mediated the effect of frustration on competition performance. We also identified roles in interpersonal competition and found that leaders exerted greater influence on followers. This suggests that role asymmetry within dyads play a critical role in interpersonal competition, particularly in the context of frustration. These results provide interpersonal neural evidence for interpersonal competitive interactions in the context of frustration and contribute to a better understanding of the dynamic mitigating effects of frustration experiences on competition. Looking ahead, several key directions merit further investigation. For instance, the impact of the heterogeneity of frustration types on interpersonal competition warrants deeper exploration. Furthermore, subsequent studies should strive to validate these findings in more ecologically valid real-world settings.

Responses to Reviewer 2

COMMENT 1: This work aims to establish whether frustration leads to a decrease in

competitive behaviour between two participants playing a reaction time game. In the frustration group participants first play a geometric game which is shown to increase the frustration of the participants and decrease their confidence. This is the correlated with a decrease in the competitive behaviour mediated by an increase in interpersonal neural synchronization within prefrontal cortex responsible for cognitive control.

RESPONSE: We sincerely thank you for the positive evaluation and thoughtful comments. Your insights were highly valuable in improving both the clarity and rigor of our manuscript. Below, we provide a detailed point-by-point response to each of your comments, along with a summary of the corresponding revisions made to the manuscript.

COMMENT 2: Originality:The work is contextualised well and motivated in terms of the limitations of prior work - such as the inability of prior work to establish an attenuation of competition over time. In addition the details of the paradigm appear novel and support detailed new results and perspectives while remaining grounded in established techniques where appropriate.

RESPONSE: We sincerely appreciate your recognition of the originality and conceptual significance of our study. As you rightly pointed out, our research was motivated by a critical gap in the existing literature — specifically, the limited understanding of the dynamics through which frustration impacts interpersonal competition.

In this study, we carefully designed our experimental paradigm based on the limitations of prior research, especially the lack of understanding of how frustration dynamically affects competitive behavior over time. We used a novel geometric game task to induce frustration, which not only effectively increased participants' frustration but also decreased their confidence. This design allowed us to quantify the level of frustration and analyze its specific impact on subsequent competitive behavior.

We also employed fNIRS-basedc hypersanning to record participants' neural activities synchronously. Our results revealed a dynamic pattern of interpersonal neural synchronization (INS) in the prefrontal cortex (PFC) during the competition task. Early in the task, INS was observed in the frontopolar cortex (FPC), and later in

the dorsolateral prefrontal cortex (dlPFC). This dynamic change of INS provides new insights into the neural mechanisms of how frustration affects competition.

We are delighted that our work has been recognized for its originality. We will continue to explore the interaction between frustration and other emotional factors in future research, as well as the differences in neural synchronization mechanisms across various competitive tasks, with the aim of making further contributions to this field.

COMMENT 3: Quality: The validity check employed throughout the paradigm is the greatest strength of this work in my opinion and lends a greater degree of confidence in the results as a consequence. For example, being able to establish a clear increase in frustration from the geometric task, established a greater direction of influence from leaders to follows, and track INS over time are important qualities of the paradigm and lead to very rich results being obtained.

The one weakness on quality for me is the absence of any discussion on the mechanism which leads INS in the prefrontal cortex to decrease competitive behaviour. For example, is competition decreased as the frustrated participants are now using more effort to control for their negative affect deliberately, or is the decrease in competition a more innate behaviour which promotes social cohesion? This is a subtle difference but how deliberate this mechanism is and how innate it is appear to be important considerations.

RESPONSE: We sincerely thank you for these profound and thought-provoking comments regarding the underlying mechanism of competition mitigation. We fully agree that distinguishing between "deliberate effort" and "innate adaptation" is essential for a comprehensive elucidation of the neurocognitive basis of our findings.

In the revised manuscript, we have added a dedicated paragraph at the end of Section 4.1 to explore this mechanism. We do not view these two modes as mutually exclusive; rather, based on our results, we propose that the reduction in competitive behavior reflects a dynamic evolution from executive resource regulation to intentional alignment:

Early Stage: We propose that the initial reduction in competition is primarily driven by deliberate cognitive effort. According to the limited cognitive resource theory (Hofmann et al., 2009), the allocation of substantial executive resources to the frontopolar cortex (FPC) for monitoring and suppressing emotions triggered by frustration reallocates the cognitive resources originally intended to drive aggressive competitive responses. Therefore, at this stage, the interpersonal neural synchronization (INS) in the FPC serves as a marker for effortful, top-down co-regulatory control.

Late Stage: As the interaction persists, the mechanism shifts toward an innate-like social adaptation. The shared experience of frustration serves as a "social glue" (Bastian et al., 2018), facilitating emotional bonding and interpersonal cohesion. At this stage, the INS in the dorsolateral prefrontal cortex (dlPFC) serves as a neural marker for intentional alignment, enabling dyads to mutually understand and predict the intention to mitigate competition. Consequently, the reduction in competition evolves from deliberate cognitive regulation into an adaptive social behavior that fosters cohesion and maintains social stability.

We believe this dual-mode explanation provides a robust mechanistic framework for understanding how INS drives the sustained attenuation of interpersonal competition.

Please see the details below(see revised manuscript on page 30-31):

In summary, we propose that this process reflects a dynamic evolution from executive resource regulation to intentional alignment. During the early stage of the task, the enhanced synchronization in the FPC reflects interpersonal co-regulatory control directed at frustration. This regulatory process possesses distinct high-load cognitive attributes. According to the limited cognitive resource theory (Hofmann et al., 2009), when the brain allocates substantial resources to the FPC for monitoring and suppressing negative emotions triggered by frustration, the cognitive resources originally intended to drive aggressive competitive responses are reallocated, leading to the decline in early competitive willingness. As the task persists, the shared

experience of frustration facilitates emotional bonding between individuals, which in turn promotes intentional alignment mediated by the dlPFC. Social psychological research indicates that shared negative experiences, particularly in frustrating contexts, can enhance interpersonal cohesion (Bastian et al., 2018). At this stage, the INS in the dlPFC serves as a neural marker for dyads to mutually understand the intention to mitigate competition. The reduction in competition no longer relies solely on deliberate cognitive regulation but is transformed into an adaptive social behavior that fosters cohesion. This dynamic process provides a neurocognitive mechanistic explanation for understanding how INS drives the mitigation of competitive behavior.

New References:

Hofmann, W., Friese, M., & Strack, F. (2009). Impulse and self-control from a dual-systems perspective. Perspectives on psychological science, 4(2), 162-176.

Bastian, B., Jetten, J., Thai, H. A., & Steffens, N. K. (2018). Shared adversity increases team creativity through fostering supportive interaction. *Frontiers in Psychology*, 9, 2309.

COMMENT 4: Clarity: The clarity of this work to me is the greatest weakness. There are two major points here. Firstly, the captions of this work could use a lot more detail (and figures should include the letters being used as a key in the caption). For figures on the results, there is almost no information summarising the main point of the figure or providing explanation. In the case of Figure 1, no information is given which aids understanding the figure or summarises the paradigm. This would be a good place to summarise the experiment with the visual ques from the images. Secondly, portions of the writing are quite disjointed and incoherent. The introduction in particular repeats itself a lot and not flow neatly between points. For example on the bottom of page 4, the conflicting results on how frustration impacts competitiveness is discussed, then the effect of group frustration during protests is considered, and then on page 6 the effect of stressors on competitive behaviour is revisited. A similar problem occurs in the methods where the preprocessing of the fNIRS data is explained multiple times in different subsections. Finally, while I

appreciate that there is conflicting results in the literature (motivating this work), more effort could be done to try to consolidate the current standing of the field in the introduction. For example, on the bottom of page 4 it is mentioned in quick succession that frustration can lead to enhanced competitive behaviour, an avoidance of competitive scenarios and an attempt to preserve self esteem, with relatively little discussion on why these conflicting results might occur. I will also mention that a large portion of page 16 is missing. I do not know why this occurred but clearly this needs to be addressed.

COMMENT4-1： The clarity of this work to me is the greatest weakness. There are two major points here. Firstly, the captions of this work could use a lot more detail (and figures should include the letters being used as a key in the caption). For figures on the results, there is almost no information summarising the main point of the figure or providing explanation. In the case of Figure 1, no information is given which aids understanding the figure or summarises the paradigm. This would be a good place to summarise the experiment with the visual ques from the images.

RESPONSE: We sincerely appreciate your constructive feedback regarding the clarity of our figure captions. We fully agree that detailed and self-explanatory captions are essential for readers to navigate the complex hyperscanning experimental design and the dynamic neural mechanisms identified in this study. In response to your comment, we have thoroughly revised and expanded the captions for all figures.

Please see the details below(see revised manuscript on page 19-28)：

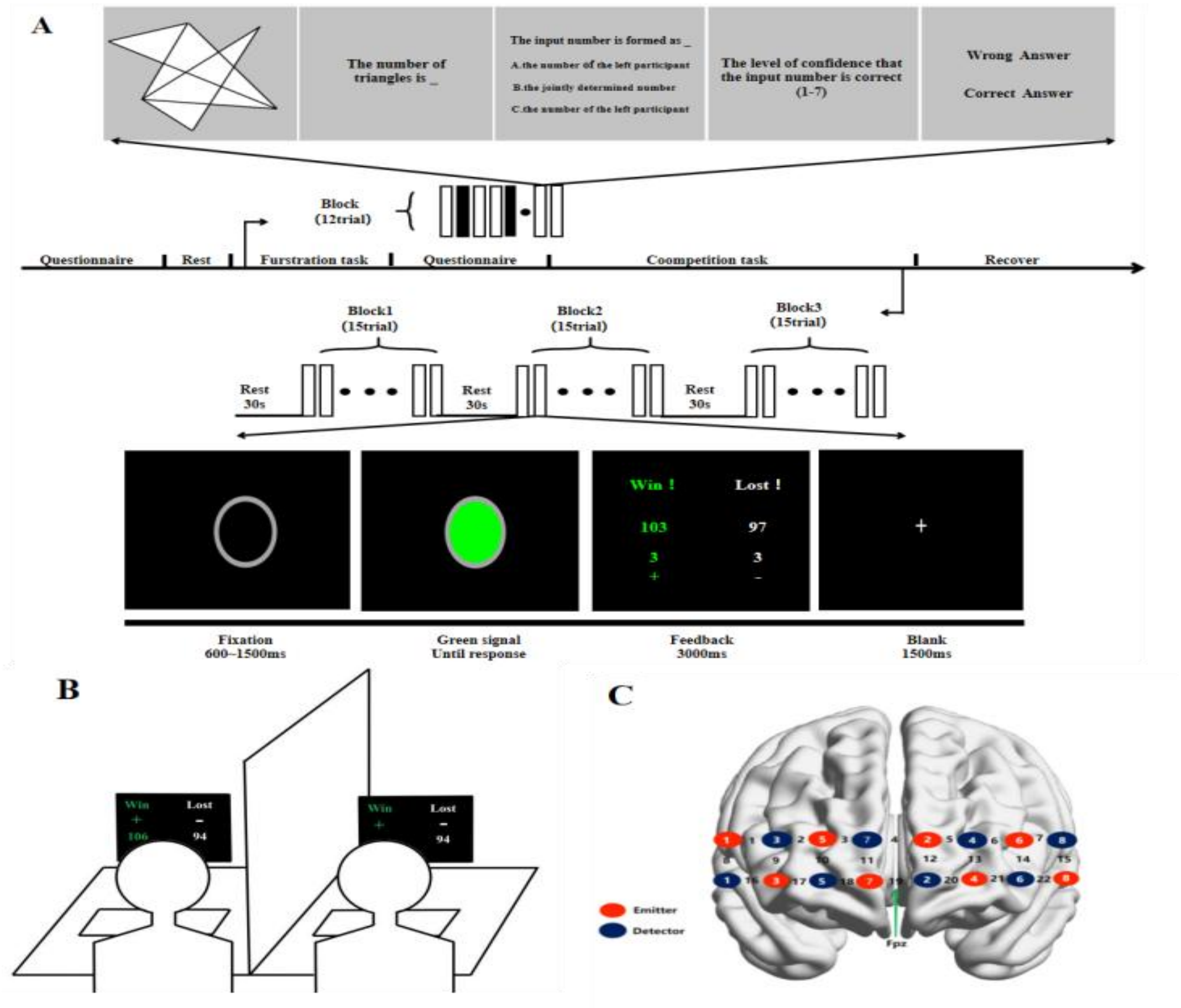

Fig. 1. Experimental paradigm and hyperscanning setup. (A) Experimental timeline and trial procedures. The experiment proceeded through a baseline rest, a frustration task, and a competitive task, with questionnaires administered at key intervals. (B) Hyperscanning configuration. Two participants sat face-to-face on either side of a divider, each using an independent monitor and keyboard to ensure social interaction while preventing visual interference of the other's screen. (C) fNIRS optode probe set. A 22-channel configuration was placed over the prefrontal cortex (PFC). Red circles represent emitters and blue circles represent detectors.

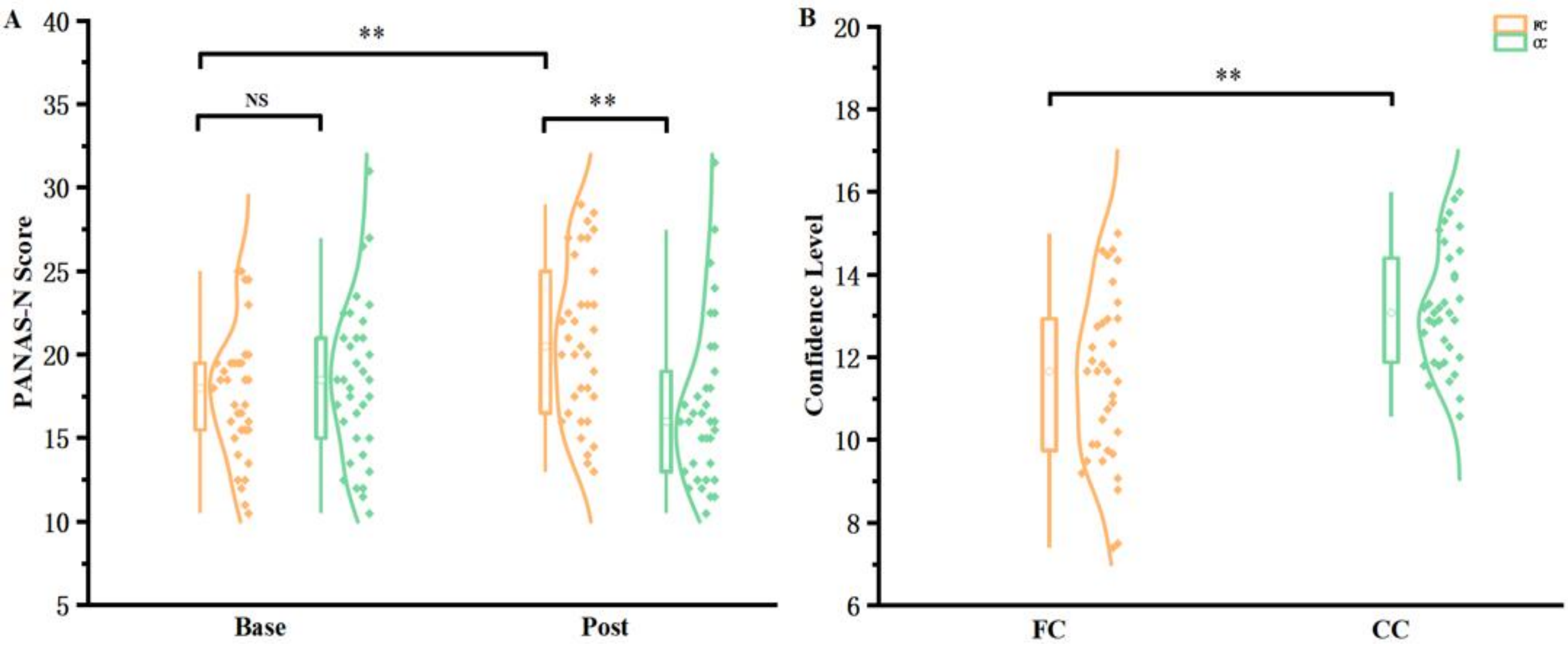

Fig. 2. Behavioral and psychological validation of the frustration induction. (A) Negative affect scores. The violin plots illustrate the Negative Affect (PANAS-N) scores at baseline (Base) and after the task (Post) for both the FC and the CC. A significant increase in negative affect was observed only in the FC following the frustration task, while no significant change was found in the CC. (B) Confidence levels during the tasks. Participants in the FC reported significantly lower confidence in their task performance compared to those in the CC group. These behavioral results serve as a manipulation check, confirming that the frustration task successfully induced a negative emotional state and reduced subjective performance confidence in the FC.

Notes: For the violin plots, the inner box represents the interquartile range (IQR), the white dot or horizontal line represents the median, and the whiskers extend to 1.5 times the IQR. $^{**}p < 0.01$, NS = not significant. FC = Frustration Condition; CC = Control Condition.

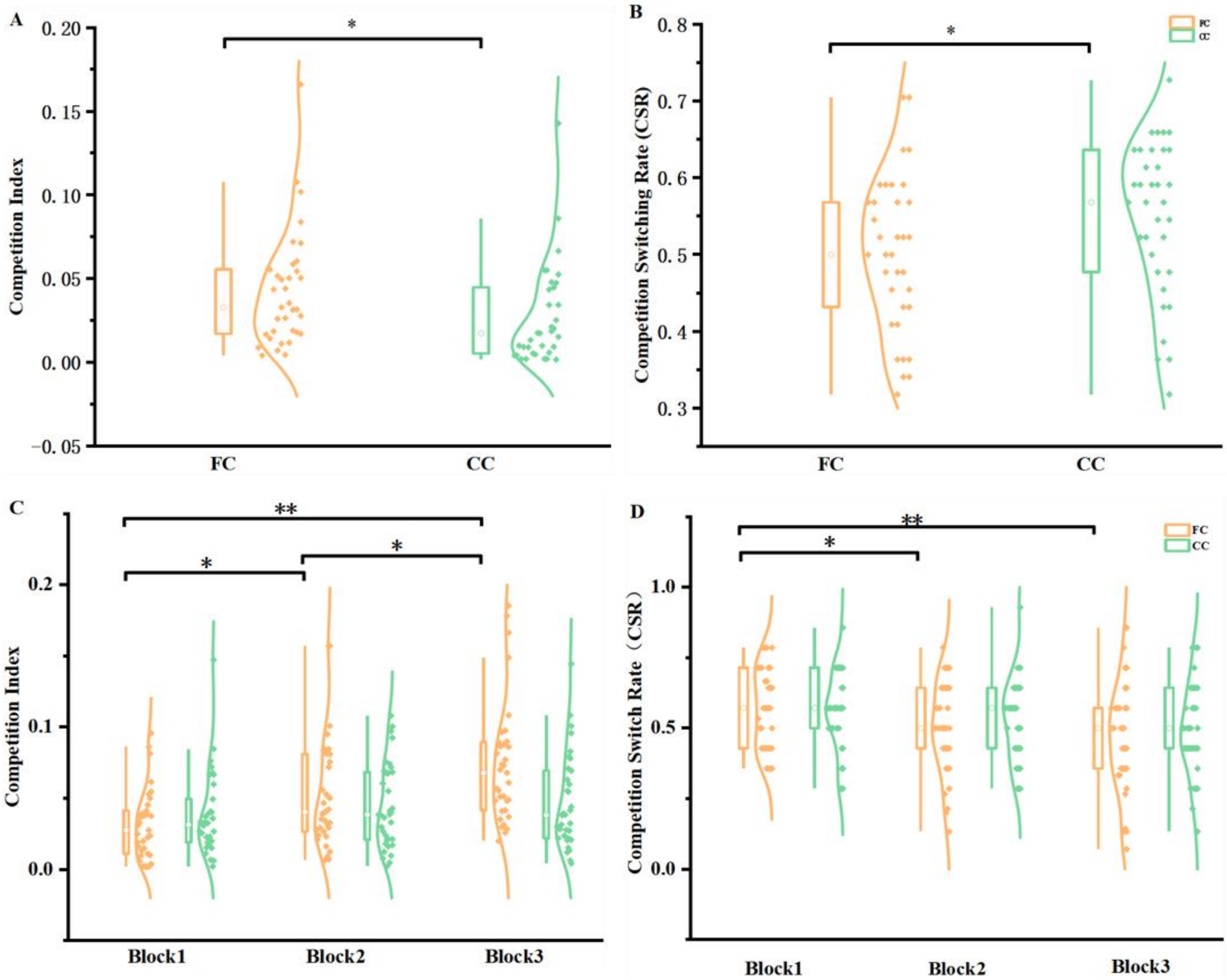

Fig. 3. Behavioral performance and dynamic progression during the competitive task.(A) Competition index. Independent samples t-tests showed that the FC exhibited a significantly higher competition index compared to the CC, indicating a divergence in competitive behavior patterns after frustration induction. (B) Competition Switching Rate (CSR). The FC showed a significantly reduced CSR compared to the CC. (C) Dynamic changes in competition index across blocks. A two-way ANOVA revealed a significant Condition × Block interaction. In the FC, the competition index increased significantly from Block 1 to Block 3, whereas no such temporal change was observed in the CC. (D) Dynamic changes in CSR across blocks. A significant main effect of Block was found, with CSR gradually decreasing from Block 1 to Block 3 across both conditions, reflecting a general stabilization of strategy over time. These findings indicate that frustration does not exert a static impact but instead attenuates interpersonal competitive interactions dynamically. *$p < 0.05$, **$p < 0.01$.

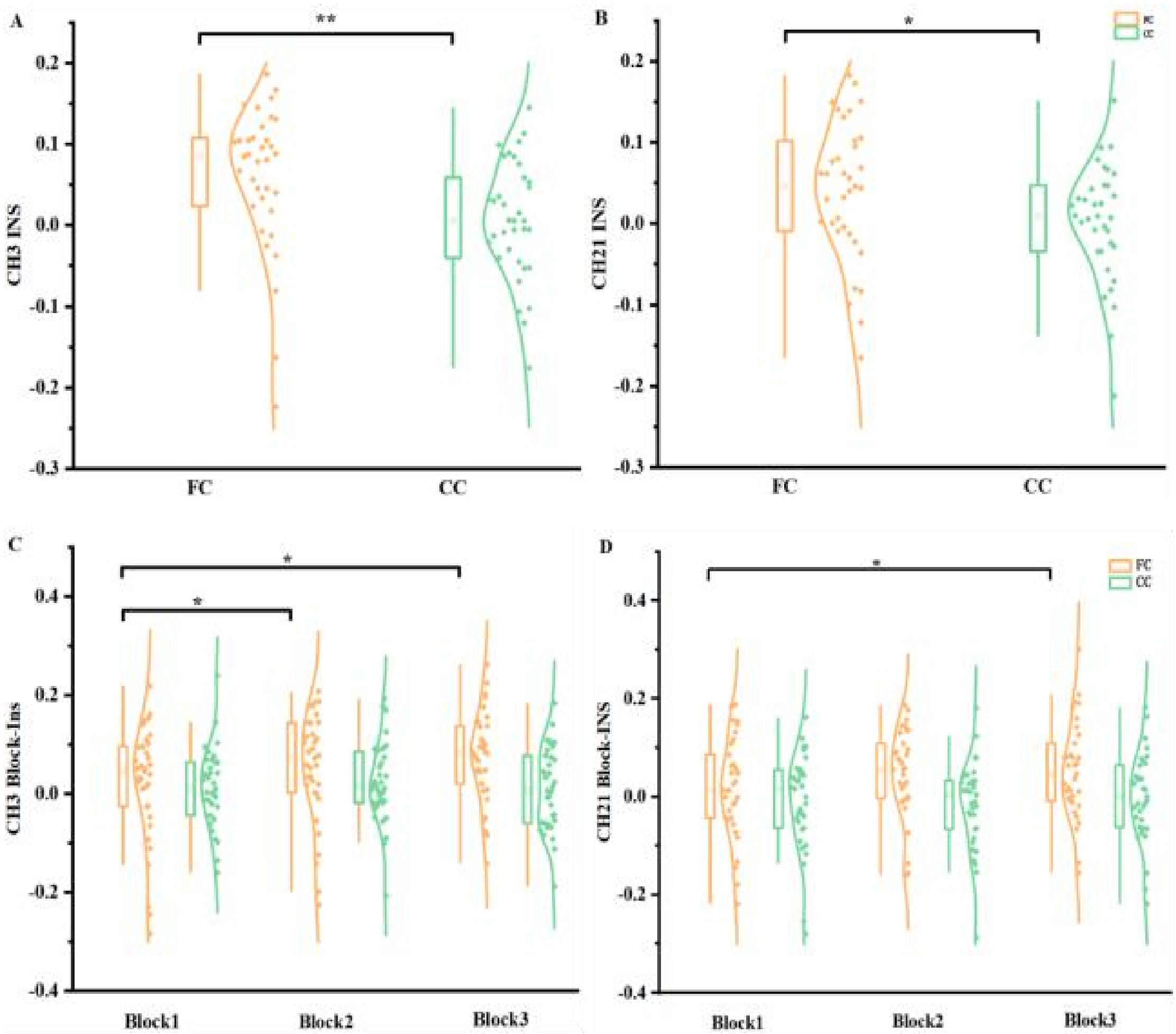

Fig. 4. INS and dynamic progression during the competitive task. (A) INS in CH3. Independent samples t-tests showed that FC exhibited significantly higher INS in CH3 compared to CC. (B) INS in CH21. Independent samples t-tests showed that FC exhibited significantly higher INS in CH21 compared to CC. (C) Dynamic changes in CH3 INS across blocks. Paired-samples t-tests within FC revealed that INS in CH3 increased significantly from Block 1 to the subsequent blocks. In contrast, no such temporal increase was observed in CC.
(D) Dynamic changes in CH21 INS across blocks. In FC, INS in CH21 showed a significant increase in the Block 3 compared to the Block 1, whereas INS in CC remained stable across the entire task. These findings demonstrate that frustration significantly enhances neural synchronization in both the FPC and dlPFC during competition. INS is not static but evolves dynamically, showing a progressive upward trend in frustrated dyads as the interaction unfolds. $*p < 0.05$, $**p < 0.01$.

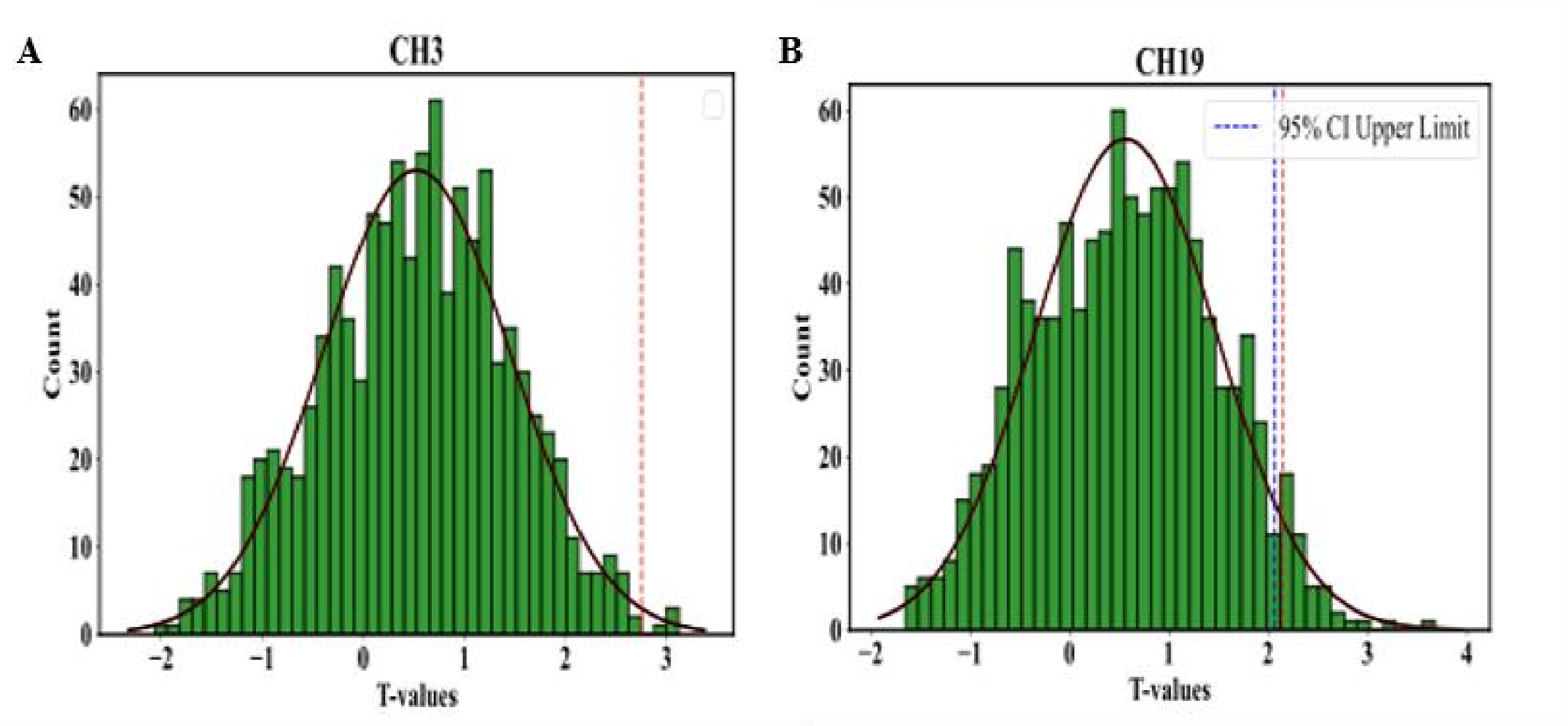

Fig. 5. Validation of task-related INS via permutation tests. (A) Permutation results for CH3. The histogram illustrates the null distribution of t-values generated from 1,000 random dyadic permutations. The observed t-value from the actual dyads (indicated by the red dashed line) falls significantly within the upper 5% tail of the distribution (p<0.001), confirming that the INS in CH3 is specific to real interacting dyads. (B) Permutation results for CH21 (dlPFC). The observed t-value for CH21 (red dashed line) is significantly higher than the t-values derived from 1,000 random permutations (p<0.001), falling beyond the 95% confidence interval upper limit (blue dashed line). These validation analyses demonstrate that the increased INS in the FPC and dlPFC during competition is a product of actual interpersonal interaction rather than a byproduct of similar task demands or random chance.

Notes: Red dashed lines represent the observed t-values from real dyads. The blue dashed line represents the 95% confidence interval upper limit of the null distribution.

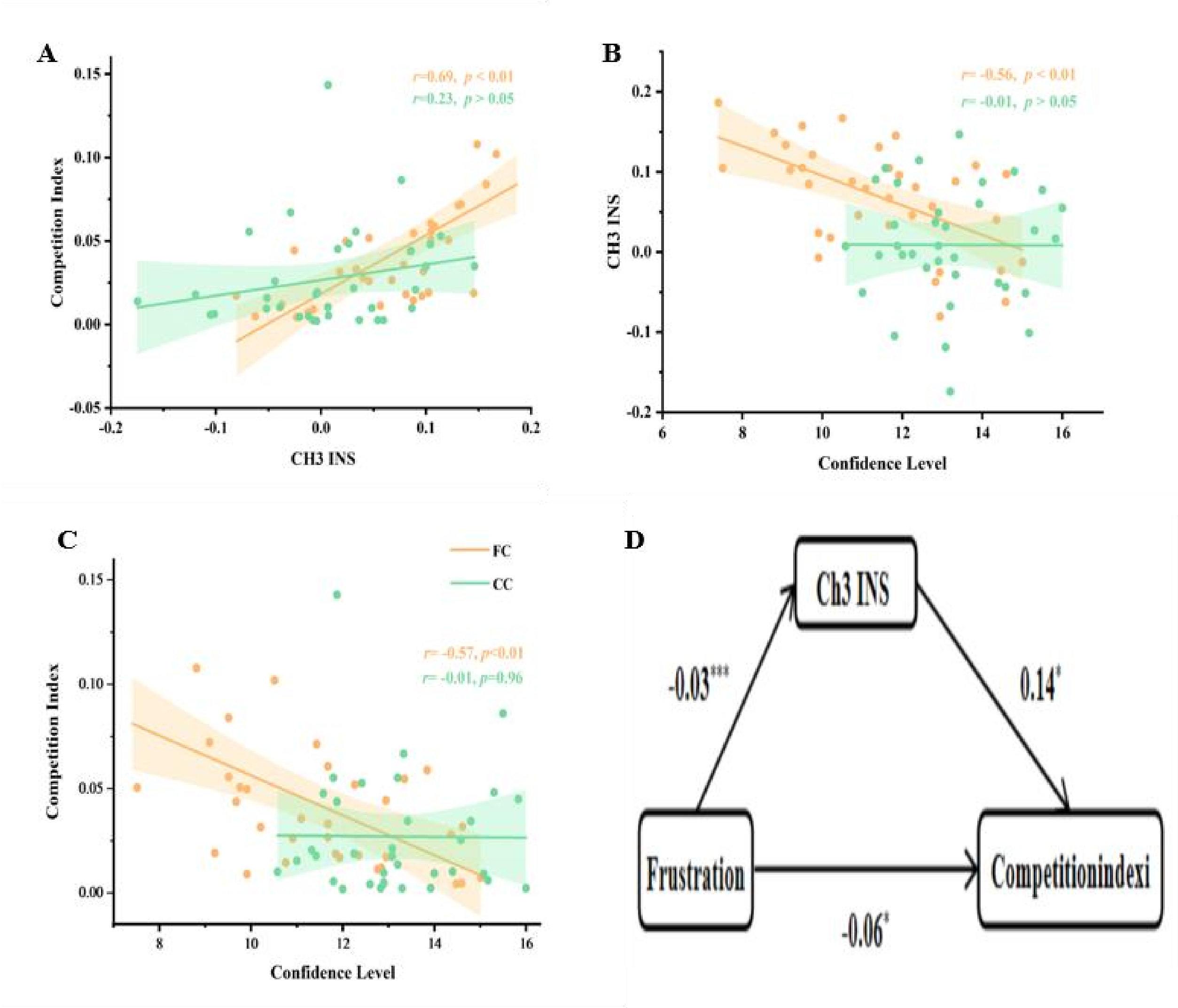

Fg. 6. Relationships between INS, psychological states, and competitive behavior. (A) Correlation between CH3 INS and competition index. Scatter plots show a significant positive correlation between task-related INS in CH3 and the competition index for FC, while no significant relationship was found for CC. (B) Correlation between CH3 INS and confidence level. In FC, higher INS in CH3 was significantly associated with lower subjective confidence levels. (C) Correlation between competition index and confidence level. A significant negative correlation was observed between the competition index and confidence levels in FC. (D) Mediation model. Path analysis reveals that the INS in CH3 significantly mediates the relationship between the frustration experience and the subsequent competition index. Standardized coefficients are shown on the paths, with asterisks indicating levels of statistical significance. *$p < 0.05$, ***$p < 0.001$. Shaded areas represent the 95% confidence interval.

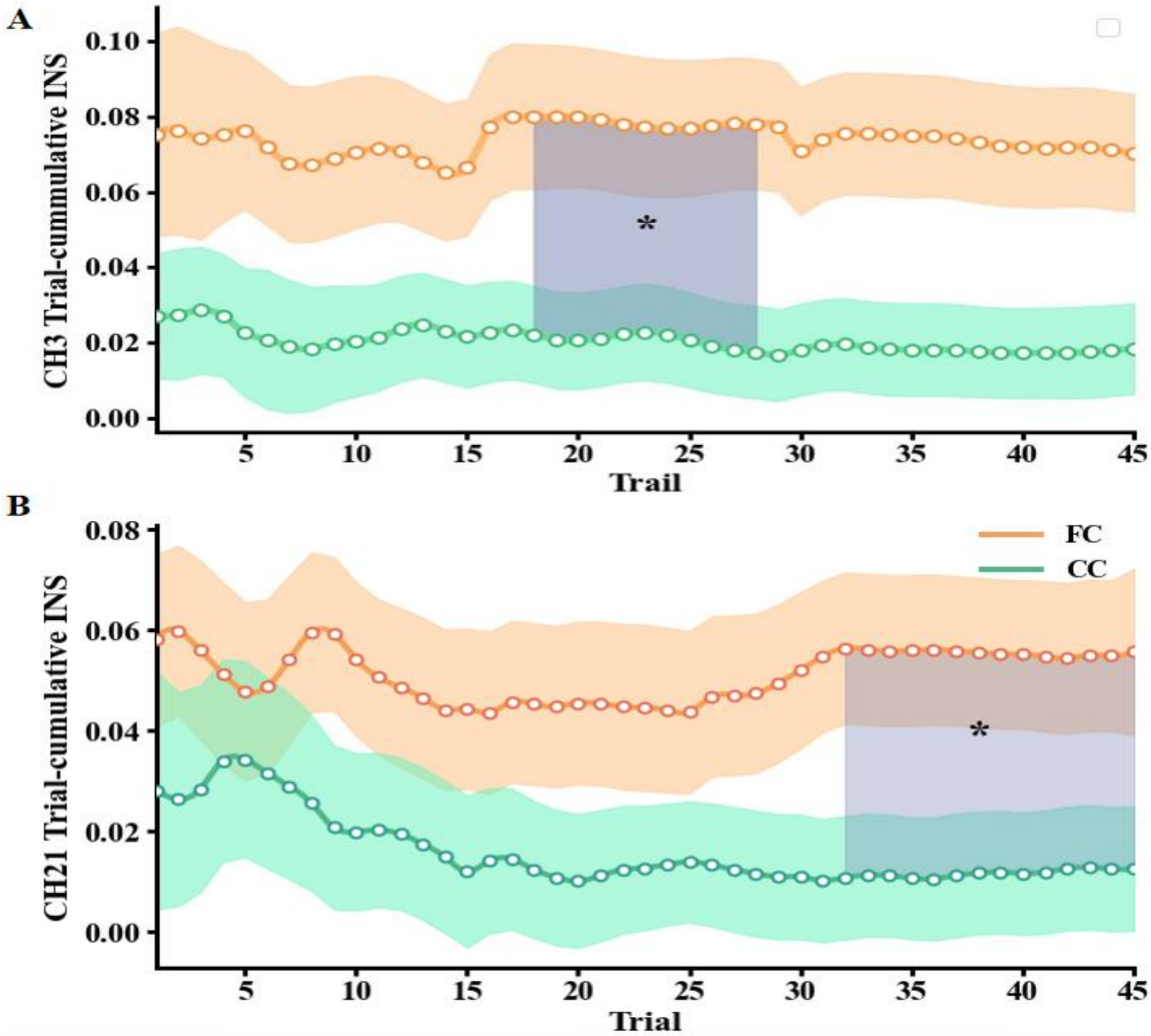

Fig. 7. Trial-accumulative INS dynamics analysis. (A) Cumulative INS in CH3. This plot illustrates the continuous accumulation of INS across 45 trials. A stable and significant difference between FC and CC, specifically between the 16th and 29th trials (indicated by the gray shaded rectangle). (B) Cumulative INS in CH21. Significant differences in CH21 emerged later in the task, starting from the 32nd trial and remaining consistent thereafter. Notes: Shaded bands around the solid lines represent the standard error of the mean (SEM). Gray shaded rectangles with asterisks (*) indicate stable significant windows ($p<0.05$).

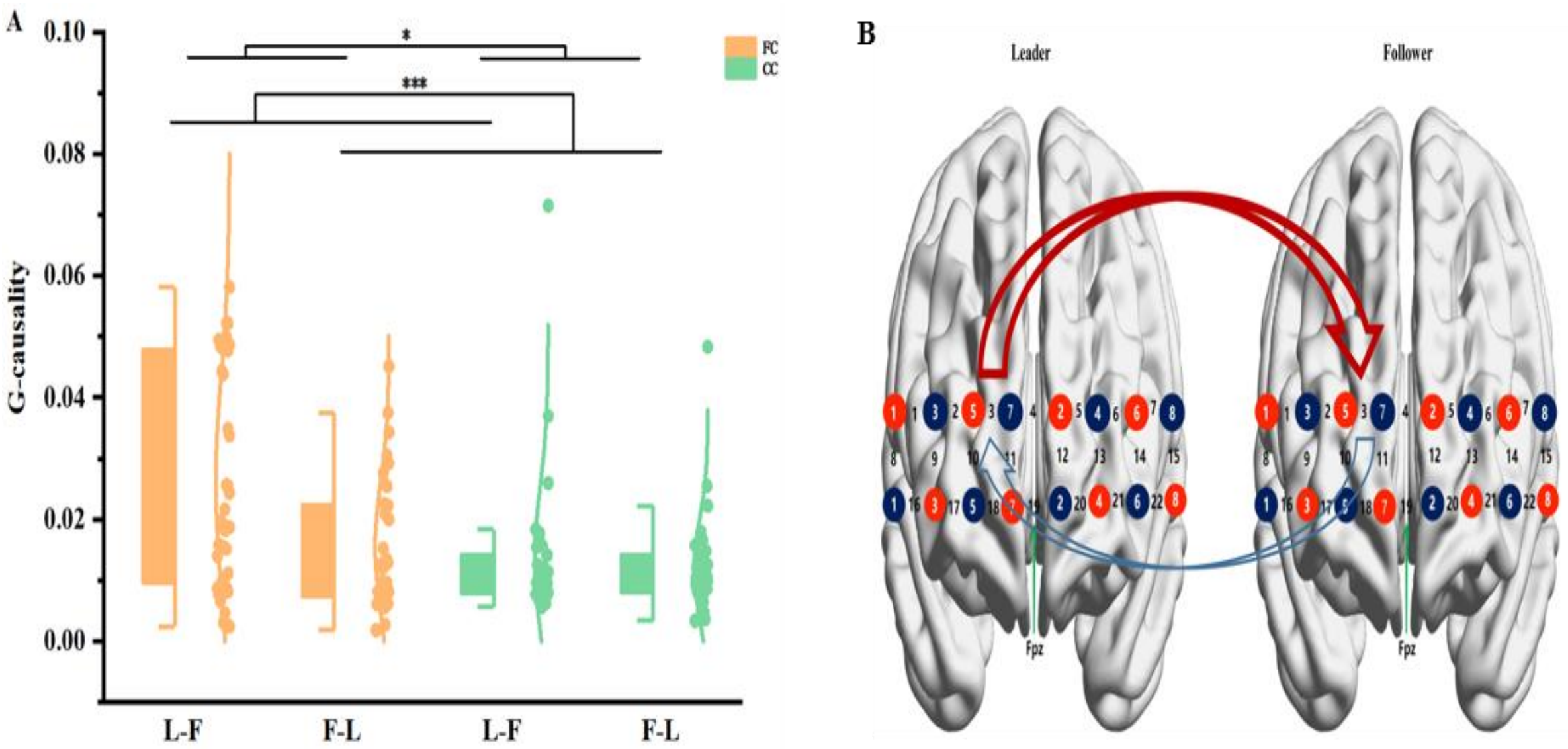

Fig. 8. Granger causality (G-causality) and coupling directionality. (A) Comparison of G-causality values. In FC, the G-causality from leader to follower (L-F) was significantly greater than from follower to leader (F-L), whereas no such directional difference was observed in CC. (B) Visualization of effective connectivity. The brain diagrams illustrate the direction and strength of information flow between the leader and the follower. The thicker red arrow represents the dominant influence from the leader to the follower in FC. *$p < 0.05$, ***$p < 0.001$. L-F = leader-to-follower; F-L = follower-to-leader.

COMMENT4-2: Secondly, portions of the writing are quite disjointed and incoherent. The introduction in particular repeats itself a lot and not flow neatly between points. For example on the bottom of page 4, the conflicting results on how frustration impacts competitiveness is discussed, then the effect of group frustration during protests is considered, and then on page 6 the effect of stressors on competitive behaviour is revisited.

RESPONSE: We are grateful for your insightful critique regarding the coherence and logical structure of the Introduction. We fully agree that the previous version contained redundancies and disjointed transitions. In accordance with your suggestions, we have thoroughly restructured the Introduction to ensure a "neat flow" and integrated the latest standing of the field as requested by the editor. The modifications are summarized as follows:

We have re-engineered the argumentative chain of the Introduction. The previously fragmented discussions regarding the "conflicting results of frustration"

and "general stressor effects" (formerly on pages 4 and 6) have been consolidated into a single, cohesive framework. Stressors are now treated as background evidence that directly supports our core hypothesis: how frustration, as a potent stressor, attenuates competition.

Following your specific advice, we removed extraneous content, such as the discussion on group frustration during protests.

We have dedicated substantial effort to consolidating the current standing of the field. The revised logical progression of the Introduction is as follows:

Paragraph 1 (Research Entry Point): Establishing frustration as a critical variable influencing interpersonal competition and defining the research background.

Paragraph 2 (Behavioral Hypothesis): Addressing conflicting findings in prior research and formulating the behavioral hypothesis that "frustration attenuates competitive behavior."

Paragraph 3 (Neural Hypothesis): Proposing the neural-level hypothesis regarding the enhancement of interpersonal neural synchronization (INS).

Paragraph 4 (Rationale for New Methodology): Justifying the necessity of transitioning from static to dynamic analysis based on the multi-stage dynamic mitigation characteristics of frustration.

Paragraph 5 (Roles and Directionality): Introducing "leader-follower" role differentiation and elucidating the scientific significance of utilizing Granger Causality Analysis (GCA) to detect directional neural interactions for analyzing asymmetrical interaction structures.

Paragraphs 6-7 (Experiment and Hypotheses): Summarizing the experimental protocol and presenting systematic hypotheses.

Please see the details below(see revised manuscript on page 4-8)：

Within the intricate social interaction network, interpersonal competition, as a fundamental mode of social interaction, serves as a crucial driving force for personal growth and societal advancement, with its internal mechanisms and external manifestations being prominent research topics in psychology (Tor & Garcia, 2023).

Traditional perspectives often characterize competition as a zero-sum game, wherein clashes among individuals or groups revolve around vying for finite resources, frequently accompanied by heightened stress, discord, and at times, animosity (Decety et al., 2004; Hsee et al., 2023). With the advances in social psychology, researchers have increasingly focused on the complexity of interpersonal competitive behavior, particularly examining how various factors influence individuals' competitive strategies (Garcia et al., 2013). Among these factors, although the potential impact of frustration as a significant contributor to interpersonal competition is evident, it has yet to garner widespread attention (Yu et al., 2016). It pervades all aspects of daily life and is considered a barrier to the realization of anticipated satisfaction (Dollard et al., 2013). Frustration not only triggers negative emotional responses, such as anxiety and anger (Yuan et al., 2015), but also alters an individual's mental state and behavioral patterns, ultimately affecting their performance in social interactions (Gallucci et al., 2020; Papini et al., 2022). Hence, it holds profound significance to unveil the patterns of human interpersonal competitive responses after frustrating situations and their underlying neural mechanisms.

Previous research on the relationship between frustration and competitive behavior has yielded contradictory conclusions. On one hand, some scholars posit that frustration can activate an individual's psychological resilience mechanisms, motivating individuals to exhibit heightened competitive drive and greater effort to overcome obstacles (Veltkamp et al., 2009; Radel et al., 2011). On the other hand, research suggests that frustration primarily acts as a potent psychological stressor, triggering psychological defense mechanisms (Papini et al., 2022;Vansteenkiste & Ryan, 2013). This discrepancy may stem from the nature of the task paradigm and the degree of psychological resource depletion: when frustration is perceived as a surmountable challenge, it may trigger resilience; however, in more complex interactive settings, frustration often significantly constricts cognitive bandwidth. According to the Theory of Cognitive Scarcity, this increased psychological strain depletes the cognitive resources required to execute sophisticated competitive

strategies (Waterschoot et al., 2020; De Bruijn et al., 2022). In such contexts, individuals tend to prioritize psychological equilibrium and self-preservation mechanisms over competitive victory, thereby exhibiting an avoidance of competitive scenarios (Liu et al., 2024). Therefore, considering the complexity of social interaction, we hypothesize that individuals experiencing frustration will exhibit diminished willingness in subsequent interpersonal competitive tasks.

Hyperscanning technology provides a critical perspective for uncovering the neural mechanisms underlying changes in interpersonal competitive behavior. Previous fNIRS-based hyperscanning studies have shown that in typical competitive interaction contexts, competing partners exhibit significant interpersonal neural synchronization (INS) in the right medial prefrontal cortex (mPFC). This synchronization is thought to reflect a strategic game process based on mutual prediction between interacting individuals (Liu et al., 2015). However, when psychological stressors intervene, distinct relationship patterns may emerge between interpersonal competitive behavior and neural synchronization. For example, a study involving young women found that acute psychosocial stress significantly suppressed overt competitive behavior, while a significant increase in INS was observed in the right dorsolateral prefrontal cortex (dlPFC) (Zhang et al., 2021). Similarly, another study showed that when individuals faced a mortality threat, although their competitive behavior decreased, neural synchronization between interacting individuals was enhanced (Zhou et al., 2022). Therefore, under challenging conditions, individuals may trigger a compensatory neural coupling mechanism. Specifically, psychological stressors may weaken competitive behavior between individuals, while interacting partners instead exhibit higher INS (Zhou et al., 2022). In such cases, the increased INS may reflect a highly integrated but less efficient interaction state, in which individuals need to invest more neural resources to maintain the interaction process, yet this integration does not necessarily correspond to higher behavioral efficiency.

However, despite current advances in fNIRS hyperscanning technology, the

assessment of INS remains limited by an implicit assumption—namely, that neural activity exhibits temporal stationarity throughout the measurement period (Li et al., 2021). Most existing studies tend to average data across the entire task duration to evaluate INS (Liang et al., 2022; Wang et al., 2022). In contrast, interpersonal competition is essentially a highly dynamic social interaction process involving complex, real-time adjustments of emotional, cognitive, and social strategies (Hamilton, 2021; Jiang et al., 2021). Furthermore, from a hyperscanning perspective, the interaction patterns between two participants after jointly experiencing frustration may exhibit dynamic characteristics that are distinct from those in single-person contexts. Research has indicated that jointly experiencing frustration not only influences individual emotional regulation through social referencing (Miao et al., 2021; Decety & Meyer, 2008) but also prompts participants to gradually adjust their goal expectations and motivational structures (Robinson & Moeller, 2014; Vansteenkiste & Ryan, 2013). Such shared experiences may trigger an adaptive psychological adjustment process: as frustration persists, individuals may gradually shift from their initial competitive focus toward maintaining psychological equilibrium, adopting more conservative behavioral strategies (Yuan et al., 2015). Therefore, the impact of frustration on interpersonal competition is not a monolithic instantaneous effect, but rather a dynamic mitigation process encompassing multi-stage changes (Dugré & Potvin, 2023). This perspective, focusing on ecological validity and temporal dynamics, aligns closely with recent academic calls to evaluate social interaction behavior in real-world contexts (Capotorto et al., 2024; Ronca et al., 2025). In summary, to more accurately and comprehensively understand the dynamic neural synchronization mechanisms during interpersonal competition under frustration, the present study employs a more refined approach to explore the dynamic fluctuations of INS throughout the competitive task.

Notably, in previous research on the neural synchronization mechanisms of interpersonal competition, two participants competing are usually considered equal and symmetrical (Cui et al., 2012), while relatively little attention has been paid to

role asymmetry, which may overlook the role differences that prevail in the real world (Decety et al., 2004; Liang et al., 2022). Indeed, research on leadership has found that leader-follower relationships spontaneously arise between participants in leaderless group discussions, even when they do not differ in natural or social status (Jiang et al., 2021). In addition, when faced with a decision when the group suffers a frustration, the greater the chance that the member who makes makes the final decision and is responsible for the outcome tends to be the leader among themselves (Liu., & Zhang., 2023; van Tuin et al., 2020), and thus interpersonal neural computation with role symmetry alone may mask some important information. In the present study, relative role differences (leader or follower) between individuals were assessed during the frustration task based on the dyads' performance of interactions during the frustration task, which could enable us to explore more comprehensively the patterns of interactions in the context of interpersonal competition for asymmetric roles. Granger Causality Analysis (GCA) was able to provide neurobiological cues for the present study as to which individual more actively drives the other (Pan et al., 2017). We therefore used GCA to further examine the coupled directionality of information flow between roles during competition.

In summary, this study will explore the dynamic effects of frustration on interpersonal competition using fNIRS hyperscanning. During the experiment, two participants cooperate to complete an adapted frustration-inducing task (Yuan et al., 2015). We collected corresponding emotional and psychological indicators via the Positive and Negative Affect Scale (PANAS) (Watson et al., 1988) and confidence level to verify whether the induction was successful. In addition, in the frustration task, we differentiated relative role differences by setting questions to assess participants' performance in the frustration task and thus. The classic keystroke task was used to assess interpersonal competition performance (Cui et al., 2012). During the interpersonal competition task, the fNIRS will simultaneously record the prefrontal region (PFC) of both subjects. This region plays an important role in competitive interactions (Zhang et al., 2023). Studies have shown that the PFC is associated with basic cognitive abilities such as attention (Benchenane et al., 2011)

and working memory (Braver et al., 1997), as well as with social cognitive abilities such as cognitive control (Sun et al., 2016), which are important in rapid key-pressing tasks. In addition, a significant increase in INS has been observed in PFC in several interpersonal competitive hyperscan studies (Zhang et al., 2023; Liu et al., 2015;). Therefore, we used the PFC as regions of interest (ROIs).

We hypothesized that: i) INS would be increased in the frustration condition(FC) relative to the control condition(CC). ii) Frustration dynamically reduces interpersonal competition across temporal phases. Dynamic INS in PFC is strongly correlated with interpersonal competition behaviors at the corresponding time points. iii) The leader-follower dynamic during interpersonal rivalry interactions is bidirectional, with the leader eliciting more neural activity in the follower.

COMMENT4-3： A similar problem occurs in the methods where the preprocessing of the fNIRS data is explained multiple times in different subsections.

RESPONSE: We appreciate your accurate observation regarding the redundancy in the Methods section. We have thoroughly reviewed the manuscript and identified several instances where preprocessing steps were unnecessarily repeated.To address this, we have streamlined the Methods section as follows:We have consolidated all data cleaning steps into a single, unified subsection: "2.4.3 fNIRS Data Preprocessing." In the previous version, Section 2.4.7 redundantly described PCA correction and z-score standardization. We have removed these descriptions, ensuring that the GCA section focuses solely on the causal modeling itself.

Please see the details below(see revised manuscript on page 14-15)：

2.4.3 fNIRS data analysis

Preprocessing

The fNIRS data underwent preprocessing through the Homer2 toolbox and a customized MATLAB-based script in MATLAB 2021a (Mathworks Inc., Natick, MA, USA). Initially, the raw light intensity signal was converted into optical density (OD).

Then, its quality was visually assessed according to the criterion of detector saturation or insufficient optical coupling (i.e., the absence of a heartbeat frequency of ~ 1 Hz in the signal's power spectrum). Any channels that failed to meet the criterion were deleted. Ultimately, 98.8% of the channels were retained for further analysis. The channels exhibiting poor signal quality were replaced with the average values of neighboring channels. If more than one-third of the channels exhibited poor quality, the dyad was excluded (Lu et al., 2020; Zhao et al., 2022). Second, the principal component analysis (PCA) was subsequently employed to eliminate signal artifacts resulting from spontaneous blood flow oscillations or other systematic factors (Zhang et al., 2016). A conservative variance threshold of 80% was applied to remove noise. Correlation-based signal improvement (CBSI) was utilized to mitigate head motion artifacts (Zhang et al., 2005). In line with previous studies, the data were neither filtered nor detrended for task-related INS analyses (Cui et al., 2012; Cheng et al., 2015). Finally, the optical density data for each channel were transformed into concentrations of oxyhemoglobin (HbO), deoxyhemoglobin (Hbr), and total hemoglobin (Hbt) using the modified Beer-Lambert law. Given that HbO signaling is more sensitive to changes in cerebral blood flow (Hoshi, 2007) and has been extensively utilized in fNIRS hyperscanning studies (Pan et al., 2017; Li et al., 2022), we focused on analyzing HbO signals.

COMMENT4-4： Finally, while I appreciate that there is conflicting results in the literature (motivating this work), more effort could be done to try to consolidate the current standing of the field in the introduction. For example, on the bottom of page 4 it is mentioned in quick succession that frustration can lead to enhanced competitive behaviour, an avoidance of competitive scenarios and an attempt to preserve self esteem, with relatively little discussion on why these conflicting results might occur.

RESPONSE: We sincerely thank you for the suggestion to further consolidate the current standing of the field. We agree that providing a theoretical explanation for the conflicting findings is essential for a robust research framework.

In the revised Introduction (Paragraph 2), we have added a dedicated theoretical

synthesis to reconcile the discrepancies in the literature. We propose that the divergence between resilience-based "promotion" and defense-based "avoidance" is primarily contingent upon the nature of the task paradigm and the degree of psychological resource depletion. Specifically, we have integrated the Theory of Cognitive Scarcity to explain that while frustration may act as a surmountable challenge in simpler contexts, it serves as a debilitating stressor in more complex interactive settings. In our study's dyadic context, frustration constricts "cognitive bandwidth" and shifts the individuals' priority toward self-preservation and psychological equilibrium rather than competitive victory.

Please see the details below(see revised manuscript on page 4-5):

Previous research on the relationship between frustration and competitive behavior has yielded contradictory conclusions. On one hand, some scholars posit that frustration can activate an individual′s psychological resilience mechanisms, motivating individuals to exhibit heightened competitive drive and greater effort to overcome obstacles (Veltkamp et al., 2009; Radel et al., 2011). On the other hand, research suggests that frustration primarily acts as a potent psychological stressor, triggering psychological defense mechanisms (Vansteenkiste & Ryan, 2013). This discrepancy may stem from the nature of the task paradigm and the degree of psychological resource depletion: when frustration is perceived as a surmountable challenge, it may trigger resilience; however, in more complex interactive settings, frustration often significantly constricts cognitive bandwidth. According to the Theory of Cognitive Scarcity, this increased psychological strain depletes the cognitive resources required to execute sophisticated competitive strategies (Waterschoot et al., 2020; Sheldon & Gunz, 2009). In such contexts, individuals tend to prioritize psychological equilibrium and self-preservation mechanisms over competitive victory, thereby exhibiting an avoidance of competitive scenarios (Liu et al., 2024; Paula & Campbell, 2002). Therefore, considering the complexity of social interaction, we hypothesize that individuals experiencing frustration will exhibit diminished willingness in subsequent interpersonal competitive tasks.

COMMENT4-5： I will also mention that a large portion of page 16 is missing. I do not know why this occurred but clearly this needs to be addressed.

RESPONSE: We sincerely apologize for the technical issue regarding the missing content on page 16. This error likely occurred during the PDF conversion or the initial submission system's file processing.

We have carefully reviewed the entire revised manuscript and the final generated PDF to ensure that all pages, sections, and paragraphs are complete and correctly displayed. The missing text has been fully restored in this revised version. We have also performed a thorough check of the document's formatting to prevent any recurrence of such issues. We thank you for bringing this to our attention.

COMMENT 5: Significance: Overall I think the quality in the design of the paradigm lends this work a high degree of significance, making communications biology an appropriate journal for the work. I also appreciate the limitations section which demonstrates that future work could clearly build on the approach described in this work - adding to its significance. I do think that some discussion could be added in cases where the results of this work contradict prior results - once again looking at Veltkamp et al (2009) and Radel et al (2011,2014). Why might this work find completely different results from these works? However, due to the depth of validation in the paradigm and precision in the metrics and results I believe the results of this work are reproducible, with the main limitation to the reproducibility coming from the clarity in the description of the methods. Specifically I think Section 2.2.1 could use more clarity in how the points system works for this game, how feedback is given and how confidence levels were determined. While I think this task does induce frustration, the section is too vague to be completely reproducible.

COMMENT5-1： Overall I think the quality in the design of the paradigm lends this work a high degree of significance, making communications biology an appropriate journal for the work. I also appreciate the limitations section which

demonstrates that future work could clearly build on the approach described in this work - adding to its significance.

RESPONSE: We are sincerely grateful for your positive assessment of our paradigm design and its significance. We are pleased that you found the work appropriate for Communications Biology.

COMMENT5-2： I do think that some discussion could be added in cases where the results of this work contradict prior results - once again looking at Veltkamp et al (2009) and Radel et al (2011,2014). Why might this work find completely different results from these works?

RESPONSE: We sincerely thank you for this insightful comment. We fully agree that it is essential to conduct an in-depth discussion on the discrepancy between our findings and the studies by Veltkamp et al. (2009) and Radel et al. (2011, 2014). This helps clarify the boundary conditions of the present study.

We have added a dedicated paragraph in the Discussion section of the revised manuscript to explain this difference from the following three perspectives:First, the difference in task paradigms (individual tasks vs. social interaction). The studies by Veltkamp and Radel primarily utilized individual tasks. In such contexts, frustration is perceived as a challenge to personal competence, and individuals tend to restore damaged self-esteem by increasing effort. However, the present study employed a dyadic interaction paradigm under hyperscanning. In complex social competition, individuals must cope not only with task frustration but also with the social pressure brought by their opponents. Such high-load interpersonal interaction rapidly depletes an individual's cognitive bandwidth, leading them to shift toward a behavioral pattern of mitigating competition.

Second, the dynamic evolution process of frustration. Previous research has focused more on the acute motivational arousal following frustration (i.e., a "temporary increase in drive"). In contrast, our study observed long-term dynamic changes across three experimental blocks. Results showed that the mitigation of competitive willingness occurred progressively. This suggests that under sustained

frustration pressure, an individual's psychological regulation mechanism undergoes a transition.

Third, the interpersonal cohesion generated by shared experiences of frustration. This is the most fundamental difference between the present study and individual task research. In dyadic interaction, the shared frustration experienced by both parties can act as an emotional bond to enhance interpersonal cohesion, thereby reaching a tacit understanding to mitigate competition.

Please see the details below(see revised manuscript on page 30):

Our findings diverge from the previous perspective that frustration serves as a motivational catalyst for mobilizing psychological resources (Veltkamp et al., 2009; Radel et al., 2011, 2014). As noted in the introduction, this discrepancy may highlight a key boundary condition of frustration-regulated behavior: the shift from an 'individual achievement' context to a 'social interaction' context. In isolated individual tasks, frustration is typically perceived as a challenge to personal competence, thereby inducing a compensatory mobilization of effort to restore self-efficacy (Radel et al., 2011). However, from a hyperscanning perspective on dyadic interaction, shared frustration acts as a potent psychological stressor that serves as an 'emotional bond,' mitigating competition by enhancing interpersonal cohesion and thereby achieving a dynamic balance of psychological resources within complex interactive environments.

COMMENT5-3： However, due to the depth of validation in the paradigm and precision in the metrics and results I believe the results of this work are reproducible, with the main limitation to the reproducibility coming from the clarity in the description of the methods. Specifically I think Section 2.2.1 could use more clarity in how the points system works for this game, how feedback is given and how confidence levels were determined. While I think this task does induce frustration, the section is too vague to be completely reproducible.

RESPONSE: We sincerely thank you for identifying the areas in Section 2.2.1

that required further elaboration to ensure full reproducibility. We agree that a detailed description of the task mechanics is essential. Accordingly, we have thoroughly revised Section 2.2.1 to provide a more transparent and precise protocol. The specific updates are summarized as follows:

1.Clarification of the Points System and Role Assignment: We have specified that a points system was employed solely to distinguish between the roles of "Leader" and "Follower" within each dyad. Participants earned 2 points for individual dominance in decision-making and 1 point each for joint determination. The individual with the higher cumulative score after 12 trials was designated as the "Leader."

2.Detailing the Feedback Mechanism: We have clarified that feedback was provided on a trial-by-trial basis immediately following the confidence rating. The feedback was displayed for 3,000 ms and was pre-programmed with an 83.3% failure rate (10 incorrect vs. 2 correct trials) to ensure robust frustration induction, regardless of actual performance.

3.Refinement of Confidence Level Assessment: We have added that participants rated their confidence in the accuracy of the input number on an untimed 7-point Likert scale (ranging from 1 = not at all confident to 7 = extremely confident).

4.Inclusion of Timing Parameters: We have added that each geometric image was presented for a maximum of 120 seconds, with an option for participants to press the spacebar to terminate the presentation early once a consensus was reached.

These additions ensure that the paradigm is now fully transparent and reproducible.

Please see the details below(see revised manuscript on page 9-10)：

The frustration-inducing task was adapted from the research of Yuan et al. (2015). To ensure the scientific validity of the task duration and difficulty, an independent sample of 24 dyads was recruited to pre-evaluate 20 candidate geometric images using an 8-point Likert scale (1 = particularly easy, 8 = especially difficult). Based on their evaluations, 10 medium-difficulty images (range: 3.55–5.13) and 2 low-difficulty images (range: 1.46–2.34) were ultimately selected as stimulus

materials for the formal task, which consisted of 12 trials. In each trial, complex geometric images were presented for a maximum duration of 120 seconds. Participants were permitted oral communication while remaining physically still to reach a consensus on the number of triangles. If a consensus was reached before the time limit, participants could press the spacebar to terminate the image presentation and proceed immediately to the answer entry phase. Following the image presentation, the participant seated on the right entered the agreed-upon answer. Subsequently, to distinguish between roles within the dyad, participants were prompted to identify the decision-making process: (i) dominance by the left-side participant (2 points), (ii) joint determination (1 point each), or (iii) dominance by the right-side participant (2 points). After 12 trials, the individual with the higher cumulative score was defined as the "leader," and the other as the "follower." Immediately after role evaluation, participants assessed their confidence in the accuracy of the input number using an untimed 7-point Likert scale (1 = not at all confident, 7 = extremely confident), with the left-side participant responding first. Finally, a feedback screen was presented for 3,000 ms. To consistently induce frustration, feedback was manipulated as "Incorrect Answer!" (totaling 10 trials, with an 83.3% failure rate) regardless of the actual response, and "Correct Answer!" was only displayed during the 2nd and 5th trials (low-difficulty images). Finally, a blank screen was presented during the inter-trial intervals.

Responses to Reviewer 3

COMMENT 1: In the Methods section (paragraph 2.3), the authors state that they used the NIRS_SPM package in MATLAB to further analyse the spatial anatomical location. Could the authors elaborate on this? Specifically, did you register all participants to MNI space? Did you use a specific atlas to determine whether one or more channels were localised in a particular brain region? Please clarify.

RESPONSE: We appreciate your request for clarification on our spatial localization

procedure. We would like to confirm that all participants in our study underwent individual spatial registration to the MNI space. The spatial registration and anatomical labeling were conducted as follows: the raw 3D coordinates for each participant, collected via FASTRAK, were imported into the NIRS_SPM package. Using the "Spatial Registration" module, an anchor-based transformation was applied to map each individual's unique head geometry onto the standard MNI (Montreal Neurological Institute) template. Anatomical labeling was performed utilizing the Anatomical Estimation function within NIRS_SPM, which is based on the Brodmann atlas and the LPBA40 (Loni Probabilistic Brain Atlas). Detailed MNI coordinates and anatomical information for all 22 channels are now provided in the Supplementary Material.

Please see the details below(see revised manuscript on page 9-10):

2.3 fNIRS data acquisition

Two identical fNIRS systems (LIGHTNIRS; Shimadzu Co., Japan) were utilized to record the brain signals of two participants in a dyad during the experiment. This fNIRS system has a sampling rate of 13.3 Hz and three near-infrared wavelengths (780 nm, 805 nm, and 830 nm). By utilizing an external trigger system to simultaneously send marking signals to both acquisition units, we ensured millisecond-level temporal alignment at the onset of the experiment and at each trial transition. For each participant, an 8×2 probe patch was positioned to cover the bilateral prefrontal cortex, resulting in a total of 22 recording channels (CHs) (Fig. 2C). Each patch was equipped with 8 emitters and 8 detectors. The distance between emitters and detectors was approximately 3 cm. The placement of the emitter and detector followed the 10–20 system (Okamoto et al., 2004). The lower row of optodes was positioned along the Fp1-Fpz-Fp2 line. A 3D magnetic space digitizer (FASTRAK; Polhemus, USA) was employed to assess the anatomical positions of optodes and channels for each participant, using NZ, CZ, AL, and AR as reference points (Singh et al., 2005; Tsuzuki et al., 2007). The spatial anatomical locations were further analyzed using the NIRS_SPM MATLAB package (Ye et al., 2009), whereby

individual coordinates were registered to the MNI space. Anatomical labels were determined based on the Brodmann and LPBA40 atlases. Detailed MNI information for all channels is provided in the Supplementary Material .

COMMENT 2: The authors selected the frequency of interest for their analysis by performing t-tests comparing the task to the baseline over the full frequency range. There is a serious risk of double-dipping here, as the frequencies that already showed significant differences between task and baseline might not be independent of the subsequent FC versus CC comparison. This issue was recently raised in a review by Hakim et al. (2023; https://doi.org/10.1016/j.neuroimage.2023.120354), in which the authors warned about the risk of double-dipping and the issues associated with such a method. Could you provide further details on how you controlled for this potential double-dipping when using the FOI to compare FC and CC? For example, did you employ any cross-validation or use independent data for frequency selection? Additionally, the authors state that the results are in line with previous studies; however, the resulting frequency range ([0.12–0.21 Hz]) falls within the range reported by the cited papers but is not identical to the [0.08–0.3 Hz] range. Could you provide further information on this discrepancy? Is it because the selected range coincides with the durations of the task set-up?

RESPONSE: Thank you very much for your professional suggestions. We have carefully studied the review published by Hakim et al. (2023) in NeuroImage, which has been highly instrumental in enhancing the methodological rigor of our study. Regarding the selection of the frequency of interest (FOI), we provide the following detailed explanation:

Generally, there are three approaches for choosing the FOI in fNIRS hyperscanning: (1) The duration of the trials setting with Wavelet coherence plot between time courses from participants within dyad (Cui et al., 2012; Hu et al., 2017; Osaka et al., 2015; Tang et al., 2016; Zhang et al., 2021); (2) Selecting one subset from the remaining frequency bands after excluding the high and low-frequency physiological noise (i.e., respiration (around 0.2-0.3Hz), cardiac pulsation (around

1Hz)) and high-frequency head movements (Jiang et al., 2012; Jiang et al., 2015; Dai et al., 2018). For example, the band range of 0.01-0.4Hz was chosen in a face-to-face vocal communicating; (3) Based on the method (2), after exclusion of the high and low-frequency noise, the statistical examination is conducted between the experimental conditions on the coherence value of each channel along with the full period range. The approach identified the frequency ranges that are specifically associated with the cognitive process of interest (Zheng et al., 2018; Liu et al., 2019; Pan et al., 2020; Long et al., 2021; Nguyen et al., 2021; Lu et al, 2020). As current fNIRS hyperscanning research requires greater rigor in frequency selection, we utilized a combination of Approach (1) and Approach (3).

To directly address your concern regarding the potential risk of double-dipping, we have performed a supplementary analysis using Leave-One-Subject-Out Cross-Validation (LOSO-CV). Detailed information can be found in Supplementary Analysis 1.

Furthermore, we sincerely apologize for the lack of precision in our manuscript regarding the statement that the selected FOI was consistent with previous research despite the discrepancy in the specific range. To ensure rigor, we have removed the relevant statements. Our original intention was to explain that the current frequency range was determined by integrating Approach (3) (statistical screening) and Approach (1) (task cycle). As you correctly pointed out, this frequency range indeed aligns closely with the duration of our task setup. In this study, the average duration of the competitive trials was approximately 5.5 seconds, which falls precisely within the identified 0.12–0.21 Hz frequency band.

Please see the details below(see revised manuscript on page 15-16, Supplementary Analysis 1)：

INS data analysis

Following preprocessing, the Wavelet Transform Coherence (WTC) method was applied on each channel to compute the INS of interacting individuals (Grinsted et al., 2004). In contrast to correlation methods, WTC is regarded as more appropriate due

to its normalization of the signal amplitude in accordance with each time window, rendering it less susceptible to motion-induced transient spikes (Nozawa et al., 2016). Compared to traditional cross-correlation methods, WTC enables the decomposition of signals in both the time and frequency domains simultaneously. Channel coherence was calculated utilizing the WTC package within a bespoke MATLAB script. The WTC of HbO time series i (m) and j (m) at wavelet scale s was determined according to the following formula:

$$\mathrm{WTC}(m,s) = \frac{\left|\langle s^{-1}W^{ij}(m,s)\rangle\right|^2}{\left|\langle s^{-1}W^{i}(m,s)\rangle\right|^2\left|\langle s^{-1}W^{j}(m,s)\rangle\right|^2}$$

where m, s, and W denote time, wavelet scale, and the continuous wavelet transform, respectively. **To identify the task-related frequency band while avoiding the issue of double-dipping (Hakim et al., 2023), we employed a Leave-One-Subject-Out Cross-Validation (LOSO-CV) method. This approach integrates frequency selection with classification modeling to ensure the independence of the analysis process (detailed procedures are provided in Supplementary Analysis 1)**. The results showed increased INS for frequencies ranging between 0.12 and 0.21 Hz. This frequency band excludes low-frequency fluctuations (below 0.01 Hz) and high-frequency physiological signals, such as cardiac pulsation (~ 1 Hz) and respiration (~ 0.3 Hz) (Pierro et al., 2014; Yücel et al., 2021). In this FOI, each channel's INS was computed individually for baseline and interpersonal interaction tasks, and then the latter was subtracted from the former to obtain the task-related INS (in comparison to the baseline). Following the acquisition of the INS values for each dyad and channel, single-sample t-tests were employed to examine whether any of the channels showed a significant enhancement of INS during cooperation, aiming to mitigate the risk of false findings and enhance the robustness of the results. The channels of interest in this investigation comprised all those that exhibited significant INS. The task-specific channels were then subjected to independent samples t-test analyse and two-way analysis of variance to assess whether there existed a difference between the FC and CC.

Supplementary Analysis 1

Leave-One-Subject-Out Cross-Validation for FOI Selection

To address potential concerns regarding double-dipping in the frequency band selection process (Hakim et al., 2023), we implemented a Leave-One-Subject-Out Cross-Validation (LOSO-CV) strategy. This approach integrated frequency-of-interest (FOI) identification with classification modeling to ensure the independence of the statistical inference and validation phases.

In each iteration of the cross-validation, data from one dyad was held out as the test set. Using the remaining N−1 dyads, paired-samples t-tests were conducted across the full frequency range (0.01–1 Hz) to compare neural activity between the task and baseline periods. Frequency bins that exhibited significant differences after FDR correction ($p<0.05$) were identified as the FOI for that specific fold. Subsequently, the average INS values across all channels within this dynamically selected FOI were extracted for the excluded dyad. Based on the INS features, a Logistic Regression (LR) model was trained on the N−1 dyads to distinguish between the frustration condition (FC = 1) and the control condition (CC = 0). The trained model was then utilized to predict the category of the held-out dyad. This procedure was repeated N times until every dyad had served as the test set. Classification performance was evaluated using prediction accuracy and the Area Under the ROC Curve (AUC), with a 1000-iteration permutation test performed to determine if the performance significantly exceeded chance levels.

Furthermore, to evaluate the stability of the frequency selection across folds, we generated binary heatmaps of the selected frequency bins for each iteration and calculated a frequency selection density curve. This allowed for a quantitative comparison between the cross-validated frequency bins and our initially identified FOI (0.12–0.21 Hz), ensuring the robustness and consistency of our results.

The results demonstrated that: (1) the FOI selected in each fold was consistently centered on the 0.12–0.21 Hz frequency band; (2) the classification accuracy and AUC of the Logistic Regression model across all folds were significantly higher than

chance levels (Accuracy = 0.771, Precision = 0.806, Recall = 0.714, F1-score = 0.758, ROC-AUC = 0.87); and (3) the permutation test confirmed the statistical significance of the model's performance. Collectively, these findings indicate that our FOI selection procedure did not introduce double-dipping bias, and the differences between the FC and CC conditions are robust and generalizable across participants.

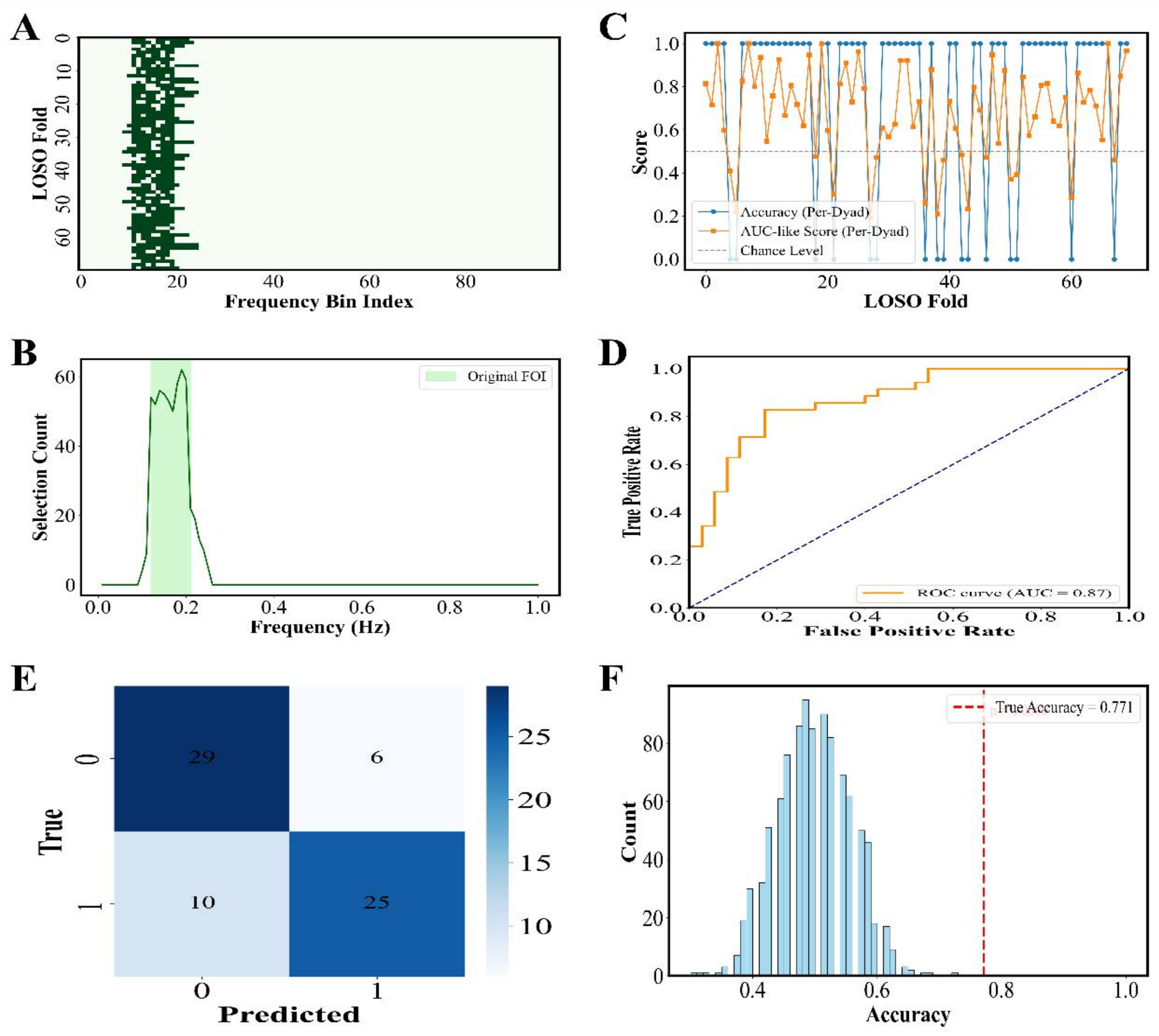

Figure S1. Leave-One-Subject-Out Cross-Validation (LOSO-CV) Analysis for FOI Selection and Model Performance. (A) Heatmap of the selected frequency bands across the 70-fold cross-validation. Each row represents one LOSO iteration, and green squares indicate the frequency bins selected as the frequency-of-interest (FOI) in that fold. (B) Frequency selection density plot. The green curve shows the frequency with which each frequency bin was selected; the light green shaded area indicates the initially identified FOI interval (0.12 –

0.21 Hz). (C) Model performance metrics for each fold. The blue line represents accuracy and the orange line represents AUC; all predicted values were significantly higher than chance levels. (D) ROC curve of the LOSO-CV model, showing good classification capability (AUC = 0.87). (E) Confusion matrix. (F) Histogram of the permutation test with 1000 shuffled labels, where the red line represents the accuracy of the predictive model.

Supplementary Reference:

Cui, X., Bryant, D. M., & Reiss, A. L. (2012). NIRS-based hyperscanning reveals increased interpersonal coherence in superior frontal cortex during cooperation. *Neuroimage, 59*(3), 2430-2437.

Dai, B., Chen, C., Long, Y., Zheng, L., Zhao, H., Bai, X., ... & Lu, C. (2018). Neural mechanisms for selectively tuning in to the target speaker in a naturalistic noisy situation. *Nature communications, 9*(1), 1-12.

Hu, Y., Hu, Y., Li, X., Pan, Y., & Cheng, X. (2017). Brain-to-brain synchronization across two persons predicts mutual prosociality. *Soc Cogn Affect Neurosci, 12*(12), 1835-1844.

Jiang, J., Chen, C., Dai, B., Shi, G., Ding, G., Liu, L., & Lu, C. (2015). Leader emergence through interpersonal neural synchronization. *Proceedings of the National Academy of Sciences, 112*(14), 4274-4279.

Jiang, J., Dai, B., Peng, D., Zhu, C., Liu, L., & Lu, C. (2012). Neural synchronization during face-to-face communication. *Journal of Neuroscience, 32*(45), 16064-16069.

Li, Z., Li, J., Hong, B., Nolte, G., Engel, A. K., & Zhang, D. (2021). Speaker–Listener Neural Coupling Reveals an Adaptive Mechanism for Speech Comprehension in a Noisy Environment. *Cerebral Cortex*.

Liu, J., Zhang, R., Geng, B., Zhang, T., Yuan, D., Otani, S., & Li, X. (2019). Interplay between prior knowledge and communication mode on teaching effectiveness: interpersonal neural synchronization as a neural marker. *NeuroImage, 193*, 93-102.

Long, Y., Zheng, L., Zhao, H., Zhou, S., Zhai, Y., & Lu, C. (2021). Interpersonal neural synchronization during interpersonal touch underlies affiliative pair bonding between romantic couples. *Cerebral Cortex, 31*(3), 1647-1659.

Lu, K., Yu, T., & Hao, N. (2020). Creating while taking turns, the choice to unlocking group creative potential. *NeuroImage, 219*, 117025.

Nguyen, T., Schleihauf, H., Kayhan, E., Matthes, D., Vrtička, P., & Hoehl, S. (2021). Neural synchrony in mother–child conversation: Exploring the role of conversation patterns. *Social Cognitive and Affective Neuroscience, 16*(1-2), 93-102.

Osaka, N., Minamoto, T., Yaoi, K., Azuma, M., Shimada, Y. M., & Osaka, M. (2015). How two brains make one synchronized mind in the inferior frontal cortex: fNIRS-based hyperscanning during cooperative singing. *Frontiers in psychology, 6,* 1811.

Pan, Y., Dikker, S., Goldstein, P., Zhu, Y., Yang, C., & Hu, Y. (2020). Instructor-learner brain coupling discriminates between instructional approaches and predicts learning. *NeuroImage, 211*, 116657.

Tang, H., Mai, X., Wang, S., Zhu, C., Krueger, F., & Liu, C. (2016). Interpersonal brain

synchronization in the right temporo-parietal junction during face-to-face economic exchange. *Soc Cogn Affect Neurosci, 11*(1), 23-32.

Zhang, R., Zhou, X., Feng, D., Yuan, D., Li, S., Lu, C., & Li, X. (2021). Effects of acute psychosocial stress on interpersonal cooperation and competition in young women. *Brain and Cognition, 151*, 105738.

Zheng, L., Chen, C., Liu, W., Long, Y., Zhao, H., Bai, X., ... & Lu, C. (2018). Enhancement of teaching outcome through neural prediction of the students' knowledge state. *Human Brain Mapping, 39*(7), 3046-3057.

COMMENT 3: In the Results section (paragraph 3.7), it is not clear whether the t-test results pertain to channel 3 or channel 21. Could you please clarify?

RESPONSE: We thank you for the reminder and apologize for the lack of clarity in Section 3.7. We would like to clarify that the one-sample t-test results reported in the first paragraph of Section 3.7 (used to verify whether the G-causalities were significantly greater than zero) were conducted on CH3 (localized in the FPC).

Please see the details below(see revised manuscript on page 27-28):

In CH3, G-causalities under the FC were significantly greater than zero in both directions: from leader to follower ($t(34) = 10.29$, $p < 0.001$) and from follower to leader ($t(34) = 17.80$, $p < 0.001$). Results in CC were similar to FC, with significant increases in G-causalities relative to zero in both directions: from leader to follower ($t(34) = 8.88$, $p < 0.001$) and from follower to leader ($t(34) = 12.26$, $p < 0.001$).

COMMENT 4: There are two citations in the Introduction (line 135: Hari et al., 2018; line 166: Hamilton, 2021) that are not present in the bibliography.

RESPONSE: We sincerely apologize for this oversight. The missing references for Hari et al. (2018) and Hamilton (2021) have now been added to the bibliography. Additionally, we have performed a thorough cross-check of all citations throughout the manuscript to ensure that every cited work is correctly listed in the References section.

COMMENT 5: In the Methods section, when discussing the CBSI, the citation is incorrect. The correct reference for the CBSI method is "Functional near infrared spectroscopy (NIRS) signal improvement based on negative correlation between oxygenated and deoxygenated haemoglobin dynamics."

RESPONSE: We thank you for pointing out this error. We apologize for the incorrect citation. The reference for the CBSI method has been corrected to the original paper: **Cui, X., Bray, S., & Reiss, A. L. (2010). Functional near infrared spectroscopy (NIRS) signal improvement based on negative correlation between oxygenated and deoxygenated hemoglobin dynamics. *Neuroimage*, *49*(4), 3039-3046.** We have updated this citation in the Methods section of the revised manuscript.

Manuscript ID: COMMSBIO-24-7780A

Title: Dynamic Interpersonal Neural Synchronization of Frustration Mitigated Interpersonal Competition：a fNIRS-based Hyperscanning Study

Revised title: (The manuscript title has been adjusted in accordance with the journal's specifications) ：Frustration reduces interpersonal competition through dynamic interpersonal neural synchronization in dyads

Dear Editor and Reviewers,

We sincerely thank the Editor and Reviewers for their time, careful evaluation, and constructive comments on our manuscript entitled “Frustration reduces interpersonal competition through dynamic neural synchronization in dyads” submitted to *Communications Biology*.

In this revised version, we have carefully addressed all remaining comments raised during the review process and made comprehensive improvements to enhance the clarity, rigor, and presentation of the manuscript. Specifically, we have:

1. systematically corrected typographical and formatting issues throughout the manuscript;
2. regenerated all figures at high resolution (600 dpi) and improved the clarity and readability of figure text, labels, and legends;
3. resolved a graphical artefact in Figure 7A introduced during figure export;
4. clarified the implementation details of the LOSO-CV and permutation procedures in Supplementary Note 1, explicitly confirming that each permutation reruns the full cross-validation pipeline, including foldwise FOI selection, thereby ensuring no data leakage.

In addition, we would like to respectfully inform the Editorial Office of an update to the first author’ s affiliation information and the funding information, and we kindly ask for the Editorial Office’ s consideration. The experimental work, data collection, and original submission of this study were completed while the first author was affiliated with North China University of Science and Technology. During the revision process, the first author began doctoral study at Shaanxi Normal University.

Accordingly, in this revised version, we have retained North China University of Science and Technology as the primary affiliation where the study was conducted and originally submitted, and have added Shaanxi Normal University as the first author’s current affiliation. We have also added the relevant funding information to the Acknowledgements/Funding section and updated the corresponding information in the submission system. We would be very grateful for the Editorial Office’s guidance if any further explanation, adjustment, or documentation is required.

All changes have been highlighted in the revised manuscript, and a detailed point-by-point response is provided below.

We thank you again for your time and consideration.

Sincerely,
Yingjie Liu

Reviewer 1: Many thanks for the revising work. All the issues were properly addressed.

Response:

We sincerely thank Reviewer 1 for their positive evaluation of our revised manuscript. We greatly appreciate their time and constructive feedback throughout the review process.

Reviewer 2：I have read the author responses in full and reread the full manuscript. My original comments stand that I feel the work has sufficient novelty and will likely shape future work. My original clarity concerns, which were my largest critiques of the original, have been improved on and I thank the authors for their efforts in revising the manuscript. I have no remaining large concerns. Just some minor points:

1. At the bottom of page 7 there is a sentence which ends "..frustration task and thus." which appears to be incomplete
2. The figure captions are improved but I think the figures themselves are still very low resolution and this is very clear in cases where there is text such as on Figure 1 and the legend of Figure 5
3. There is a typo in the caption of Figure 6 at "behaviour(n=35)"
4. Figure 7A seems to have a floating and empty legend square
5. Top of page 30 there is a typo at "consecutive trialS"

Response:

We sincerely thank Reviewer 2 for the thorough re-evaluation of our revised manuscript and for the constructive and highly encouraging feedback. We are pleased that the revisions have addressed the reviewer’ s previous concerns and improved the clarity and quality of the manuscript. We appreciate the additional minor comments, which have further helped us improve the manuscript.

Comment 1：At the bottom of page 7 there is a sentence which ends "..frustration task and thus." which appears to be incomplete.

Response:

We thank the reviewer for identifying this incomplete sentence. The unintended phrase "and thus" has been removed. The sentence has been revised as follows:

Original: "In addition, in the frustration task, we differentiated relative role differences by setting questions to assess participants' performance in the frustration task and thus."

Revised: "In addition, relative leader and follower roles were assigned based on participants' responses to the task questions and their performance during the frustration task." This change has been made in the revised manuscript, Page 7, Lines 18–20.

Comment 2: The figure captions are improved but I think the figures themselves are still very low resolution and this is very clear in cases where there is text such as on Figure 1 and the legend of Figure 5

Response:

We thank the reviewer for this valuable comment. In response, all figures have been regenerated and replaced with high-resolution versions (600 dpi) in accordance with the journal's specifications. We have also carefully reviewed the readability of all figure text, labels, and legends and made adjustments where necessary. We believe these revisions have substantially improved the clarity and overall presentation of the figures throughout the manuscript.

Comment 3：There is a typo in the caption of Figure 6 at "behaviour(n=35)"

Response:

We thank the reviewer for identifying this formatting issue in the Figure 6 caption. We have corrected the typographical error by revising "competitive behavior.(n = 35)" to "competitive behavior (n = 35)." to ensure proper formatting and consistency. The revised caption has been updated in the manuscript.

This change has been made in the revised manuscript, Page 37, Lines 18–19.

Figure 5. Relationships between INS, psychological states, and competitive behavior

(n = 35 dyads).

Comment 4：Figure 7A seems to have a floating and empty legend square

Response:

We thank the reviewer for pointing out this issue. The appearance of a floating empty legend box in Figure 7A was due to a graphical artefact introduced during figure export. We have now completely regenerated all figures following the journal’s formatting requirements, and the issue has been resolved in the revised version. The updated Figure 7A no longer contains this artefact.

Comment 5：Top of page 30 there is a typo at "consecutive trialS"

Response:

We thank the reviewer for identifying this typographical error. The word “trialS” has been corrected to “trials” in the revised manuscript.

Reviewer 3: The authors have addressed my previous questions appropriately. The manuscript is now much clearer, the Methods section is complete and easy to follow, and the Discussion is balanced and informative. I support publication of the manuscript in the *Communications Biology* Journal. I only suggest one minor clarification, which could be added to the Supplementary Materials section called "Supplementary Note 1". Please clarify whether the permutation test was performed by rerunning the full LOSO-CV pipeline for each permutation, including foldwise FOI selection, and confirm that no other preprocessing or model-selection steps were carried out outside the training folds. This would improve the transparency of the validation procedure.

Response:

We thank the reviewer for this important request for clarification regarding the permutation procedure and potential information leakage in the validation pipeline.

We have revised the Supplementary Note 1 to explicitly clarify the implementation details.

Specifically, we confirm that the entire LOSO-CV pipeline was fully re-executed for each permutation, including foldwise FOI selection, feature extraction, and model training, with class labels randomly shuffled at the dyad level. Importantly, all preprocessing steps, statistical testing, and model fitting were strictly confined to the training folds in each iteration, ensuring complete independence of the held-out test dyad.

No preprocessing steps, selected features, or model parameters were carried over between permutations or across folds. This has been explicitly clarified in the revised Supplementary Materials to improve transparency and reproducibility of the validation procedure.

Please see the revised Supplementary Note 1, Page 2, Lines 9 – end.

Supplementary Note 1: In each iteration of the cross-validation, data from one dyad was held out as the test set. Using the remaining N−1 dyads, paired-samples t-tests were conducted across the full frequency range (0.01–1 Hz) to compare neural activity between the task and baseline periods. Frequency bins that exhibited significant differences after FDR correction ($p < 0.05$) were identified as the FOI for that specific fold. Subsequently, the average INS values across all channels within this dynamically selected FOI were extracted for the excluded dyad. Based on the INS features, a Logistic Regression (LR) model was trained on the N−1 dyads to distinguish between the frustration condition (FC = 1) and the control condition (CC = 0). The trained model was then utilized to predict the category of the held-out dyad. This procedure was repeated N times until every dyad had served as the test set. Importantly, we confirm that all preprocessing, feature selection (i.e., the foldwise dynamic FOI identification), and model training steps were strictly restricted to and performed within the training folds (N−1 dyads) of each iteration, ensuring complete independence of the held-out test dyad and preventing any data leakage or double-dipping.

Classification performance was evaluated using prediction accuracy and the Area Under the ROC Curve (AUC), with a 1000-iteration permutation test performed to determine if the performance significantly exceeded chance levels. To ensure a rigorous and unbiased validation, the full LOSO-CV pipeline was completely rerun from scratch for each of the 1000 permutations. Specifically, for each permutation, the class labels were randomly shuffled, and the entire pipeline—including the foldwise paired-samples t-tests, dynamic FOI selection, and model training—was executed anew using the permuted labels, with no information or selected parameters carried over from the original true-label analysis.

Furthermore, to evaluate the stability of the frequency selection across folds, we generated binary heatmaps of the selected frequency bins for each iteration and calculated a frequency selection density curve. This allowed for a quantitative comparison between the cross-validated frequency bins and our initially identified FOI (0.12–0.21 Hz), ensuring the robustness and consistency of our results.

The results demonstrated that: (1) the FOI selected in each fold was consistently centered on the 0.12–0.21 Hz frequency band; (2) the classification accuracy and AUC of the Logistic Regression model across all folds were significantly higher than chance levels (Accuracy = 0.771, Precision = 0.806, Recall = 0.714, F1-score = 0.758, ROC-AUC = 0.87); and (3) the permutation test confirmed the statistical significance of the model's performance. Collectively, these findings indicate that our FOI selection procedure did not introduce double-dipping bias, and the differences between the FC and CC conditions are robust and generalizable across participants.
