# Supplementary material for: Frustration reduces interpersonal competition through dynamic interpersonal neural synchronization in dyads
Source: Commun Biol. 2026 Jun 27;9:1362. doi: 10.1038/s42003-026-10573-6 (PMC13677735; doi:10.1038/s42003-026-10573-6)
Supplement: Supplementary file 2 [file 42003_2026_10573_MOESM2_ESM.pdf]

Supplementary Materials

Frustration reduces interpersonal competition through dynamic interpersonal neural synchronization in dyads

Jingyue Liu[1,2], Yujia Meng[1], Chenyu Zhang[1], Ye Zhang[1], Yifei Zhong[1], Rui Huang[1], Yingjie Liu*

1. *School of Psychology and Mental Health, Hebei Key Laboratory of Mental Health and Brain Science, North China University of Science and Technology, Tangshan, China*
2. *Key Laboratory of Modern Teaching Technology, Ministry of Education, Shaanxi Normal University, Xi'an, China*

***Corresponding author:**

Yingjie Liu, School of Psychology and Mental Health, North China University of Science and Technology, Hebei Province, 063210, China.

Email: psyliuyingjie@163.com

Supplementary Note 1

Leave-One-Subject-Out Cross-Validation for FOI Selection

To address potential concerns regarding double-dipping in the frequency band selection process (Hakim et al., 2023), we implemented a Leave-One-Subject-Out Cross-Validation (LOSO-CV) strategy. This approach integrated frequency-of-interest (FOI) identification with classification modeling to ensure the independence of the statistical inference and validation phases.

In each iteration of the cross-validation, data from one dyad was held out as the test set. Using the remaining N−1 dyads, paired-samples t-tests were conducted across the full frequency range (0.01–1 Hz) to compare neural activity between the task and baseline periods. Frequency bins that exhibited significant differences after FDR correction ($p<0.05$) were identified as the FOI for that specific fold. Subsequently, the average INS values across all channels within this dynamically selected FOI were extracted for the excluded dyad. Based on the INS features, a Logistic Regression (LR) model was trained on the N−1 dyads to distinguish between the frustration condition (FC = 1) and the control condition (CC = 0). The trained model was then utilized to predict the category of the held-out dyad. This procedure was repeated N times until every dyad had served as the test set. Importantly, we confirm that all preprocessing, feature selection (i.e., the foldwise dynamic FOI identification), and model training steps were strictly restricted to and performed within the training folds (N−1 dyads) of each iteration, ensuring complete independence of the held-out test dyad and preventing any data-leakage or double-dipping.

Classification performance was evaluated using prediction accuracy and the Area Under the ROC Curve (AUC), with a 1000-iteration permutation test performed to determine if the performance significantly exceeded chance levels. To ensure a rigorous and unbiased validation, the full LOSO-CV pipeline was completely rerun from scratch for each of the 1000 permutations. Specifically, for each permutation, the class labels were randomly shuffled, and the entire pipeline—including the foldwise paired-samples t-tests, dynamic FOI selection, and model training—was executed

anew using the permuted labels, with no information or selected parameters carried over from the original true-label analysis.

Furthermore, to evaluate the stability of the frequency selection across folds, we generated binary heatmaps of the selected frequency bins for each iteration and calculated a frequency selection density curve. This allowed for a quantitative comparison between the cross-validated frequency bins and our initially identified FOI (0.12–0.21 Hz), ensuring the robustness and consistency of our results.

The results demonstrated that: (1) the FOI selected in each fold was consistently centered on the 0.12–0.21 Hz frequency band; (2) the classification accuracy and AUC of the Logistic Regression model across all folds were significantly higher than chance levels (Accuracy = 0.771, Precision = 0.806, Recall = 0.714, F1-score = 0.758, ROC-AUC = 0.87); and (3) the permutation test confirmed the statistical significance of the model's performance. Collectively, these findings indicate that our FOI selection procedure did not introduce double-dipping bias, and the differences between the FC and CC conditions are robust and generalizable across participants.

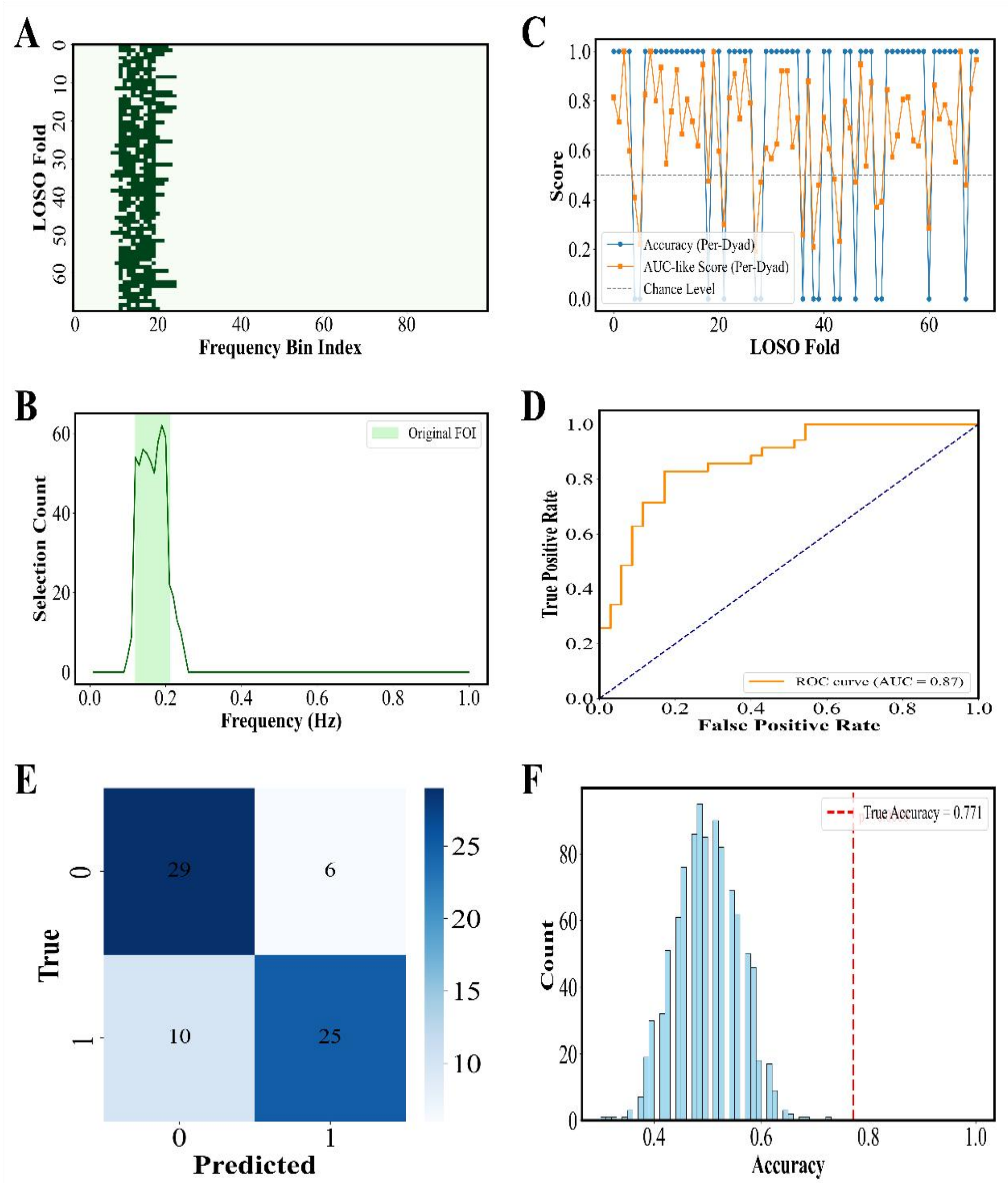

Figure S1. Leave-One-Subject-Out Cross-Validation (LOSO-CV) Analysis for FOI Selection and Model Performance. (A) Heatmap of the selected frequency bands across the 70-fold cross-validation. Each row represents one LOSO iteration, and green squares indicate the frequency bins selected as the frequency-of-interest (FOI) in that fold. (B) Frequency selection density plot. The green curve shows the frequency with which each frequency bin was selected; the light green shaded area indicates the initially identified FOI interval (0.12 – 0.21 Hz). (C) Model performance metrics for each fold. The blue line represents accuracy and the orange line represents AUC; all predicted values were significantly higher than chance

levels. (D) ROC curve of the LOSO-CV model, showing good classification capability (AUC = 0.87). (E) Confusion matrix. (F) Histogram of the permutation test with 1000 shuffled labels, where the red line represents the accuracy of the predictive model.

Supplementary Table 1. The coordinates in MNI space

CH	X	Y	Z	BA
1	53	29	36	45 - pars triangularis Broca's area, 0.50929
2	43	52	27	46 - Dorsolateral prefrontal cortex, 0.73568
3	16	69	24	10 - Frontopolar area, 1
4	-13	70	20	10 - Frontopolar area, 1
5	-38	61	10	10 - Frontopolar area, 0.63498
6	-50	46	9	45 - pars triangularis Broca's area, 0.50538
7	-57	29	14	45 -pars triangularis Broca's area, 0.92105
8	63	17	19	45 -pars opercularis, part of Broca's area, 0.50164
9	53	44	11	45 -pars triangularis Broca's area, 0.65724
10	36	66	5	10 - Frontopolar area, 0.77143
11	4	71	7	10 - Frontopolar area, 1
12	-24	70	30	10 - Frontopolar area, 0.61667
13	-42	59	0	46 - Dorsolateral prefrontal cortex, 0.56471
14	-53	42	1	45 - pars triangularis Broca's area, 0.63211
15	-58	25	9	45 - pars triangularis Broca's area, 0.62739
16	58	34	0	45 - pars triangularis Broca's area, 0.83548
17	46	57	-6	46 - Dorsolateral prefrontal cortex, 0.66802
18	21	71	-8	11 - Orbitofrontal area, 0.91554
19	-8	71	10	10 - Frontopolar area, 0.78788
20	-33	65	-9	11 - Orbitofrontal area, 0.54717
21	-47	51	-9	46 - Dorsolateral prefrontal cortex, 0.67054
22	-54	38	-5	46 - Dorsolateral prefrontal cortex,0.49498
