# Supplementary material for: Frustration reduces interpersonal competition through dynamic interpersonal neural synchronization in dyads
Source: Commun Biol. 2026 Jun 27;9:1362. doi: 10.1038/s42003-026-10573-6 (PMC13677735; doi:10.1038/s42003-026-10573-6)
Supplement: Supplementary file 3 [file 42003_2026_10573_MOESM3_ESM.pdf]

Corresponding author(s): Yingjie Liu
Last updated by author(s): Jun 10, 2026

Reporting Summary

Nature Portfolio wishes to improve the reproducibility of the work that we publish. This form provides structure for consistency and transparency in reporting. For further information on Nature Portfolio policies, see our Editorial Policies and the Editorial Policy Checklist.

Statistics

For all statistical analyses, confirm that the following items are present in the figure legend, table legend, main text, or Methods section.

n/a	Confirmed	
☐	☒	The exact sample size (*n*) for each experimental group/condition, given as a discrete number and unit of measurement
☐	☒	A statement on whether measurements were taken from distinct samples or whether the same sample was measured repeatedly
☐	☒	The statistical test(s) used AND whether they are one- or two-sided<br>*Only common tests should be described solely by name; describe more complex techniques in the Methods section.*
☒	☐	A description of all covariates tested
☐	☒	A description of any assumptions or corrections, such as tests of normality and adjustment for multiple comparisons
☐	☒	A full description of the statistical parameters including central tendency (e.g. means) or other basic estimates (e.g. regression coefficient) AND variation (e.g. standard deviation) or associated estimates of uncertainty (e.g. confidence intervals)
☐	☒	For null hypothesis testing, the test statistic (e.g. *F*, *t*, *r*) with confidence intervals, effect sizes, degrees of freedom and *P* value noted<br>*Give P values as exact values whenever suitable.*
☒	☐	For Bayesian analysis, information on the choice of priors and Markov chain Monte Carlo settings
☒	☐	For hierarchical and complex designs, identification of the appropriate level for tests and full reporting of outcomes
☐	☒	Estimates of effect sizes (e.g. Cohen's *d*, Pearson's *r*), indicating how they were calculated

Our web collection on statistics for biologists contains articles on many of the points above.

Software and code

Policy information about availability of computer code

Data collection	Two identical fNIRS systems (LIGHTNIRS; Shimadzu Co., Japan) were utilized to record the brain signals of two participants in a dyad during the experiment. The absorption values of the three wavelengths (780 nm, 805 nm, and 830 nm) of near-infrared light were measured at a sampling rate of 13.3 Hz and subsequently transformed into concentration changes of oxyhemoglobin (HbO), deoxyhemoglobin (HbR), and total hemoglobin (HbT) in accordance with the modified Beer-Lambert law (Owenreece et al., 1999).
Data analysis	Behavioral data were analyzed using SPSS 26 (IBM Corp., Armonk, NY, USA). Two-way ANOVA were used to determine the significance of the difference between the different conditions. $p < 0.05$ were considered statistically significant. Correlations between behavioral performances and neural indices were determined using Pearson's correlation. Details of behavioral and fNIRS data analysis are provided in the "Data analysis" section.<br>In the present study, the fNIRS data collected during the task and at rest were analyzed using the Matlab 2020b platform (Mathworks Inc., Natick, MA, USA). All custom scripts used in this study were written in Matlab 2020b.

For manuscripts utilizing custom algorithms or software that are central to the research but not yet described in published literature, software must be made available to editors and reviewers. We strongly encourage code deposition in a community repository (e.g. GitHub). See the Nature Portfolio guidelines for submitting code & software for further information.

Data

Policy information about availability of data

All manuscripts must include a data availability statement. This statement should provide the following information, where applicable:

- Accession codes, unique identifiers, or web links for publicly available datasets
- A description of any restrictions on data availability
- For clinical datasets or third party data, please ensure that the statement adheres to our policy

The main data used to generate all the figures and analyses in this article are available in the Science Data Bank repository: (https://doi.org/10.57760/sciencedb.15908). Individual behavioral and fNIRS raw data are available from the corresponding author upon reasonable request.

Research involving human participants, their data, or biological material

Policy information about studies with human participants or human data. See also policy information about sex, gender (identity/presentation), and sexual orientation and race, ethnicity and racism.

Reporting on sex and gender	For the present study, we collected roughly equal numbers of male and female samples. Nevertheless, we did not analyze the data for gender. This decision was largely based on the fact that the main objective of this study did not involve gender differences. To ensure data accuracy and ethical compliance, we followed strict privacy measures in collecting personal information and obtained informed consent from all participants. Participants voluntarily provided information about their biological sex for describing sample composition, but this information was not used for gender-specific analyses.In order to ensure data accuracy and ethical compliance, strict privacy measures were taken in the collection of personal information and informed consent was obtained from all participants. Participants voluntarily provided information about their biological sex through a questionnaire that was used to describe the sample composition, but this information was not used for gender-specific analyses.
Reporting on race, ethnicity, or other socially relevant groupings	The present study did not analyze data on socially constructed or socially relevant categorical variables (e.g., race, ethnicity, etc.). This decision was largely based on the fact that the main objective of this study did not involve race differences.
Population characteristics	See above
Recruitment	PARTICIPANT RECRUITMENT AND SCREENING: To ensure a diverse sample, participants were recruited through multiple channels including social media platforms, campus advertisements, and email. All participants reported unimpaired or corrected vision and had no physical or mental health problems. Prior to the start of the experiment, participants were asked to complete a short questionnaire to collect basic information such as gender, age, and past medical history. To control for the influence of potential factors on the experimental task, we used the Spielberger State Anxiety Inventory (STAI-state) (Spielberger et al., 1983) and the Beck Depression Inventory, Second Edition (BDI-II) (Beck et al., 1996) to assess the participants' mood states. Only those participants whose mood state scores were within the normal range were allowed to proceed to the follow-up task. All participants participated voluntarily and signed an informed consent form prior to the start of the experiment.
Ethics oversight	Approval Letter of Ethics Committee of North China University ofScience and Technology（2023030)

Note that full information on the approval of the study protocol must also be provided in the manuscript.

Field-specific reporting

Please select the one below that is the best fit for your research. If you are not sure, read the appropriate sections before making your selection.

☐ Life sciences ☒ Behavioural & social sciences ☐ Ecological, evolutionary & environmental sciences

For a reference copy of the document with all sections, see nature.com/documents/nr-reporting-summary-flat.pdf

Behavioural & social sciences study design

All studies must disclose on these points even when the disclosure is negative.

Study description	The present study used a quantitative experimental design aimed at exploring the dynamic effects of frustrating experiences on interpersonal competition through a systematic experimental approach.
Research sample	For this study, 146 college students with unimpaired or corrected vision and devoid of any physical or psychological health issues were enlisted. All participants were right-handed individuals. Every participant was randomly matched with an unfamiliar counterpart. Each pair, composed of two individuals of identical gender, was randomly allocated to either the Frustration condition or Control condition. Three pairs were omitted from the analyses due to lack of relative role differences, prolonged task response times, or unsuccessful fNIRS device acquisition. Consequently, the ultimate dataset comprised 35 pairs in Frustration condition (mean age = 19.45 ± 1.35 years, females = 40) and 35 pairs in Control condition (mean age = 19.60 ± 1.55 years, females = 36).

	Participants provided informed consent before the commencement of the experiment, and their remuneration was contingent upon task performance. This study adhered to the ethical guidelines delineated in the most recent Declaration of Helsinki and received approval from the local medical ethics committee. All participants provided written informed consent for the experiment.
Sampling strategy	This study used random sampling to select participants. This means that each member of the population had an equal chance of being selected into the sample, thus minimizing selection bias and increasing the external validity of the results. In addition, in this study, we did not perform a formal sample size calculation. However, we chose a sample size large enough to ensure the validity of the statistical analysis and the reliability of the results. Specifically, our sample size (146 participants) was significantly larger than those used in previous similar studies. Based on prior research experience, larger sample sizes contribute to statistical efficacy and stability of results.
Data collection	Two participants arrived at the laboratory and were ushered into a secluded room after confirming their unfamiliarity with each other. Subsequently, the two participants were positioned side-by-side at an elongated table equipped with a computer screen and two keyboards placed before them, while a partition board was inserted in the center to foster a lifelike interactive setting. Upon seating, the experimenter meticulously elucidated the entire experimental procedure to ensure comprehensive comprehension of the experiment's requisites. The experimental protocol comprised several stages, encompassing two questionnaire administration sessions (pre-frustration task: demographic questionnaire, state-check questionnaire, and Positive and Negative Affect Scale (PANAS); post-frustration task: PANAS) and two task modules (frustration-inducing task or control task; interpersonal competition task), culminating in a concluding recovery phase. Two identical fNIRS systems (LIGHTNIRS; Shimadzu Co., Japan) were utilized to record the brain signals of two participants in a dyad during the experiment.
Timing	2023.6.10-2023.10.26
Data exclusions	During data analysis, data from a total of three dyads (six participants) were excluded for the following reasons:<br>Lack of relative role differences: two participants in one dyad failed to exhibit the expected role differences, which may have affected the validity of the experimental results.<br>Excessive task response time: the task response time for one dyad was significantly outside the normal range, resulting in data that did not meet the preset analysis criteria.<br>Unsuccessful fNIRS device acquisition: a dyad's brain signal recordings were unable to obtain high-quality data due to technical problems or equipment malfunction.<br>These exclusion criteria were pre-established during the experimental design phase to ensure data quality for the final analysis and validity of the experimental results.
Non-participation	No particioants dropped out/declined particioation.
Randomization	In the present study, participants were assigned to different experimental groups through randomization. Specifically, after participants completed an initial screening and agreed to participate, they were assigned to one of the two main experimental conditions (e.g., a frustration-induction task or a control task) through a preprogrammed random assignment system, thus ensuring that each participant had an equal chance of entering either experimental condition.

Reporting for specific materials, systems and methods

We require information from authors about some types of materials, experimental systems and methods used in many studies. Here, indicate whether each material, system or method listed is relevant to your study. If you are not sure if a list item applies to your research, read the appropriate section before selecting a response.

Materials & experimental systems

n/a	Involved in the study
☒	☐ Antibodies
☒	☐ Eukaryotic cell lines
☒	☐ Palaeontology and archaeology
☒	☐ Animals and other organisms
☒	☐ Clinical data
☒	☐ Dual use research of concern
☒	☐ Plants

Methods

n/a	Involved in the study
☒	☐ ChIP-seq
☒	☐ Flow cytometry
☒	☐ MRI-based neuroimaging

Plants

Seed stocks	This study involved human participants and used functional near infrared spectroscopic imaging (fNIRS) as a neuroimaging technique. Unfortunately, the available options failed to cover the employed materials, experimental systems, and methods used in this study. Even if we had marked all inapplicable items as "n/a", this section would still have appeared.
Novel plant genotypes	This study involved human participants and used functional near infrared spectroscopic imaging (fNIRS) as a neuroimaging technique. Unfortunately, the available options failed to cover the employed materials, experimental systems, and methods used in this study. Even if we had marked all inapplicable items as "n/a", this section would still have appeared.
Authentication	This study involved human participants and used functional near infrared spectroscopic imaging (fNIRS) as a neuroimaging technique. Unfortunately, the available options failed to cover the employed materials, experimental systems, and methods used in this study. Even if we had marked all inapplicable items as "n/a", this section would still have appeared.
